# Community health workers in humanitarian settings: Scoping review

**DOI:** 10.7189/jogh.10.020602

**Published:** 2020-12

**Authors:** Nathan P Miller, Farid Bagheri Ardestani, Hannah Sarah Dini, Fouzia Shafique, Nureyan Zunong

**Affiliations:** 1 UNICEF, New York, USA; 2 Columbia University Mailman School of Public Health, New York, USA; 3 Save the Children, Washington, D.C., USA

## Abstract

**Background:**

There is a need for greater understanding of experiences implementing community-based primary health care in humanitarian settings and of the adjustments needed to ensure continuation of essential services and utilization of services by the population, and to contribute to effective emergency response. We reviewed the evidence base on community health workers (CHWs) in humanitarian settings, with the goal of improving delivery of essential services to the most vulnerable populations.

**Methods:**

We conducted a scoping review of published and grey literature related to health and nutrition services provided by CHWs in humanitarian settings. Extracted data from retained documents were analyzed inductively for key themes.

**Results:**

Of 3709 documents screened, 219 were included in the review. Key findings from the literature include: 1) CHWs were often able to continue providing services during acute and protracted crises, including prolonged periods of conflict and insecurity and during population displacement. 2) CHWs carried out critical emergency response activities during acute crises. 3) Flexible funding facilitated transitions between development and humanitarian programming. 4) Communities that did not have a locally-resident CHW experienced reduced access to services when travel was limited. 5) Community selection of CHWs and engagement of respected local leaders were crucial for community trust and acceptance and high utilization of services. 6) Selection of local supervisors and use of mobile phones facilitated continued supervision. 7) Actions taken to maintain supplies included creating parallel supply chains, providing buffer stocks to CHWs, and storing commodities in decentralized locations. 8) When travel was restricted, reporting and data collection were continued using mobile phones and use of local data collectors. 9) CHWs and supervisors faced security threats and psychological trauma as a result of their work.

**Conclusions:**

To achieve impact, policy makers and program implementers will have to address the bottlenecks to CHW service delivery common in stable low-income settings as well as the additional challenges unique to humanitarian settings. Future interventions should take into account the lessons learned from years of experience with implementation of community-based primary health care in humanitarian settings. There is also a need for rigorous assessments of community-based primary health care interventions in humanitarian settings.

There is a need to assess strategies to maintain and improve access to essential health and nutrition services in humanitarian settings. Community health workers (CHWs) can provide crucial contributions to maintaining essential services [1] and to emergency response [2] during crises. Following the West Africa Ebola outbreak and with the current COVID-19 pandemic, there is a growing recognition of the importance of CHWs for health system resilience and for global health security [3,4]. However, CHWs are still not included in most countries’ emergency preparedness and response plans [5].

There is substantial evidence that CHWs can deliver a wide range of services at community level, that they can increase access to essential health care services, and that scaling up community-level interventions can lead to large improvements in reproductive, maternal, newborn, and child health [6-10]. However, the challenges to implementing community health services in a humanitarian setting can be daunting. Beyond implementation challenges usually faced in stable low-income settings, program implementers in humanitarian settings must also cope with challenges such as insecurity, large-scale population movements, destruction or theft of infrastructure and commodities, and even greater shortages of health workers.

There have been several experiences with provision of health care services by CHWs in humanitarian settings, but to date, there has been little effort to synthesize the evidence. There is a need for greater understanding of experiences in implementing community health interventions in humanitarian settings and about what adjustments need to be made to programs to ensure continuation of essential services, utilization of services by the population, and effective emergency response. We conducted a scoping review to synthesize the growing evidence base on health care service delivery through CHWs in humanitarian settings, with the goal of improving delivery of essential services to the most vulnerable populations.

## METHODS

### Objective

To map the existing evidence and to document the experiences with health and nutrition service delivery by CHWs in humanitarian settings.

### Research questions

1. What health and nutrition interventions have been delivered by CHWs in humanitarian settings?
2. How did crises impact the delivery of health and nutrition interventions by CHWs in humanitarian settings?
3. What were the major CHW service delivery bottlenecks and facilitators in humanitarian settings?
4. What CHW service delivery strategies and tools have been employed to overcome bottlenecks in humanitarian settings?
5. What were the lessons learned for overcoming bottlenecks and improving health and nutrition service delivery and improving emergency response by CHWs in humanitarian settings?

### Search strategy

We conducted a scoping review of published and grey literature following the scoping review methodology laid out by Kahlil et al. [11] and the PRISMA Extension for Scoping Reviews (PRISMA-ScR) [12]. A scoping review allowed for a comprehensive mapping and synthesis of the existing literature on CHWs in humanitarian settings and identification of key research gaps. Given the fact that no previous synthesis of this topic has been conducted, and because the existing literature is heterogeneous in nature, a scoping review that included the full scope of literature on the topic, regardless of type or quality of the study, was most appropriate [11].

### Search strategy for published literature

We searched the Medline, Scopus, Web of Science, CINAHL, PsychINFO, Africa Wide, Academic Search Premier, Health Source Nursing Academic, and EconLit databases using the Medical Subject Headings (MESH) and key words. Additionally, the bibliographies of recently published relevant reviews were reviewed in order to identify documents that may have been missed in the database search. We had no restriction on language or publication dates. The search strategy was comprehensive in order to obtain the full range of published literature. The database search strategies are presented in Appendix S1 of the **Online Supplementary Document**.

### Search strategy for grey literature

Broadcasts were sent to the members of major global health coordination groups related to health in humanitarian settings. These included the Collaboration and Resources Group for Child Health (the CORE Group), the Global Health Cluster, the Child Health Task Force, the Interagency Working Group on Reproductive Health in Crises, and the Community Health Community of Practice. Members were asked for documents, reports, and published articles that might qualify for the review. We also requested any relevant materials related to CHWs in humanitarian settings from Ministries of Health, United Nations agencies, and non-governmental organizations known to support or have supported community-based health or nutrition services in humanitarian settings.

### Screening of abstracts and full texts

All abstracts were screened by two researchers. Any inconsistencies in inclusion/exclusion decisions between the two researchers were discussed until consensus was reached. If the two screeners could not reach a consensus, a third reviewer was consulted to arbitrate. For grey literature reports that did not contain abstracts or executive summaries, full reports were screened for inclusion criteria. Full texts of articles and reports that passed the abstract screening stage were screened by one researcher.

### Inclusion criteria

Documents were included for data extraction and analysis if they met all of the following criteria:

− Refer to provision of health or nutrition services.
− Refer to services provided by CHWs at community level. The criteria for a CHW for this review were: 1) They provided health or nutrition services in the communities from which they came (including a wider area encompassing their home community); 2) Their role as a CHW was recognized by the Ministry of Health (MoH) or a partner organization; 3) They were linked to and supported by the health system (MoH or partner organization); 4) They had less training than professional health workers (below tertiary education certificate).
− Describe provision of services in a humanitarian setting. Post-conflict/post-emergency settings were considered if they were related to the period directly after the emergency and a clear link was made between the conditions described and the emergency. Emergency preparedness interventions were considered even if the study did not take place during an emergency.
− Include information from a low- or middle-income country.
− Describe a research study or a description of implementation of interventions.

### Data extraction

Full texts of journal articles and grey literature that were retained following the full-text screening were analyzed for content relevant to the research questions. Relevant text was extracted to a standardized data extraction form.

### Charting data and narrative synthesis

Using the extracted data, study characteristics and key findings for each paper were recorded in a table (Appendix S2 of the **Online Supplementary Document**). Key themes and additional findings were identified through review of the summary table and the extracted data. We used inductive analysis of the extracted data to identify key themes in the data. In addition to thematic analysis, we included points that were determined to be relevant to the research questions, even if they did not appear frequently in the data. We applied this subjective approach to allow for inclusion of the full range of experiences that may be useful to program implementers and policy makers, even if some experiences were not frequently reported.

Consistent with the methods of a scoping review [11], the objective of including the full range of relevant literature, and the qualitative nature of the analysis, quality assessment of included documents was not carried out. Furthermore, given the dearth of rigorous quantitative evaluations of interventions, the wide range of interventions described, the wide range of study methods applied in the literature, and the qualitative focus of the research questions of this scoping review, no assessment of the effectiveness of interventions was conducted.

## RESULTS

### Search results

The peer-reviewed literature search was run on October 9, 2018 and resulted in 3637 non-duplicative results. Of these, 167 met the inclusion criteria. An additional 59 documents were identified through the grey literature search, of which 44 met the inclusion criteria. Finally, 13 documents were identified through reference screening and 8 of those met the inclusion criteria. The total number of documents screened was 3709, of which 219 were included in the review. Appendix S3 of the **Online Supplementary Document** presents the flowchart for the peer-reviewed and grey literature search process.

### Characteristics of the included literature

Appendix S2 of the **Online Supplementary Document** provides a table summarizing the key information for each of the included documents and Table 1 gives a brief summary of document characteristics. The majority of the included documents were journal articles; from the Africa, Eastern Mediterranean, and South-East Asia regions; reported on a conflict-related setting or a disease outbreak setting; and detailed CHW services that were provided in a general population setting (rather than a camp setting). There was a large variety of study designs, including pre-post surveys, single cross-sectional surveys, qualitative interviews and focus group discussions, document analysis, and documentation of experiences.

**Table 1:** Characteristics of included literature

	Number
**Type of document:**	
Journal article	181
Report	16
Commentary	4
Dissertation	4
Book chapter	2
Conference abstract	2
Conference paper	2
Conference presentation	1
Magazine article	2
Web blog	5
**WHO region:**	
Africa	84
Americas	25
Eastern Mediterranean	63
Europe	1
South-East Asia	40
Western Pacific	3
Various/not specified	8
**Type of crisis:**	
Conflict	142
Disease outbreak	44
Drought	1
Natural disaster	25
Nutrition emergency	5
Various/not specified	4
Crisis setting:	
General population	182
Refugee/IDP camp	47
Various/not specified	3

### Characteristics of CHWs and services provided

Most papers did not provide detailed information on characteristics (ie, age, gender, education, remuneration, training received, etc.) of the CHWs they described. Of the ones that did provide details, we see a great deal of variation in terms of age (between 16 and 55 years of age), gender (mostly either exclusively women or mixed women and men), education and literacy (from no education and illiterate to secondary school graduates), remuneration (either volunteer or a range of stipends or salaries), and extent of integration into national health systems (supported by an NGO with no ties to the health system, fully integrated into the health system, or a combination of the two).

There was also a wide range of interventions carried out by CHWs in the literature. Common services provided by CHWs included treatment of childhood illnesses; tuberculosis (TB) case finding and treatment; vitamin A supplementation; deworming; immunization promotion or provision; malnutrition screening; assisting home deliveries; antenatal and postnatal care for women and newborns; health and nutrition education; promotion of facility delivery; promotion of family planning and provision of contraceptives; promotion of water, sanitation, and hygiene (WASH) practices; psychosocial support; trauma care; first aid; surveillance and case finding during disease outbreaks; and registration of births and deaths. The full list of services provided is shown in the table in Appendix S2 of the **Online Supplementary Document**.

### Need for community-based services

Many documents highlighted a need for community-based services because health facility services were not accessible to large populations [13-19]. During conflict in South Sudan, many health facilities were closed, looted, and/or burnt down, and health facility staff fled the affected areas. Drugs were looted or destroyed and those facilities that remained open often lacked essential medicines and supplies [18]. During the Ebola outbreak, some health facilities closed and utilization greatly decreased in facilities that remained open because of fear of the disease [20,21]. Even if health facilities remained open, they were often overwhelmed and understaffed [22].

Health services in opposition-controlled areas were neglected by governments in El Salvador [14,23], Ethiopia [24], and Myanmar [25]. In some cases, health facilities and health workers were deliberately targeted by combatants, including the national military [14,23,26,27]. This targeting led to closure of health facilities, shortages of health workers, limited mobility of health workers, difficult access to health facilities, and blocked access to communities. In response to the lack of access to facility-based care, local authorities and communities organized the provision of essential care at the community level [14,15,22,23,28].

Several studies suggested that CHW services were more resilient than health facility services during the West Africa Ebola outbreak [29], during conflict in South Sudan [18] and Yemen [30], and during a flooding disaster in Bangladesh [31]. This was due to greater accessibility of CHWs when travel was hampered [18,30,31] and because of mistrust of the government and health facilities and a greater level of trust that existed between CHWs and community members [21,29]. The limitations of an emergency response strategy that focused on outside actors delivering aid to affected communities was highlighted in the response to the 2015 earthquake in Nepal; it took an average of 19 days before any outside relief arrived to remote mountain villages, highlighting the need for sufficiently equipped locally-based service providers in hard-to-reach areas [5].

### Maintaining essential services during crises

The large majority of the literature reported that CHWs were able to continue providing services during acute crises and long periods of conflict and insecurity, although some CHWs substantially reduced their service provision in the early phases of emergencies. CHWs in Nepal continued providing services immediately after the earthquake [5]. CHWs in Bangladesh continued providing integrated community case management (iCCM) services during a flooding emergency, although the number of treatments decreased because of travel limitations. A number of coping mechanisms were employed to ensure continuation of services. For example, when community clinics were flooded or were inaccessible, CHWs visited households by boat or set up temporary clinics in accessible areas. Supervisors and community members communicated with CHWs by phone when they were unable to reach them in person [31]. During a period of acute flare-up of conflict in South Sudan in 2013-2014, a majority of CHWs continued their iCCM activities. The number of children treated declined by about 50% at the peak of the crisis, but then quickly recovered. The number of children treated by CHWs was consistently higher than by health facilities before, during, and after the crisis [18]. In Central African Republic (CAR), CHWs continued providing services over a period of more than four years of protracted conflict and insecurity. The CHW reporting rate fell from 95% to 70% during the peak of the conflict, but then recovered once the situation had stabilized [32]. In Myanmar, maternal health workers (MHWs) provided antenatal care, counseling, and delivered babies during a time of heightened insecurity when community members were displaced into the jungle [26].

There were several examples – in Myanmar [26,27,33], CAR [32], northern Uganda [34], Burundi [34], and South Sudan [18,35] – of CHWs moving with communities when populations were displaced due to conflict and continuing to provide services. In South Sudan, displaced CHWs treated the host population in addition to their displaced home community members. Likewise, CHWs in areas that received IDPs treated both their original host population and IDPs [18]. In Myanmar, military attacks forced communities into the jungle for four months. The CHWs moved with the populations and continued services throughout this period [27].

During the West Africa Ebola outbreak, CHWs often continued their work, including during the early outbreak period when they did not receive any instruction or support. In Kenema District of Sierra Leone, 95% of CHWs reported activity during the outbreak [36]. The numbers of children treated for pneumonia, diarrhea, and malaria dropped sharply during the initial period of the outbreak, but CHWs did continue providing services in many areas and services rebounded in the later stage of the outbreak [21,29,37]. In Guinea, the proportion of CHWs in Ebola-affected areas reported being active fell from 98% before the outbreak to 74% during the outbreak. The proportion managing malaria cases fell from 68% to 48% [37]. Declines in treatments occurred because CHWs were instructed to stop providing treatments, did not receive drugs, were afraid of infection, or were diverted to Ebola-related activities, and because of reduced utilization due to fear of contracting Ebola while seeking treatment [21,29]. However, when CHWs received clear instructions to continue services and were supplied, services quickly rebounded, even during the outbreak. Services that did not depend on drug supplies generally continued, demonstrating that CHWs continued to be active and continued providing their routine services that were not as dependent on outside support or supplies [29].

In an HIV/AIDS project in South Sudan, during periods of active insecurity, CHWs provided patients with a “runaway bag” containing three months of antiretroviral therapy (ART). Evaluation of the context at regular intervals allowed the team to activate the contingency plan on time, before the situation deteriorated to a point where restrictions imposed on the movements of the CHWs would make them unable to pick up the medication from the clinic [38,39].

There were some cases in which CHW services ceased or mostly ceased when an emergency occurred. For example, in Haiti, most Red Cross volunteers ceased their activities following the 2010 earthquake [40]. Likewise, the Somali Red Crescent stopped immunization activities for several years because the militant group in the area refused to allow house-to-house visits or mass public campaign activities [40]. In Ivory Coast, geographic expansion of a CHW program to a second health district was disrupted by violence linked to presidential elections [41].

### Policy and funding

A few examples of donor-funded programs highlighted the importance of flexibility in funding to facilitate transitions between development and humanitarian programming. In South Sudan, a donor agency was reluctant to continue funding a development-focused iCCM program during an escalation of conflict. This caused disruptions in the supply of commodities to CHWs during the crisis [18]. In Ethiopia, recurrent nutrition emergencies were treated as short-term emergencies. Therefore, a response was initiated each time an emergency occurred. The emergency response was dependent on humanitarian funding and the response was short-term. This caused repeated shortages in funding and delays in emergency response [42]. In Mali on the other hand, flexible funding allowed for longer-term planning so that interventions could bridge humanitarian and development contexts. Humanitarian resources were invested in health system strengthening activities, which facilitated the transition back to development programming [42]. In Yemen, although there was a need to expand the coverage area for iCCM services, the project closed in one of the two governorates after only one year because the short-term grant was not renewed. It was suggested that the best way to ensure sustainability of community health services such as iCCM would be to ensure that CHWs are integrated into the national health system and that the services they provide are supported by national policies [30].

### Distribution and workload of CHWs

CHWs in South Sudan [43] and Afghanistan [44] complained of difficulties in completing the expected number of home visits because of competing work priorities. CHWs’ catchment areas also played a role in their ability to deliver services as expected. In South Sudan, CHWs stated that a large number of tasks and few CHWs to cover the large populations were factors that limited the amount of time they were able to spend on delivering newborn care services [43]. In Yemen, CHWs were not evenly distributed among villages, and some CHWs were expected to provide services in multiple villages. This led to reduced access to care in villages that did not have a resident CHW. CHWs complained that they incurred high costs when they traveled and that seasonal flooding and insecurity limited their ability to consistently reach some villages. Study respondents suggested that to ensure equal access to services, each community should have its own resident CHW, especially in conflict settings where travel was often limited. Furthermore, in cases where CHWs had to cover multiple villages, CHWs needed a transport allowance to cover the cost of travel [30].

### Selection of CHWs

The importance of community selection of CHWs in facilitating community acceptance was highlighted in several settings [45]. There was a negative example from a Cambodian refugee camp in Thailand where the refugee community was not consulted or involved in the selection of the CHWs. As a result, the CHWs were not culturally acceptable to the refugees, who preferred older female CHWs rather than the young male CHWs who were hired. Furthermore, the CHWs were more educated and wealthier than the refugee population, which created mistrust of CHWs [46]. A study from Afghanistan highlighted the risks of relying on community leaders for the selection of CHWs, which led to nepotism and the recruitment of CHWs who were not the most appropriate candidates [47].

Education and literacy requirements in the recruitment of CHWs created challenges in some settings. In Yemen, a requirement that CHWs have secondary education led to a shortage of CHWs (female CHWs in particular) and to CHWs having to cover multiple villages, resulting in multiple service delivery challenges [30]. In South Sudan [18,48], Afghanistan [49], Myanmar [27], and Nepal [5], it was necessary to recruit illiterate or low literacy CHWs to ensure availability of CHWs in all communities. Low literacy levels of CHWs presented challenges to service delivery [15,27,48,49]. In response, special low literacy tools were developed to facilitate recording of monthly activities [49]; collection of data on pregnancies, live births, and deaths [27]; and diagnosis and treatment of childhood malnutrition [48]. Traditional birth attendants (TBAs) in South Sudan relied on facility-based workers to help them complete monthly reports [50]. A study in South Sudan showed that CHWs had high levels of accuracy in treating malnourished children with adapted low literacy tools [48].

### Training

A few papers highlighted issues with trainings for CHWs for emergency response activities coming too late to be effective. In India, CHWs were trained to provide psychosocial support to tsunami survivors. However, CHWs reported that they did not implement their training knowledge because the trainings occurred many months after the tsunami [51]. During the Ebola outbreak in Guinea, Liberia, and Sierra Leone, WHO, UNICEF, and the MoHs developed “no-touch” iCCM guidelines, which instructed CHWs to perform clinical examinations and treatment without touching the sick child and to suspend use of rapid diagnostic tests (RDTs) to diagnose malaria [52]. However, CHWs were trained on the no-touch policy only after the peak in cases had passed [21,29].

### Supervision

Several studies documented reductions in the frequency of supervision during emergencies. Reasons for this included insecurity [14,30,32,53], lack of transport and lack of payment to supervisors for transport [15], flooding of roads [18,30,31], and lack of human resources [15]. There were a number of unique challenges to supervision during the West Africa Ebola outbreak, including NGO policies restricting staff movement for safety reasons; the inability of NGO workers to enter many communities due to hostility and community resistance; the closure of health facilities due to quarantine, illness, or the death of health workers; and an overwhelming preoccupation with Ebola-related activities [29].

The major theme regarding overcoming supervision challenges was the use of local community members as CHW supervisors. Peer supervision was used to overcome human resources shortages in Afghanistan [15]. In contested regions of Myanmar where access to CHWs was restricted for outside supervisors, senior CHWs, called back pack health workers (BPHWs), provided clinical care in communities and were also responsible for supervision and providing supplies to the lower-level cadres [25,33]. In South Sudan, having CHW supervisors who were from the local communities allowed supervisors to receive guidance from local community leaders on whether it was safe to travel to certain areas. Supervisors were also able to track down displaced CHWs using their community networks [18].

In Yemen, when it was not possible to reach some areas to provide direct supervision, CHWs were contacted by mobile phone. CHWs also created a WhatsApp group to discuss problems, exchange ideas, and give each other feedback. Respondents in Yemen suggested providing CHWs with mobile phones so that all CHWs could be in contact with their supervisors when travel was restricted [30].

### Supply of commodities

Supply of commodities was a common challenge. Shortages of commodities often occurred at the central level or at health facilities [15,18,29,30,49], and commodities that were intended for CHWs were sometimes consumed at the health facility [30]. In Afghanistan [15], South Sudan [18], and Bangladesh [31], adverse weather made traveling to remote areas to deliver supplies difficult. In South Sudan [54] and Yemen [30], conflict and the need to transport commodities through insecure areas caused supply chain delays. Additional challenges included poor management, high distribution costs, and inadequate storage facilities [54]. In South Sudan [54] and Yemen [30], CHWs in areas that experienced a large influx of IDPs were not able to meet increased demand for services due to limited drug supplies. The quantity of drugs supplied to CHWs did not change from the pre-crisis levels and therefore did not take into account large increases in the population in areas that received IDPs. In Guinea, Liberia, and Sierra Leone during the Ebola outbreak, longstanding supply chain and supervision weaknesses were further exacerbated by the outbreak [29].

Program implementers did carry out several actions to improve the supply of commodities to CHWs. In South Sudan, the weakness of the government supply chain forced the implementing NGO to create a parallel supply chain [19]. Likewise, in Afghanistan, areas that were supported by NGOs were more likely to have consistent drug supplies because NGOs provided drugs outside the government system when necessary [49]. In several settings, when difficulties in delivering commodities were expected, either because of increasing insecurity or because of the arrival of inclement weather, CHWs were given increased stocks to last them through periods without resupply [15,30,32,41]. In South Sudan, supervisors began storing drug supplies in decentralized areas, such as health facilities or supervisors’ homes, because of a fear that the drug warehouse would be looted. Implementing partners and CHW supervisors suggested setting up storage facilities for drugs and supplies at the sub-district level to allow for better pre-positioning [18]. In Myanmar, CHWs suggested practical solutions to logistical constraints, including the use of walkie-talkies for communication, donkeys for travel and transport of supplies, and headlamps to facilitate provision of services [26]. In areas of El Salvador and the Philippines that were persecuted by the government, CHWs used traditional or homemade medicines to increase community self-reliance and to reduce the cost of services and the need for imported commodities [23,55,56].

### Program monitoring and data collection

In South Sudan, reporting periods were extended to allow more flexibility to account for reduced mobility [18]. In Yemen, mobile phones were used by CHWs to transmit data and by supervisors to provide feedback when travel was limited [30]. In Myanmar, BPHWs collected mortality data in areas affected by conflict that were inaccessible to outside data collectors. As described by Lee et al., “External surveyors would have to enter and travel through the conflict zones illegally at great risk, and would not have the necessary local knowledge and skills to traverse the terrain and avoid military troops. The [back pack] health workers have several advantages for conducting surveys in this population: they have a high level of familiarity with local communities, are highly trusted by the villagers, and visit all communities in the course of their normal work. These factors make them excellent surveyors for assessment of mortality and other health indicators, a task they accomplish with minimal additional resources [57].”

### Service utilization

Community members generally reported high levels of satisfaction with community-based health services, and especially valued curative services provided by CHWs [14,56]. In South Sudan, CHWs were the primary provider of curative services for sick children, with far greater numbers of sick child consultations than in health facilities [18]. On the other hand, some community members did have concerns about CHW illiteracy, competence, and lack of medical qualifications, and in some cases surveyed community members were not aware of the CHWs in their community or were unaware of the services that CHWs provided [15,30,34].

Conflict had both negative and positive effects on service utilization. For example, in Yemen, social mobilization activities were limited in insecure areas and some households were reluctant to open the door for CHWs because of fear of attacks [30]. On the other hand, conflict was said to increase utilization of CHWs because travel to health facilities was more difficult during times of heightened insecurity or because health facilities had been looted or destroyed [18,30].

A major factor promoting utilization of CHW services was trust in local CHWs. In Guatemala, community members said they trusted the village health workers from their communities and understood the treatments since they were communicated to them in a way they could understand [58]. In a region of India affected by ethnic conflict, the use of outreach workers from each ethnic group was key to gaining the trust of community members and in facilitating free access to all areas and patients [59]. In Chad, the use of local CHWs from the displaced population to carry out mortality surveillance in IDP camps may have helped to overcome some of the social, political, economic, and cultural barriers to collecting sensitive information [60].

During the Ebola outbreak in Guinea, Liberia, and Sierra Leone, because of CHWs’ ties to health facilities, communities displayed elevated levels of fear and mistrust toward CHWs [29,61,62]. However, the close relationships CHWs had with community members were more resilient than the relationships communities had with facility-based health workers and community members preferred to seek care from CHWs than from health facilities. Trust in CHWs as community members also helped overcome confusion and fear about Ebola and study respondents believed CHWs’ activities helped break chains of transmission [21,29,61].

In Myanmar, MHWs covered several communities and therefore were not residents of all of the communities that they served. For them to be accepted in communities and for women to trust them and use their services, it was essential to work with and build good relationships with local lay health workers and TBAs. MHWs reported that holding initial community meetings to introduce themselves and the project objectives was key to gaining acceptance in the community. In areas where these community meetings were not held, it led to delayed notification of pregnancies and occasional refusal from mothers to accept MHW services [26].

Another key factor in gaining community trust and acceptance of CHWs’ services was the engagement of respected community leaders. For example, in India, obtaining recognition by local leaders that TB was a major problem in their communities was key to gaining community compliance and utilization of TB services [59]. In Yemen, it was necessary to conduct meetings with community leaders and village heads to clarify the importance of CHW services and what the CHWs’ skills were [30]. Engagement of trusted local leaders was also crucial in gaining the trust of community members and enabling an effective response to the West Africa Ebola outbreak [21,29].

In some cases, it may also be necessary to integrate traditional practices with the CHWs’ services. In Myanmar, for example, a MHW described a case in which the family of a woman who required an urgent blood transfusion insisted on first performing a traditional religious ceremony with a healer. Although this consumed time and increased the risk of a poor outcome, it was necessary to gain acceptance of the MHW’s services [26].

Finally, utilization was also affected by the shortages of commodities reported above. Stockouts had consequences beyond the immediate inability to treat sick patients; they also led to a reduction in trust in CHWs by community members and reduced service utilization in general [30,49,54].

### DRC to health facilities

Long distances, lack of transportation, and cost of transportation were primary challenges to completing referrals [15,30,50]. In South Sudan, a failure to provide CHWs with mobile phones and money to make calls and lack of basic items like flashlights, bags, raincoats, and boots also made it more difficult for CHWs to facilitate referrals to health facilities [50].

In conflict settings, caregivers were less likely to complete referral to a health facility for multiple reasons, such as closure of facilities, drug stockouts, and insecurity [18]. In South Sudan, TBAs were afraid to escort pregnant women to health facilities because of fear of inter-tribal fighting, rape, and abduction [50]. In Guinea, Liberia, and Sierra Leone during the Ebola outbreak, community members refused referrals to health facilities because of fear of infection and would often hide from CHWs for fear of being referred to a health facility [21,29]. Even when patients did complete the referral despite all of the challenges, health facilities often lacked the staff, supplies, medicines, and equipment to adequately manage referred patients [18,30].

In Myanmar, the initial approach to addressing maternal mortality in conflict-affected areas where women did not have access to health facilities was to train TBAs to provide basic materials and educational messages on clean delivery and recognition of danger signs during pregnancy. This approach was insufficient as women still required access to emergency obstetric care and were unable to reach health facilities. Therefore, a project was designed that offered community-based basic emergency obstetric care [27].

### Community engagement and ownership

As discussed above, community-led selection of CHWs and directly engaging trusted local leaders and community groups was key in gaining the trust of communities to ensure service utilization [21,26,29,30,45,46,59]. This was especially true in settings where trust in government was low and services were sensitive [29,63]. In northern Nigeria, engagement of traditional and religious leaders reduced resistance to the poliovirus vaccine [64]. In Afghanistan, involvement of religious and other community leaders was important for gaining community trust and obtaining permission for CHWs to give injectable contraceptives [65].

Strong community ownership promoted community support for CHWs. For example, in El Salvador and Ethiopia, communities agreed to cover transportation costs and other expenses for CHWs [14,66]. In the Philippines, community members paid for the CHWs’ training expenses, as well as in-kind contributions to CHWs [55].

The existence of community health committees was another factor that facilitated CHW programs. In Afghanistan, village councils supported health post construction, community mobilization for campaigns and utilization of CHW services, transportation for referrals, security for female CHWs, in-kind contributions, and problem solving. Without the support of village councils, CHWs would not have been able to talk about sensitive topics, such as contraception [15,49]. In Guinea, Liberia, and Sierra Leone during the Ebola outbreak, communities that had community health committees prior to Ebola were able to respond to Ebola more quickly and effectively [29,63].

A study in Iraq highlighted the importance of ensuring that the services provided by CHWs were aligned with the needs and priorities of the community. Village paramedics and lay first responders were trained to provide care for landmine victims. However, community members wanted the paramedics and first responders to address issues beyond landmine injuries, and expanding the program’s scope was important for gaining the trust of the communities. The program also increased the feeling among community members that they were important to the external society [67].

Strong community ownership may also have had benefits beyond improved health. In Guatemala, village health workers were involved in activities outside of health, such as agriculture, land tenure, water, and sanitation, and were encouraged to promote community empowerment through addressing social determinants of health. The author who documented this reported that the program helped make “the Indian population in this part of the Guatemalan Highlands increasingly conscious of their own collective situation,” which in turn encouraged “a spirit of self-health and cooperation [58].”

### Motivation and retention of CHWs

Financial compensation was a crucial factor for motivating and retaining CHWs and for the sustainability of programs [15,49]. CHWs who were not remunerated for their work had difficulty meeting their basic needs and some CHWs went on strike to protest the lack of salary [50,68]. Some CHWs reported that their main motivation for continuing work during emergencies was a desire to help their community [5,62]. Community acceptance and appreciation of services were also important in motivating CHWs, and the establishment of CHWs’ day in Afghanistan was seen as a powerful motivator for CHWs [15]. Reasons for leaving the position included family opposition to deployment, overwhelming responsibilities beyond work duties, discrimination, lack of incentives, spouse relocation, and seeking better paid/urban employment [45].

### Security of CHWs and supervisors

CHWs and supervisors faced a number of risks related to their work responsibilities, particularly because of the need to travel frequently on dangerous roads [23,44,69] and the possession of medical commodities [23,55]. In some cases, CHWs were explicit targets because of their perceived alignment with one of the sides in a conflict. In the Philippines, CHWs were harassed and targeted by the government military and vigilante groups that were fighting against a guerrilla insurgency. Any persons, including CHWs, providing services in affected areas were assumed to be communist sympathizers [55]. In Myanmar, CHWs faced risks from the government military forces, militia checkpoints, landmines, and adverse weather [25,33]. In Nicaragua, health workers, including CHWs, were targeted by militias [70]. One study In Nicaragua found that, “Of the 20 brigadistas [CHWs]..., ten have been kidnapped and five have quit due to the threat [71].” In Guatemala, many CHWs and members of their families were targeted and killed even though they were not involved in armed activities [58]. In El Salvador, the military blocked health workers from entering opposition-controlled areas because they suspected the medicines they were carrying would be used by the guerrillas. They also blocked basic commodities such as rice, cooking oil, powdered milk, and salt and interfered with vaccination campaigns [72]. In Pakistan, community vaccination workers were targeted by militants, with many having been killed [16,73]. Insecurity caused challenges in recruitment, retention, supervision, and the ability of CHWs to travel to households [14,32,45,53]. In some cases, it was difficult or impossible to deploy CHWs in the most dangerous areas [74].

Even in non-conflict settings, CHWs may face high risks. During Ebola, CHWs were at increased risk of infection because of their contact with sick people in the community and the lack of safety equipment, such as personal protective equipment and non-contact thermometers [29,75]. In Kakuma refugee camp in Kenya, trained TBAs faced harassment and threats by untrained TBAs who felt that the trained TBAs threatened their livelihoods [76].

Some mitigation strategies were enacted or proposed to improve the safety of CHWs and supervisors. In Pakistan, security was improved with police guarding the vaccination teams [73]. In Afghanistan [74], northern Nigeria [77], and Yemen [30], government officials, NGOs, and CHWs coordinated with local communities and community leaders to ensure that community leaders took the responsibility to ensure the safety of CHWs, to facilitate communication with community members, and to warn health workers when it was not safe to travel to communities. To avoid threats to female CHWs who had to travel to other villages, male and female CHWs in Afghanistan and Yemen were grouped together to travel to some areas [15,30]. In Afghanistan [74] and Yemen [30], it was recommended to decrease the ratio of CHWs to population in insecure areas and to deploy CHWs in each village. These measures would reduce the need for CHWs to travel outside of their home communities, thereby reducing the risks they faced while traveling. Supervisors in South Sudan used their local networks in communities to find out when it was safe to travel to certain areas [18]. In Yemen, when it was not safe for supervisors to travel to the villages to supervise CHWs, they would carry out remote supervision and collect data via WhatsApp [30]. In El Salvador, CHWs traveled at night and on inconspicuous public transport, used unmarked worksites and private homes and concealed medical commodities, and used traditional and home-made medicines (such as home-made oral rehydration solution). NGOs negotiated access to affected areas and safe passage for health workers and United Nations-negotiated cease-fires allowed for vaccination campaigns [23]. Additional suggested measures to ensure the safety of CHWs and supervisors during times of increased insecurity included providing security training to CHWs [30,78] and offering insurance to CHWs [78].

### Mental health of CHWs

In addition to the physical threats faced by CHWs, some also experienced psychological trauma. CHWs in Nepal expressed ongoing anxiety and a fear of resuming their work after the earthquake [5]. A study in Indonesia found that Red Cross volunteers who responded to an earthquake experienced trauma from the earthquake, as well as additional trauma as volunteers, which led to high levels of posttraumatic stress disorder [79]. Variables associated with symptoms of mental health disorders were loss of resources, providing psychosocial support, handling administration, handing out food aid, and perceived lack of support from team leaders and the organization [80]. In Sierra Leone during the Ebola outbreak, CHWs reported sadness, grief, and loneliness stemming from the “loss of loved ones, personal fear of contracting Ebola, concern that the health system had forgotten them, and frustration with the way Ebola imposed a standstill on life in general [62].” Sources of distress for BPHWs in Myanmar included insecurity and threats of violence from the Burmese army, fear of landmines, feelings of incompetence, lack of medical resources, transportation barriers, personnel shortages, and being away from families for extended periods of time [81,82].

Suggested measures to improve the mental health of CHWs included managing working hours, having CHWs rotate between tasks that provide low and high reward, supplementing lost resources, providing proper equipment, strengthening organizational support, and providing psychosocial support to volunteers who experience traumatic situations [78,80]

### Gender

Female CHWs were generally seen as more appropriate and effective for providing maternal and child health tasks [49] and the recruitment of female CHWs was seen as an important step in improving access to health services for women [40]. In Afghanistan, the presence of a female CHW in the community was associated with higher use of modern contraception, antenatal care services, and skilled birth attendants, while the presence of a male CHW was not [83]. However, it was sometimes difficult for women to carry out other tasks that were seen as more traditionally appropriate for men [49].

Female CHWs faced a number of challenges related to their gender. Although women were often prioritized for CHW recruitment it was often difficult to find women who met the minimum educational/literacy requirements [15,30,45,84]. Furthermore, gender norms may have led communities in some countries to disproportionately select men as CHWs [84]. In Afghanistan, Democratic Republic of Congo, Liberia, and Sierra Leone, women often needed the permission of a male head of household to become CHWs and many unmarried female CHWs left their positions as CHWs when they got married [49,84]. These attitudes on gender made it difficult to attract and retain female CHWs [47]. It was even more difficult for women to become peer supervisors, CHW supervisors, or to obtain managerial or policy-making positions because of literacy, cultural, and safety concerns [15,49,84].

Women also faced gender-specific risks to security, especially when they were required to travel long distances in insecure settings [84]. There was also a gender aspect to vulnerability during the West Africa Ebola outbreak, where the majority of community-based responders were women [85].

Despite these challenges, having the opportunity to serve as CHWs was seen as empowering for women, allowing them to have an independent income, gain knowledge, improve their social status, and to have greater freedom to travel, as well as by prioritizing women’s health in communities [45,49,74]. Female CHWs were also seen as positive role models for women and girls in the community [74].

### Emergency response activities

In addition to continued provision of routine services during emergencies, there were a number of examples of CHWs carrying out critical emergency response activities during acute crises. After the Nepal earthquake, CHWs carried out immediate response activities in communities before the arrival of external aid and without formal instruction or support and they continued to support relief efforts once external aid arrived. They provided basic health care and first aid, distributed water purification items, assisted with transport of the severely wounded, participated in search and rescue of people who were trapped, suggested proper management of human and animal corpses, helped distribute and ration available food, aided construction of temporary shelters, salvaged useful materials from partially-collapsed houses, gave information on diarrhea prevention, and provided psychological support. CHWs also provided aid agencies with data on the number of households and individuals in the communities that allowed them to budget appropriately [5]. Similarly, after cyclone Nargis in Myanmar, CHWs provided care in their communities before any mobile teams from NGOs were able to access affected areas. CHWs were also a crucial part of the subsequent emergency relief effort at the community level, carrying out needs assessments and distributing aid based on their knowledge of the community [86]. Following the flooding in Pakistan in 2010, Lady Health Workers were among the first responders and were re-tasked from their more development-oriented work to focus on emergency response activities, such as malnutrition screening, mass immunization, de-worming, health education campaigns, and delivery of basic life-saving services [42,87]. Following the Haiti earthquake, CHWs were engaged to distribute chlorine products and safe storage containers [88] and to carry out active case finding for TB [89].

There were also several examples of CHWs performing key emergency response tasks during disease outbreaks. In Guinea, Liberia, and Sierra Leone during Ebola, CHWs were initially not systematically engaged in the Ebola response. However, CHWs took it upon themselves to liaise with health facilities and to provide Ebola prevention information in their communities. Ebola workers were recruited from outside communities and sent to communities to carry out Ebola activities, but these outsiders faced intense mistrust and were (sometimes violently) rejected from communities [29,63]. After initial failures, CHWs and other community members were recruited to carry out Ebola activities in their communities, including community education and social mobilization, contact tracing, active case finding, notifying burial teams of deaths, promotion of care seeking, informal caretaking of sick people, psychosocial support, and labor-related tasks [21,29,52,61,62,90]. In Monrovia, Liberia, a community-based surveillance intervention quickly increased the number of Ebola cases detected and isolated [63]. Similarly, during an Ebola outbreak in Uganda, Red Cross volunteers carried out disease surveillance, contact tracing, follow-up of recovered Ebola survivors, community sensitization to reduce stigma against survivors, and community education. Stakeholders reported that the volunteers’ status as community members allowed them to play a key role in passing messages to community members [91]. In the Democratic Republic of Congo during a cholera outbreak, CHWs in refugee camps carried out active case finding and conducted a mass education campaign, which was seen as key to reducing mortality [92].

CHWs also responded to nutrition emergencies in Mali and Ethiopia. In Mali, CHWs shifted their focus to nutrition activities during a nutrition emergency [42]. In Ethiopia, a policy change to allow community management of acute malnutrition led to much higher numbers of malnutrition treatments, high cure rates, and improved and faster response during frequent nutrition emergencies [42].

### Emergency preparedness

Emergency preparedness was rarely mentioned in the reviewed literature. In Nepal, CHWs had not been trained on disaster response, so CHWs did not feel that they were adequately prepared when the earthquake occurred. To be better prepared for future crises, CHWs requested training in first aid, how to build safe temporary housing, how to ensure the health of pregnant women and children during a disaster, how to prevent and manage illnesses that arise during a disaster, personal hygiene after a disaster, water purification techniques, and how to provide psychosocial support [5]. Likewise, in Mali, a lack of emergency preparedness and capacity for emergency response led to a slow response to the conflict and nutrition emergency in 2012 [42]. In Afghanistan, respondents in a study about emergency mobile units called for community volunteers to be integrated into disaster preparedness and response activities so that they could support emergency mobile units with their community knowledge and contacts [93].

There were a few examples of including CHWs in preparedness planning. In South Sudan, a contingency plan for CHWs included delivery of key messages on what to do in case of conflict [38]. In Iran, CHWs were used to carry out a community education intervention for disaster awareness and preparedness, resulting in significant increases in household awareness and readiness [94].

## DISCUSSION

The need for community-based health services in humanitarian settings is clear, particularly in the context of affected populations living outside of camp settings in rural areas. Given the difficult access to health facilities, mobile clinics have been widely used to provide services to hard-to-reach populations in humanitarian settings. However, mobile clinics are expensive, logistically challenging, and often provide infrequent and inconsistent access to care, and there is limited evidence supporting their use [95]. Furthermore, a modeling study found that a CHW strategy would be more effective for reducing mortality due to childhood pneumonia than a mobile clinic strategy [96].

Taking into account the limitations of fixed health facilities and mobile clinics, and the numerous examples of CHWs continuing to provide services and delivering emergency relief interventions, greater attention and resources should be directed to establishing and strengthening community-based primary health care in fragile and humanitarian settings. Instead of suspending CHW services during crises, it should be a priority to continue support to CHWs so they can maintain essential life-saving services when they are most needed. The observed declines in service provision in the initial phases of acute emergencies [18,29,31,32] may be alleviated through a commitment to maintain essential services at the community level and improved planning and preparedness for how to ensure continued support to CHWs.

Many of the service delivery barriers and facilitators in humanitarian settings are similar to those in non-emergency settings in low- and middle-income countries. The importance of factors such as predictable funding, community selection and ownership of CHWs, selection of locally-resident CHWs, providing services that address community priorities, engagement of community leaders and committees, community demand generation activities, remuneration of CHWs, maintaining supervision and supply chains, providing buffer stocks, limiting the distances CHWs have to travel, provision of transportation allowances to CHWs, use of simplified tools, and integrating CHWs into the health system are consistent with findings in development settings [8,10,97-102]. Because many of the service delivery challenges in humanitarian settings are related to the weakness of health systems, and because health services are also likely to be more effective and sustainable if they are integrated into national health systems, a focus on strengthening community health systems is essential [8,30,97].

The issue of recruitment of low-literacy CHWs is controversial. Higher CHW education and literacy have been correlated with improved performance [8] and recruiting low literacy CHWs creates additional operational challenges. However, in settings where it was not possible to recruit literate CHWs, low literacy CHWs have been able to provide a range of services [18,100,103-106], although more evidence is needed on their performance.

The lack of discussion on including CHWs in national or program preparedness plans is striking. Given the essential services they provide and the clear potential of CHWs as first responders in communities, the fact that they are not often included in emergency preparedness plans (if these plans exist at all) is a missed opportunity. Outlining in advance the critical procedures to be followed and supports to be provided during a crisis will increase CHWs’ ability to effectively continue their routine services during emergencies and to rapidly and effectively respond to crises.

There are several limitations to this study. First, because our objective was to document a wide range of experiences, we did not assess the quality of the studies included. Second, because of the small number of studies employing rigorous methods to evaluate interventions and the wide range of interventions and study methods, we did not estimate effectiveness of interventions delivered by CHWs in humanitarian settings. These results should be interpreted as descriptions of experiences rather than an assessment of which interventions or implementation modalities are effective. Third, because we focused on descriptive results and we included findings that may have appeared in one or a small number of papers, the results are subjective and open to bias. Fourth, we must be cognizant of the possibility of publication bias. The large majority of included papers reported a positive result (quantitatively or qualitatively) from CHW-delivered health services. However, we do not know the number of unsuccessful interventions that were not published, so the results should be taken with caution.

Although we did not assess the effectiveness of interventions or implementation modalities, these results provide a number of valuable lessons learned. Policy makers and implementers supporting CHWs in humanitarian settings should consider these lessons in the design and management of their programs.

### Lessons learned

#### Need for community-based services

There is a clear and often increased need for community-based primary health care in both acute and protracted humanitarian settings. Health facilities are not accessible to large portions of the population and often suffer from deteriorations in service provision and utilization during crises.

#### Continuation of routine services during crises

CHWs are able to continue providing services during acute crises and prolonged periods of conflict and insecurity, although in some cases with reductions in service delivery. Clear policies and continued support to CHWs are crucial to continuation of services.

#### Emergency preparedness and response

Humanitarian response agencies should take advantage of established CHW networks to facilitate the delivery of aid and CHWs should be engaged in the initial and subsequent phases of crises. CHWs can be quickly trained on new tasks to respond to a crisis. Emergency response would be improved by developing emergency preparedness plans at the community level and providing CHWs and supervisors with training on emergency preparedness and response.

#### Funding of community-based health services

Flexible and longer-term funding arrangements that allow smoother transitions between development and humanitarian programming facilitate rapid emergency response and achievement of longer-term development objectives. This is especially important in fragile settings where crises occur regularly and can be anticipated.

#### Distribution of CHWs

Communities that are not served by a CHW who is a resident of that community may experience reduced access to services, particularly during periods of heightened insecurity when travel is restricted.

#### Selection of CHWs

Community-led selection of CHWs is crucial for ensuring community acceptance of and trust in the CHW and leads to higher utilization of services. In settings where literacy levels are low, it may be necessary to recruit low-literacy CHWs, especially if it is a priority to recruit female CHWs from the community in which they serve. In this case, it is necessary to develop low-literacy tools and to provide the necessary support to CHWs to manage their tasks.

#### Supervision and program monitoring

Having supervisors who are from the same communities as the CHWs allows for greater levels of familiarity and trust among community members and enables supervisors to use local networks to obtain information on the local security situation and population movements. CHW peer supervisors may be able to carry out supervision when outside supervisors cannot reach or contact affected communities.

Where there is sufficient access to phone and data networks, mobile technology can be used to carry out supervision, transmit data, transfer payments, and to track down displaced CHWs when it is not possible for supervisors to travel to communities. For this to be feasible, all CHWs will need to have access to mobile phones and sufficient phone credit.

#### Supply chain

Implementers need to plan for periods of difficult access to communities, whether because of severe weather, insecurity, or other disruptions. This may mean pre-positioning supplies in decentralized locations that will be reachable for CHWs during crises, giving CHWs extra buffer stocks, and/or providing CHWs with “runaway bags” with essential medicines and supplies. As medical commodities may be a target for armed groups, storage locations should be as inconspicuous as possible. Population movements must also be taken into account when quantifying the amount of commodities to supply to CHWs. In cases where the routine supply chain has broken down, implementing partners may need to create parallel supply chains.

#### Community engagement and service utilization

In addition to use of local CHWs for service delivery and emergency response, trusted community leaders and community health committees should be engaged in order to gain acceptance from the community.

#### Referral to health facilities

Actions to facilitate referrals, such as mobilization of community transportation, providing compensation for transportation, and/or providing CHWs with the supplies needed to accompany referred patients, should be considered. In settings where referral is not possible, it may be necessary to train CHWs to provide urgent care for severely ill patients.

#### Motivation and retention of CHWs

Although many programs have operated with CHWs who were not compensated, remuneration for CHWs’ work improves motivation and retention and allows CHWs to meet their basic household needs.

#### Security of CHWs and supervisors

Measures must be put in place to reduce the risks to CHWs and supervisors, particularly in conflict-affected settings and during disease outbreaks. Mitigation strategies to be considered according to the local context include incorporating CHWs and supervisors in emergency preparedness plans, coordinating with local communities, ensuring that CHWs only work in their home communities, carrying out remote supervision and monitoring using mobile technology, ensuring that workers and stored commodities are inconspicuous, providing larger stocks of commodities to reduce the frequency of travel, providing security, negotiating safe access, pairing male and female CHWs, providing personal protective equipment during outbreaks, providing security training, and providing health insurance.

#### Mental health of CHWs

In addition to physical harm, the psychological trauma that CHWs may experience should also be considered. Efforts should be made to monitor CHWs’ mental health, to reduce sources of distress in the workplace, to provide strong organizational support, and to provide psychosocial support as needed.

#### Gender

Policy makers and program implementers should consider the impact of hiring practices on gender equity. Furthermore, efforts should be made to reduce the challenges that female CHWs face in their work because of their gender. For example, engagement of community leaders, husbands of CHWs, and communities in general may help to gain greater acceptance of and support for female CHWs’ roles and work duties. Providing financial remuneration and considering gender aspects of security will also facilitate the work of female CHWs.

#### Institutionalization of CHWs

Integration of CHWs into national health systems improves effectiveness and sustainability of CHW services and improves the support CHWs receive in emergencies.

### Research gaps

The lessons learned from the literature can provide guidance on how to improve CHW service delivery in humanitarian settings. However, much of the existing literature is descriptive. There is a large gap in the literature with regards to rigorous assessments of CHW service delivery, strategies to improve service delivery and access to services, and evaluations of the effectiveness of interventions. Priority research questions that need to be addressed are:

1. How can CHWs be rapidly located, contacted, mobilized, trained, and supplied following an acute crisis?
2. What is the effectiveness of strategies to maintain supervision, supply chain, and monitoring when travel is limited?
3. What is the quality of care/adherence to protocols delivered by CHWs in humanitarian settings?
4. What is the quality of care/adherence to protocols delivered by low literacy CHWs?
5. What proportion of patients referred by CHWs to a health facility in humanitarian settings complete the referral and what are the patient outcomes?
6. What is the effectiveness of strategies to facilitate referral of patients from the community to health facilities?
7. What is the quality of care delivered by CHWs managing severely ill patients when referral is not possible and what are the patient outcomes?
8. What is the effectiveness of strategies to improve security of CHWs and supervisors?
9. What is the burden of mental health disorders among CHWs?
10. What is the effectiveness of interventions to improve the mental health of CHWs?
11. What is the effectiveness of strategies to reduce barriers faced by female CHWs?
12. What is the effectiveness of strategies to make CHW programs more resilient during emergencies?
13. What is the cost-effectiveness of emergency response strategies that include CHWs compared to strategies focused on fixed facilities or mobile clinics that do not include CHWs?
14. What are the best practices in developing emergency preparedness plans that include CHWs?
15. How can CHWs be most effectively used in prevention and response to disease outbreaks?
16. What is the effectiveness of interventions delivered by CHWs in humanitarian settings?
17. What factors improve the effectiveness of interventions delivered by CHWs in humanitarian settings?

## CONCLUSIONS

Recent cholera and Ebola outbreaks and the COVID-19 pandemic have underscored the need for strong primary health care and coordinated emergency response at the community level. CHWs should be seen as crucial for community and health system resilience and for improved emergency preparedness and response. However, to achieve impact, policy makers and program implementers will have to address the bottlenecks to CHW service delivery common in stable low-income settings as well as the additional challenges unique to humanitarian settings. Future programs should take into account the lessons learned from years of experience with implementation of community-based primary health care in humanitarian settings. To strengthen this evidence base and further improve service delivery, there is a need for rigorous assessments of implementation, quality, utilization, equitability, coverage, and impact of community-based primary health care interventions in humanitarian settings.

## Additional material

Online Supplementary Document
