# Supplementary material for: Community health workers in humanitarian settings: Scoping review
Source: J Glob Health. 2020 Dec 6;10(2):020602. doi: 10.7189/jogh.10.020602 (PMC7719274; doi:10.7189/jogh.10.020602)

Appendix S1: Database search strategies

PubMed search

**Healthcare terms**
("health”) OR ("healthcare”) OR ("primary care") OR ("nutrition”) OR ("nutritional”) OR ("malnutrition”) OR ("family planning") OR
“Delivery of Health Care"[Mesh] OR "Primary Health Care"[Mesh] OR "Delivery of Health Care, Integrated"[Mesh] OR "Health Care Reform"[Mesh]
**AND Community terms**
("community”) OR ("communities”) OR ("community-based") OR ("community based") OR ("home-based") OR ("home based") OR ("village”)
OR ("Community Health Planning"[Mesh] OR "Community Health Services"[Mesh])
**AND Community Health Worker terms**
("community health workers") OR ("community health worker") OR ("community-based health workers") OR ("community-based health worker") OR ("community based health workers") OR ("community based health worker") OR ("community healthcare workers") OR ("community healthcare worker") OR ("community health care workers") OR ("community health care worker") OR ("community volunteers") OR ("community volunteer") OR ("community health volunteers") OR ("community health volunteer") OR ("volunteer health workers") OR ("volunteer health worker") OR ("voluntary health workers") OR ("voluntary health worker") OR ("lay health workers") OR ("lay health worker") OR ("community relays") OR ("community relay") OR ("relais communautaires") OR ("relais communautaire") OR ("lady health workers") OR ("lady health worker") OR ("lady health visitors") OR ("lady health visitor") OR ("community based distributors") OR ("community based distributor") OR ("community-based distribution") OR ("community based distribution") OR ("community-based distributors") OR ("community-based distributor") OR ("community health distributors") OR ("community health distributor") OR ("community based agents") OR ("community based agent") OR ("community-based agents") OR ("community-based agent") OR ("health extension workers") OR ("health extension worker") OR ("community health agents") OR ("community health agent") OR ("agents de santé") OR ("agent de santé") OR ("agents de sante") OR ("agent de sante") OR ("health surveillance assistants") OR ("health surveillance assistant") OR ("community health assistants") OR ("community health assistant") OR ("community health surveyors") OR ("community health surveyor") OR ("community health promoters") OR ("community health promoter") OR ("rural health auxiliaries") OR ("rural health auxiliary") OR ("community owned resource persons") OR ("community owned resource person") OR ("community-owned resource persons") OR ("community-owned resource person") OR ("health aides") OR ("health aide") OR ("allied health personnel") OR ("voluntary workers") OR ("voluntary worker") OR ("home nursing aides") OR ("home nursing aide") OR ("peer groups") OR ("peer group") OR ("doulas”) OR ("doula”) OR ("barefoot doctors") OR ("barefoot doctor") OR ("backpack health workers") OR ("backpack health worker") OR ("back pack health workers") OR ("back pack health worker") OR ("backpack health care workers") OR ("backpack health care worker") OR ("back pack health care workers") OR ("back pack health care worker") OR ("backpack healthcare workers") OR ("backpack healthcare worker") OR ("back pack healthcare workers") OR ("back pack healthcare worker") OR ("brigadistas") OR ("brigadista") OR ("community networks") OR ("community network") OR ("village polio volunteers") OR ("village polio volunteer") OR ("outreach volunteers") OR ("outreach volunteer") OR ("outreach workers") OR ("outreach worker") OR ("community networks") OR ("community network") OR ("community health nurses") OR ("community health nurse") OR ("community health officers") OR ("community health officer") OR ("village health workers") OR ("village health worker") OR ("village health volunteers") OR ("village health volunteer") OR ("malaria agents") OR ("malaria agent") OR ("community mobilizers") OR ("community mobilizer") OR ("community mobilisers") OR ("community mobiliser") OR ("polio volunteers") OR ("polio volunteer") OR ("volunteer health advisors") OR ("volunteer health advisor") OR ("village-based family planning workers") OR ("village-based family planning worker") OR ("village based family planning workers") OR ("village based family planning worker") OR ("community nutrition workers") OR ("community nutrition worker") OR ("community-based nutrition workers") OR ("community-based nutrition worker") OR ("community based nutrition workers") OR ("community based nutrition worker") OR ("community nutrition volunteers") OR ("community nutrition volunteer") OR ("community-based nutrition volunteers") OR ("community-based nutrition volunteer") OR ("community based nutrition volunteers") OR ("community based nutrition volunteer") OR ("accredited social health activists") OR ("accredited social health activist") OR ("anganwadi workers") OR ("anganwadi worker") OR ("agentes comunitários de saúde") OR ("agente comunitário de saúde") OR ("agentes comunitarios de saude") OR ("agente comunitario de saude") OR ("agentes polivalentes elementares") OR ("agente polivalente elementar") OR ("agentes comunitários de salud") OR ("agente comunitário de salud") OR ("agentes comunitarios de salud") OR ("agente comunitarios de salud") OR ("activistas") OR ("activista") OR ("barangay health workers") OR ("barangay health worker") OR ("colaboradores voluntários") OR ("colaborador voluntário") OR ("community drug distributors") OR ("community drug distributor") OR ("community health representatives") OR ("community health representative") OR ("female multipurpose health workers") OR ("female multipurpose health worker") OR ("female multi-purpose health workers") OR ("female

multi-purpose health worker") OR ("kaders") OR ("kader") OR ("promotoras") OR ("promotora") OR ("rural health motivators") OR ("rural health motivator") OR ("village drug-kit managers") OR ("village drug-kit manager") OR ("village drug kit managers") OR ("village drug kit manager") OR ("village health helpers") OR ("village health helper") OR ("traditional birth attendants") OR ("traditional birth attendant") OR ("midwives") OR ("midwife") OR ("midwifery") OR ("lay maternal health workers") OR ("lay maternal health worker")
OR "Community Health Workers"[Mesh]
**AND Humanitarian Settings terms**
("emergency”) OR ("emergencies”) OR ("humanitarian”) OR ("disaster”) OR ("disasters”) OR ("outbreak”) OR ("outbreaks”) OR ("epidemic”) OR ("epidemics”) OR ("crisis") OR ("crises”) OR ("insecurity”) OR ("war”) OR ("wars”) OR ("war-torn”) OR ("warfare”) OR ("conflict") OR ("conflicts") OR ("tsunami") OR ("tsunamis") OR ("flooding”) OR ("flood”) OR ("floods”) OR ("cyclone”) OR ("cyclones”) OR ("hurricane”) OR ("hurricanes”) OR ("earthquake”) OR ("earthquakes”) OR ("drought”) OR ("droughts”) OR ("famine”) OR ("famines”) OR ("refugee”) OR ("refugees”) OR ("displaced”) OR ("displacement")
OR "Relief Work"[Mesh] OR "Warfare"[Mesh] OR "Emergency Medical Services"[Mesh] OR "Disease Outbreaks"[Mesh] OR "Disasters"[Mesh] OR "Starvation"[Mesh] OR "Refugees"[Mesh] OR "Geological Phenomena"[Mesh] OR "Warfare and Armed Conflicts"[Mesh]
**AND Low- and Middle-Income Country terms**
("Afghanistan") OR ("Albania") OR ("Algeria") OR ("American Samoa") OR ("Angola") OR ("Armenia") OR ("Azerbaijan") OR ("Bangladesh") OR ("Belarus") OR ("Byelarus") OR ("Belorussia") OR ("Belize") OR ("Benin") OR ("Bhutan") OR ("Bolivia") OR ("Bosnia") OR ("Botswana") OR ("Brazil") OR ("Bulgaria") OR ("Burma") OR ("Burkina Faso") OR ("Burundi") OR ("Cabo Verde") OR ("Cape Verde") OR ("Cambodia") OR ("Cameroon") OR ("Central African Republic") OR ("Chad") OR ("China") OR ("Colombia") OR ("Comoros") OR ("Comores") OR ("Comoro") OR ("Congo") OR ("Costa Rica") OR ("Côte d'Ivoire") OR ("Cuba") OR ("Djibouti") OR ("Dominica") OR ("Dominican Republic") OR ("Ecuador") OR ("Egypt") OR ("El Salvador") OR ("Eritrea") OR ("Ethiopia") OR ("Fiji") OR ("Gabon") OR ("Gambia") OR ("Gaza") OR ("Georgia") OR ("Georgia Republic") OR ("Ghana") OR ("Grenada") OR ("Grenadines") OR ("Guatemala") OR ("Guinea") OR ("Guinea Bissau") OR ("Guyana") OR ("Haiti") OR ("Herzegovina") OR ("Hercegovina") OR ("Honduras") OR ("India") OR ("Indonesia") OR ("Iran") OR ("Iraq") OR ("Jamaica") OR ("Jordan") OR ("Kazakhstan") OR ("Kenya") OR ("Kiribati") OR ("Korea") OR ("Kosovo") OR ("Kyrgyz") OR ("Kirghizia") OR ("Kirghiz") OR ("Kyrgyzstan") OR ("Lao PDR") OR ("Laos") OR ("Lebanon") OR ("Lesotho") OR ("Liberia") OR ("Libya") OR ("Macedonia") OR ("Madagascar") OR ("Malawi") OR ("Malay") OR ("Malaya") OR ("Malaysia") OR ("Maldives") OR ("Mali") OR ("Marshall Islands") OR ("Mauritania") OR ("Mauritius") OR ("Mexico") OR ("Micronesia") OR ("Moldova") OR ("Mongolia") OR ("Montenegro") OR ("Morocco") OR ("Mozambique") OR ("Myanmar") OR ("Namibia") OR ("Nepal") OR ("Nicaragua") OR ("Niger") OR ("Nigeria”) OR (“Pakistan”) OR (“Palau") OR ("Panama") OR ("Papua New Guinea") OR (“Paraguay") OR ("Peru”) OR (“Philippines") OR ("Principe") OR ("Romania") OR ("Rwanda") OR ("Ruanda") OR ("Samoa") OR ("Sao Tome") OR ("Senegal") OR ("Serbia") OR ("Sierra Leone") OR ("Solomon Islands") OR ("Somalia") OR (“South Africa") OR ("South Sudan") OR ("Sri Lanka") OR ("St Lucia") OR ("St Vincent") OR ("Sudan") OR ("Surinam") OR ("Suriname") OR ("Swaziland") OR ("Syria") OR ("Syrian Arab Republic") OR ("Tajikistan") OR ("Tadzhikistan") OR ("Tajikistan") OR ("Tadzhik") OR ("Tanzania") OR ("Thailand") OR ("Timor") OR ("Togo") OR ("Tonga") OR ("Tunisia") OR ("Turkey") OR ("Turkmen") OR ("Turkmenistan") OR ("Tuvalu") OR ("Uganda") OR ("Ukraine") OR ("Uzbek") OR ("Uzbekistan") OR ("Vanuatu") OR ("Vietnam") OR ("West Bank") OR ("Yemen") OR ("Zambia") OR ("Zimbabwe") OR ("Deprived Countries") OR ("Deprived Population") OR ("Deprived Populations") OR ("Developing Countries") OR ("Developing Country") OR ("Developing Economies") OR ("Developing Economy") OR ("Developing Nation") OR ("Developing Nations") OR ("Developing Population") OR ("Developing Populations") OR ("Developing World") OR ("LAMI Countries") OR ("LAMI Country") OR ("Less Developed Countries") OR ("Less Developed Country") OR ("Less Developed Economies”) OR ("Less Developed Nation") OR ("Less Developed Nations") OR ("Less Developed World") OR ("Lesser Developed Countries") OR ("Lesser Developed Nations") OR ("LMIC") OR ("LMICS") OR ("Low GDP") OR ("Low GNP") OR ("Low Gross Domestic") OR ("Low Gross National") OR ("Low Income Countries") OR ("Low Income Country") OR ("Low Income Economies”) OR ("Low Income Economy") OR ("Low Income Nations") OR ("Low Income Population") OR ("Low Income Populations") OR ("Lower GDP") OR ("lower gross domestic") OR ("Lower Income Countries") OR ("Lower Income Country") OR ("Lower Income Nations") OR ("Lower Income Population") OR ("Lower Income Populations") OR ("Middle Income Countries") OR ("Middle Income Country") OR ("Middle Income Economies”) OR ("Middle Income Nation") OR ("Middle Income Nations") OR ("Middle Income Population") OR ("Middle Income Populations") OR ("Poor Countries") OR ("Poor Country") OR ("Poor Economies”) OR ("Poor Economy") OR ("Poor Nation") OR ("Poor Nations") OR ("Poor Population") OR ("Poor Populations") OR ("poor world") OR ("Poorer Countries") OR ("Poorer Economies”) OR ("Poorer Economy") OR ("Poorer Nations") OR ("Poorer Population") OR ("Poorer Populations") OR ("Third World") OR ("Transitional Countries") OR ("Transitional Country") OR ("Transitional Economies") OR ("Transitional Economy") OR ("Under Developed Countries") OR ("Under Developed Country") OR ("under developed nations") OR ("Under Developed World") OR ("Under Served Population") OR ("Under Served Populations") OR ("Underdeveloped Countries") OR ("Underdeveloped Country") OR ("underdeveloped economies") OR ("underdeveloped nations") OR ("underdeveloped population") OR ("Underdeveloped World") OR ("Underserved Countries") OR ("Underserved Nations") OR ("Underserved Population") OR

("Underserved Populations")
**NOT High Income Country terms**
NOT "Aruba"[Mesh] OR "Australia"[Mesh] OR "South Australia"[Mesh] OR "Western Australia"[Mesh] OR "Austria"[Mesh] OR "Bahamas"[Mesh] OR "Bahrain"[Mesh] OR "Belgium"[Mesh] OR "Bermuda"[Mesh] OR "Brunei"[Mesh] OR "Canada"[Mesh] OR "Channel Islands"[Mesh] OR "Denmark"[Mesh] OR "Finland"[Mesh] OR "France"[Mesh] OR "Germany"[Mesh] OR "Germany, West"[Mesh] OR "Germany, East"[Mesh] OR "Greenland"[Mesh] OR "Guam"[Mesh] OR "Hong Kong"[Mesh] OR "Iceland"[Mesh] OR "Ireland"[Mesh] OR "United Kingdom"[Mesh] OR "Israel"[Mesh] OR "Italy"[Mesh] OR "Japan"[Mesh] OR "Kuwait"[Mesh] OR "Luxembourg"[Mesh] OR "Netherlands"[Mesh] OR "New Zealand"[Mesh] OR "Norway"[Mesh] OR "Qatar"[Mesh] OR "Saudi Arabia"[Mesh] OR "Singapore"[Mesh] OR "Spain"[Mesh] OR "Sweden"[Mesh] OR "Switzerland"[Mesh] OR "Taiwan"[Mesh] OR "United Arab Emirates"[Mesh] OR "United States"[Mesh] OR "United States Virgin Islands"[Mesh]

Scopus search

**Healthcare terms**
TITLE-ABS-KEY ("health") OR ("healthcare") OR ("primary care") OR ("nutrition") OR ("nutritional") OR ("malnutrition") OR ("family planning") OR ("primary health care") OR ("primary healthcare") OR ("community health services")
**AND Community terms**
TITLE-ABS-KEY ("community") OR ("communities") OR ("community-based") OR ("community based") OR ("home-based") OR ("home based") OR ("village")
**AND Community Health Worker terms**
TITLE-ABS-KEY ("community health workers") OR ("community health worker") OR ("community-based health workers") OR ("community-based health worker") OR ("community based health workers") OR ("community based health worker") OR ("community healthcare workers") OR ("community healthcare worker") OR ("community health care workers") OR ("community health care worker") OR ("community volunteers") OR ("community volunteer") OR ("community health volunteers") OR ("community health volunteer") OR ("volunteer health workers") OR ("volunteer health worker") OR ("voluntary health workers") OR ("voluntary health worker") OR ("lay health workers") OR ("lay health worker") OR ("community relays") OR ("community relay") OR ("relais communautaires") OR ("relais communautaire") OR ("lady health workers") OR ("lady health worker") OR ("lady health visitors") OR ("lady health visitor") OR ("community based distributors") OR ("community based distributor") OR ("community-based distribution") OR ("community based distribution") OR ("community-based distributors") OR ("community-based distributor") OR ("community health distributors") OR ("community health distributor") OR ("community based agents") OR ("community based agent") OR ("community-based agents") OR ("community-based agent") OR ("health extension workers") OR ("health extension worker") OR ("community health agents") OR ("community health agent") OR ("agents de santé") OR ("agent de santé") OR ("agents de sante") OR ("agent de sante") OR ("health surveillance assistants") OR ("health surveillance assistant") OR ("community health assistants") OR ("community health assistant") OR ("community health surveyors") OR ("community health surveyor") OR ("community health promoters") OR ("community health promoter") OR ("rural health auxiliaries") OR ("rural health auxiliary") OR ("community owned resource persons") OR ("community owned resource person") OR ("community-owned resource persons") OR ("community-owned resource person") OR ("health aides") OR ("health aide") OR ("allied health personnel") OR ("voluntary workers") OR ("voluntary worker") OR ("home nursing aides") OR ("home nursing aide") OR ("peer groups") OR ("peer group") OR ("doulas") OR ("doula") OR ("barefoot doctors") OR ("barefoot doctor") OR ("backpack health workers") OR ("backpack health worker") OR ("back pack health workers") OR ("back pack health worker") OR ("backpack health care workers") OR ("backpack health care worker") OR ("back pack health care workers") OR ("back pack health care worker") OR ("backpack healthcare workers") OR ("backpack healthcare worker") OR ("back pack healthcare workers") OR ("back pack healthcare worker") OR ("brigadistas") OR ("brigadista") OR ("community networks") OR ("community network") OR ("village polio volunteers") OR ("village polio volunteer") OR ("outreach volunteers") OR ("outreach volunteer") OR ("outreach workers") OR ("outreach worker") OR ("community networks") OR ("community network") OR ("community health nurses") OR ("community health nurse") OR ("community health officers") OR ("community health officer") OR ("village health workers") OR ("village health worker") OR ("village health volunteers") OR ("village health volunteer") OR ("malaria agents") OR ("malaria agent") OR ("community mobilizers") OR ("community mobilizer") OR ("community mobilisers") OR ("community mobiliser") OR ("polio volunteers") OR ("polio volunteer") OR ("volunteer health advisors") OR ("volunteer health advisor") OR ("village-based family planning workers") OR ("village-based family planning worker") OR ("village based family planning workers") OR ("village based family planning worker") OR ("community nutrition workers") OR ("community nutrition worker") OR ("community-based nutrition workers") OR ("community-based nutrition worker") OR ("community based nutrition workers") OR ("community based nutrition worker") OR ("community nutrition volunteers") OR ("community nutrition volunteer") OR ("community-based nutrition volunteers") OR ("community-based nutrition volunteer") OR ("community based nutrition volunteers") OR ("community based nutrition volunteer") OR

("accredited social health activists") OR ("accredited social health activist") OR ("anganwadi workers") OR ("anganwadi worker") OR ("agentes comunitários de saúde") OR ("agente comunitário de saúde") OR ("agentes comunitarios de saude") OR ("agente comunitario de saude") OR ("agentes polivalentes elementares") OR ("agente polivalente elementar") OR ("agentes comunitários de salud") OR ("agente comunitário de salud") OR ("agentes comunitarios de salud") OR ("agente comunitarios de salud") OR ("activistas") OR ("activista") OR ("barangay health workers") OR ("barangay health worker") OR ("colaboradores voluntários") OR ("colaborador voluntário") OR ("community drug distributors") OR ("community drug distributor") OR ("community health representatives") OR ("community health representative") OR ("female multipurpose health workers") OR ("female multipurpose health worker") OR ("female multi-purpose health workers") OR ("female multi-purpose health worker") OR ("kaders") OR ("kader") OR ("promotoras") OR ("promotora") OR ("rural health motivators") OR ("rural health motivator") OR ("village drug-kit managers") OR ("village drug-kit manager") OR ("village drug kit managers") OR ("village drug kit manager") OR ("village health helpers") OR ("village health helper") OR ("traditional birth attendants") OR ("traditional birth attendant") OR ("midwives") OR ("midwife") OR ("midwifery") OR ("lay maternal health workers") OR ("lay maternal health worker")
**AND Humanitarian Settings terms**
TITLE-ABS-KEY ("emergency”) OR ("emergencies”) OR ("humanitarian”) OR ("disaster”) OR ("disasters”) OR ("outbreak”) OR ("outbreaks”) OR ("epidemic”) OR ("epidemics”) OR ("crisis") OR ("crises”) OR ("insecurity”) OR ("war”) OR ("wars”) OR ("war-torn”) OR ("warfare”) OR ("conflict") OR ("conflicts") OR ("tsunami") OR ("tsunamis") OR ("flooding”) OR ("flood”) OR ("floods”) OR ("cyclone”) OR ("cyclones”) OR ("hurricane”) OR ("hurricanes”) OR ("earthquake”) OR ("earthquakes”) OR ("drought”) OR ("droughts”) OR ("famine”) OR ("famines”) OR ("refugee”) OR ("refugees”) OR ("displaced”) OR ("displacement") OR (“armed conflict”)
**AND Low- and Middle-Income Country terms**
TITLE-ABS-KEY ("Afghanistan") OR ("Albania") OR ("Algeria") OR ("American Samoa") OR ("Angola") OR ("Armenia") OR ("Azerbaijan") OR ("Bangladesh") OR ("Belarus") OR ("Byelarus") OR ("Belorussia") OR ("Belize") OR ("Benin") OR ("Bhutan") OR ("Bolivia") OR ("Bosnia") OR ("Botswana") OR ("Brazil") OR ("Bulgaria") OR ("Burma") OR ("Burkina Faso") OR ("Burundi") OR ("Cabo Verde") OR ("Cape Verde") OR ("Cambodia") OR ("Cameroon") OR ("Central African Republic") OR ("Chad") OR ("China") OR ("Colombia") OR ("Comoros") OR ("Comores") OR ("Comoro") OR ("Congo") OR ("Costa Rica") OR ("Côte d'Ivoire") OR ("Cuba") OR ("Djibouti") OR ("Dominica") OR ("Dominican Republic") OR ("Ecuador") OR ("Egypt") OR ("El Salvador") OR ("Eritrea") OR ("Ethiopia") OR ("Fiji") OR ("Gabon") OR ("Gambia") OR ("Gaza") OR ("Georgia") OR ("Georgia Republic") OR ("Ghana") OR ("Grenada") OR ("Grenadines") OR ("Guatemala") OR ("Guinea") OR ("Guinea Bissau") OR ("Guyana") OR ("Haiti") OR ("Herzegovina") OR ("Hercegovina") OR ("Honduras") OR ("India") OR ("Indonesia") OR ("Iran") OR ("Iraq") OR ("Jamaica") OR ("Jordan") OR ("Kazakhstan") OR ("Kenya") OR ("Kiribati") OR ("Korea") OR ("Kosovo") OR ("Kyrgyz") OR ("Kirghizia") OR ("Kirghiz") OR ("Kyrgyzstan") OR ("Lao PDR") OR ("Laos") OR ("Lebanon") OR ("Lesotho") OR ("Liberia") OR ("Libya") OR ("Macedonia") OR ("Madagascar") OR ("Malawi") OR ("Malay") OR ("Malaya") OR ("Malaysia") OR ("Maldives") OR ("Mali") OR ("Marshall Islands") OR ("Mauritania") OR ("Mauritius") OR ("Mexico") OR ("Micronesia") OR ("Moldova") OR ("Mongolia") OR ("Montenegro") OR ("Morocco") OR ("Mozambique") OR ("Myanmar") OR ("Namibia") OR ("Nepal") OR ("Nicaragua") OR ("Niger") OR ("Nigeria”) OR (“Pakistan”) OR (“Palau") OR ("Panama") OR ("Papua New Guinea") OR (“Paraguay") OR ("Peru”) OR (“Philippines") OR ("Principe") OR ("Romania") OR ("Rwanda") OR ("Ruanda") OR ("Samoa") OR ("Sao Tome") OR ("Senegal") OR ("Serbia") OR ("Sierra Leone") OR ("Solomon Islands") OR ("Somalia") OR (“South Africa") OR ("South Sudan") OR ("Sri Lanka") OR ("St Lucia") OR ("St Vincent") OR ("Sudan") OR ("Surinam") OR ("Suriname") OR ("Swaziland") OR ("Syria") OR ("Syrian Arab Republic") OR ("Tajikistan") OR ("Tadzhikistan") OR ("Tajikistan") OR ("Tadzhik") OR ("Tanzania") OR ("Thailand") OR ("Timor") OR ("Togo") OR ("Tonga") OR ("Tunisia") OR ("Turkey") OR ("Turkmen") OR ("Turkmenistan") OR ("Tuvalu") OR ("Uganda") OR ("Ukraine") OR ("Uzbek") OR ("Uzbekistan") OR ("Vanuatu") OR ("Vietnam") OR ("West Bank") OR ("Yemen") OR ("Zambia") OR ("Zimbabwe") OR ("Deprived Countries") OR ("Deprived Population") OR ("Deprived Populations") OR ("Developing Countries") OR ("Developing Country") OR ("Developing Economies") OR ("Developing Economy") OR ("Developing Nation") OR ("Developing Nations") OR ("Developing Population") OR ("Developing Populations") OR ("Developing World") OR ("LAMI Countries") OR ("LAMI Country") OR ("Less Developed Countries") OR ("Less Developed Country") OR ("Less Developed Economies”) OR ("Less Developed Nation") OR ("Less Developed Nations") OR ("Less Developed World") OR ("Lesser Developed Countries") OR ("Lesser Developed Nations") OR ("LMIC") OR ("LMICS") OR ("Low GDP") OR ("Low GNP") OR ("Low Gross Domestic") OR ("Low Gross National") OR ("Low Income Countries") OR ("Low Income Country") OR ("Low Income Economies”) OR ("Low Income Economy") OR ("Low Income Nations") OR ("Low Income Population") OR ("Low Income Populations") OR ("Lower GDP") OR ("lower gross domestic") OR ("Lower Income Countries") OR ("Lower Income Country") OR ("Lower Income Nations") OR ("Lower Income Population") OR ("Lower Income Populations") OR ("Middle Income Countries") OR ("Middle Income Country") OR ("Middle Income Economies”) OR ("Middle Income Nation") OR ("Middle Income Nations") OR ("Middle Income Population") OR ("Middle Income Populations") OR ("Poor Countries") OR ("Poor Country") OR ("Poor Economies”) OR ("Poor Economy") OR ("Poor Nation") OR ("Poor Nations") OR ("Poor Population") OR ("Poor Populations") OR ("poor world") OR ("Poorer Countries") OR ("Poorer Economies”) OR ("Poorer Economy") OR ("Poorer Nations") OR ("Poorer Population") OR ("Poorer Populations") OR ("Third World") OR ("Transitional Countries") OR ("Transitional Country") OR ("Transitional Economies") OR

("Transitional Economy") OR ("Under Developed Countries") OR ("Under Developed Country") OR ("under developed nations") OR ("Under Developed World") OR ("Under Served Population") OR ("Under Served Populations") OR ("Underdeveloped Countries") OR ("Underdeveloped Country") OR ("underdeveloped economies") OR ("underdeveloped nations") OR ("underdeveloped population") OR ("Underdeveloped World") OR ("Underserved Countries") OR ("Underserved Nations") OR ("Underserved Population") OR ("Underserved Populations")
**NOT High Income Country terms**
TITLE-ABS-KEY (“Aruba") OR (“Australia") OR (“South Australia") OR (“Western Australia") OR (“Austria") OR (“Bahamas") OR (“Bahrain") OR (“Belgium") OR (“Bermuda") OR (“Brunei") OR (“Canada") OR (“Channel Islands") OR (“Denmark") OR (“Finland") OR (“France") OR (“Germany") OR (“Germany, West") OR (“Germany, East") OR (“Greenland") OR (“Guam") OR (“Hong Kong") OR (“Iceland") OR (“Ireland") OR (“United Kingdom") OR (“Israel") OR (“Italy") OR (“Japan") OR (“Kuwait") OR (“Luxembourg") OR (“Netherlands") OR (“New Zealand") OR (“Norway") OR (“Qatar") OR (“Saudi Arabia") OR (“Singapore") OR (“Spain") OR (“Sweden") OR (“Switzerland") OR (“Taiwan") OR (“United Arab Emirates") OR (“United States") OR (“United States Virgin Islands")

Web of Science search

**Healthcare terms**
TI=(”health” OR "healthcare” OR "primary care" OR "nutrition” OR "nutritional” OR "malnutrition” OR "family planning" OR "primary health care" OR "primary healthcare" OR “community health services”)
TS=(”health” OR "healthcare” OR "primary care" OR "nutrition” OR "nutritional” OR "malnutrition” OR "family planning" OR "primary health care" OR "primary healthcare" OR “community health services”)
**AND Community terms**
TI=("community” OR "communities” OR "community-based" OR "community based" OR "home-based" OR "home based" OR "village”)
TS=("community” OR "communities” OR "community-based" OR "community based" OR "home-based" OR "home based" OR "village”)
**AND Community Health Worker terms**
TI=("community health workers" OR "community health worker" OR "community-based health workers" OR "community-based health worker" OR "community based health workers" OR "community based health worker" OR "community healthcare workers" OR "community healthcare worker" OR "community health care workers" OR "community health care worker" OR "community volunteers" OR "community volunteer" OR "community health volunteers" OR "community health volunteer" OR "volunteer health workers" OR "volunteer health worker" OR "voluntary health workers" OR "voluntary health worker" OR "lay health workers" OR "lay health worker" OR "community relays" OR "community relay" OR "relais communautaires" OR "relais communautaire" OR "lady health workers" OR "lady health worker" OR "lady health visitors" OR "lady health visitor" OR "community based distributors" OR "community based distributor" OR "community-based distribution" OR "community based distribution" OR "community-based distributors" OR "community-based distributor" OR "community health distributors" OR "community health distributor" OR "community based agents" OR "community based agent" OR "community-based agents" OR "community-based agent" OR "health extension workers" OR "health extension worker" OR "community health agents" OR "community health agent" OR "agents de santé" OR "agent de santé" OR "agents de sante" OR "agent de sante" OR "health surveillance assistants" OR "health surveillance assistant" OR "community health assistants" OR "community health assistant" OR "community health surveyors" OR "community health surveyor" OR "community health promoters" OR "community health promoter" OR "rural health auxiliaries" OR "rural health auxiliary" OR "community owned resource persons" OR "community owned resource person" OR "community-owned resource persons" OR "community-owned resource person" OR "health aides" OR "health aide" OR "allied health personnel" OR "voluntary workers" OR "voluntary worker" OR "home nursing aides" OR "home nursing aide" OR "peer groups" OR "peer group" OR "doulas” OR "doula” OR "barefoot doctors" OR "barefoot doctor" OR "backpack health workers" OR "backpack health worker" OR "back pack health workers" OR "back pack health worker" OR "backpack health care workers" OR "backpack health care worker" OR "back pack health care workers" OR "back pack health care worker" OR "backpack healthcare workers" OR "backpack healthcare worker" OR "back pack healthcare workers" OR "back pack healthcare worker" OR "brigadistas" OR "brigadista" OR "community networks" OR "community network" OR "village polio volunteers" OR "village polio volunteer" OR "outreach volunteers" OR "outreach volunteer" OR "outreach workers" OR "outreach worker" OR "community networks" OR "community network" OR "community health nurses" OR "community health nurse" OR "community health officers" OR "community health officer" OR "village health workers" OR "village health worker" OR "village health volunteers" OR "village health volunteer" OR "malaria agents" OR "malaria agent" OR "community mobilizers" OR "community mobilizer" OR "community mobilisers" OR "community mobiliser" OR "polio volunteers" OR "polio volunteer" OR "volunteer health advisors" OR "volunteer health advisor" OR "village-based family planning workers" OR "village-based family planning worker" OR "village based family planning workers" OR "village based family planning worker" OR "community nutrition workers" OR "community nutrition worker" OR "community-based nutrition workers" OR "community-based nutrition worker" OR "community based nutrition workers"

OR "community based nutrition worker" OR "community nutrition volunteers" OR "community nutrition volunteer" OR "community-based nutrition volunteers" OR "community-based nutrition volunteer" OR "community based nutrition volunteers" OR "community based nutrition volunteer" OR "accredited social health activists" OR "accredited social health activist" OR "anganwadi workers" OR "anganwadi worker" OR "agentes comunitários de saúde" OR "agente comunitário de saúde" OR "agentes comunitarios de saude" OR "agente comunitario de saude" OR "agentes polivalentes elementares" OR "agente polivalente elementar" OR "agentes comunitários de salud" OR "agente comunitário de salud" OR "agentes comunitarios de salud" OR "agente comunitarios de salud" OR "activistas" OR "activista" OR "barangay health workers" OR "barangay health worker" OR "colaboradores voluntários" OR "colaborador voluntário" OR "community drug distributors" OR "community drug distributor" OR "community health representatives" OR "community health representative" OR "female multipurpose health workers" OR "female multipurpose health worker" OR "female multi-purpose health workers" OR "female multi-purpose health worker" OR "kaders" OR "kader" OR "promotoras" OR "promotora" OR "rural health motivators" OR "rural health motivator" OR "village drug-kit managers" OR "village drug-kit manager" OR "village drug kit managers" OR "village drug kit manager" OR "village health helpers" OR "village health helper" OR "traditional birth attendants" OR "traditional birth attendant" OR "midwives" OR "midwife" OR "midwifery" OR "lay maternal health workers" OR "lay maternal health worker")
TS=("community health workers" OR "community health worker" OR "community-based health workers" OR "community-based health worker" OR "community based health workers" OR "community based health worker" OR "community healthcare workers" OR "community healthcare worker" OR "community health care workers" OR "community health care worker" OR "community volunteers" OR "community volunteer" OR "community health volunteers" OR "community health volunteer" OR "volunteer health workers" OR "volunteer health worker" OR "voluntary health workers" OR "voluntary health worker" OR "lay health workers" OR "lay health worker" OR "community relays" OR "community relay" OR "relais communautaires" OR "relais communautaire" OR "lady health workers" OR "lady health worker" OR "lady health visitors" OR "lady health visitor" OR "community based distributors" OR "community based distributor" OR "community-based distribution" OR "community based distribution" OR "community-based distributors" OR "community-based distributor" OR "community health distributors" OR "community health distributor" OR "community based agents" OR "community based agent" OR "community-based agents" OR "community-based agent" OR "health extension workers" OR "health extension worker" OR "community health agents" OR "community health agent" OR "agents de santé" OR "agent de santé" OR "agents de sante" OR "agent de sante" OR "health surveillance assistants" OR "health surveillance assistant" OR "community health assistants" OR "community health assistant" OR "community health surveyors" OR "community health surveyor" OR "community health promoters" OR "community health promoter" OR "rural health auxiliaries" OR "rural health auxiliary" OR "community owned resource persons" OR "community owned resource person" OR "community-owned resource persons" OR "community-owned resource person" OR "health aides" OR "health aide" OR "allied health personnel" OR "voluntary workers" OR "voluntary worker" OR "home nursing aides" OR "home nursing aide" OR "peer groups" OR "peer group" OR "doulas” OR "doula” OR "barefoot doctors" OR "barefoot doctor" OR "backpack health workers" OR "backpack health worker" OR "back pack health workers" OR "back pack health worker" OR "backpack health care workers" OR "backpack health care worker" OR "back pack health care workers" OR "back pack health care worker" OR "backpack healthcare workers" OR "backpack healthcare worker" OR "back pack healthcare workers" OR "back pack healthcare worker" OR "brigadistas" OR "brigadista" OR "community networks" OR "community network" OR "village polio volunteers" OR "village polio volunteer" OR "outreach volunteers" OR "outreach volunteer" OR "outreach workers" OR "outreach worker" OR "community networks" OR "community network" OR "community health nurses" OR "community health nurse" OR "community health officers" OR "community health officer" OR "village health workers" OR "village health worker" OR "village health volunteers" OR "village health volunteer" OR "malaria agents" OR "malaria agent" OR "community mobilizers" OR "community mobilizer" OR "community mobilisers" OR "community mobiliser" OR "polio volunteers" OR "polio volunteer" OR "volunteer health advisors" OR "volunteer health advisor" OR "village-based family planning workers" OR "village-based family planning worker" OR "village based family planning workers" OR "village based family planning worker" OR "community nutrition workers" OR "community nutrition worker" OR "community-based nutrition workers" OR "community-based nutrition worker" OR "community based nutrition workers" OR "community based nutrition worker" OR "community nutrition volunteers" OR "community nutrition volunteer" OR "community-based nutrition volunteers" OR "community-based nutrition volunteer" OR "community based nutrition volunteers" OR "community based nutrition volunteer" OR "accredited social health activists" OR "accredited social health activist" OR "anganwadi workers" OR "anganwadi worker" OR "agentes comunitários de saúde" OR "agente comunitário de saúde" OR "agentes comunitarios de saude" OR "agente comunitario de saude" OR "agentes polivalentes elementares" OR "agente polivalente elementar" OR "agentes comunitários de salud" OR "agente comunitário de salud" OR "agentes comunitarios de salud" OR "agente comunitarios de salud" OR "activistas" OR "activista" OR "barangay health workers" OR "barangay health worker" OR "colaboradores voluntários" OR "colaborador voluntário" OR "community drug distributors" OR "community drug distributor" OR "community health representatives" OR "community health representative" OR "female multipurpose health workers" OR "female multipurpose health worker" OR "female multi-purpose health workers" OR "female multi-purpose health worker" OR "kaders" OR "kader" OR "promotoras" OR "promotora" OR "rural health motivators" OR "rural health motivator" OR "village drug-kit managers" OR "village drug-kit manager" OR "village drug kit managers" OR

"village drug kit manager" OR "village health helpers" OR "village health helper" OR "traditional birth attendants" OR "traditional birth attendant" OR "midwives" OR "midwife" OR "midwifery" OR "lay maternal health workers" OR "lay maternal health worker")
**AND Humanitarian Settings terms**
TI=("emergency" OR "emergencies" OR "humanitarian" OR "disaster" OR "disasters" OR "outbreak" OR "outbreaks" OR "epidemic" OR "epidemics" OR "crisis" OR "crises" OR "insecurity" OR "war" OR "wars" OR "war-torn" OR "warfare" OR "conflict" OR "conflicts" OR "tsunami" OR "tsunamis" OR "flooding" OR "flood" OR "floods" OR "cyclone" OR "cyclones" OR "hurricane" OR "hurricanes" OR "earthquake" OR "earthquakes" OR "drought" OR "droughts" OR "famine" OR "famines" OR "refugee" OR "refugees" OR "displaced" OR "displacement" OR "armed conflict")
TS=("emergency" OR "emergencies" OR "humanitarian" OR "disaster" OR "disasters" OR "outbreak" OR "outbreaks" OR "epidemic" OR "epidemics" OR "crisis" OR "crises" OR "insecurity" OR "war" OR "wars" OR "war-torn" OR "warfare" OR "conflict" OR "conflicts" OR "tsunami" OR "tsunamis" OR "flooding" OR "flood" OR "floods" OR "cyclone" OR "cyclones" OR "hurricane" OR "hurricanes" OR "earthquake" OR "earthquakes" OR "drought" OR "droughts" OR "famine" OR "famines" OR "refugee" OR "refugees" OR "displaced" OR "displacement" OR "armed conflict")
**AND Low- and Middle-Income Country terms**
TI=("Afghanistan" OR "Albania" OR "Algeria" OR "American Samoa" OR "Angola" OR "Armenia" OR "Azerbaijan" OR "Bangladesh" OR "Belarus" OR "Byelarus" OR "Belorussia" OR "Belize" OR "Benin" OR "Bhutan" OR "Bolivia" OR "Bosnia" OR "Botswana" OR "Brazil" OR "Bulgaria" OR "Burma" OR "Burkina Faso" OR "Burundi" OR "Cabo Verde" OR "Cape Verde" OR "Cambodia" OR "Cameroon" OR "Central African Republic" OR "Chad" OR "China" OR "Colombia" OR "Comoros" OR "Comores" OR "Comoro" OR "Congo" OR "Costa Rica" OR "Côte d'Ivoire" OR "Cuba" OR "Djibouti" OR "Dominica" OR "Dominican Republic" OR "Ecuador" OR "Egypt" OR "El Salvador" OR "Eritrea" OR "Ethiopia" OR "Fiji" OR "Gabon" OR "Gambia" OR "Gaza" OR "Georgia" OR "Georgia Republic" OR "Ghana" OR "Grenada" OR "Grenadines" OR "Guatemala" OR "Guinea" OR "Guinea Bissau" OR "Guyana" OR "Haiti" OR "Herzegovina" OR "Hercegovina" OR "Honduras" OR "India" OR "Indonesia" OR "Iran" OR "Iraq" OR "Jamaica" OR "Jordan" OR "Kazakhstan" OR "Kenya" OR "Kiribati" OR "Korea" OR "Kosovo" OR "Kyrgyz" OR "Kirghizia" OR "Kirghiz" OR "Kyrgyzstan" OR "Lao PDR" OR "Laos" OR "Lebanon" OR "Lesotho" OR "Liberia" OR "Libya" OR "Macedonia" OR "Madagascar" OR "Malawi" OR "Malay" OR "Malaya" OR "Malaysia" OR "Maldives" OR "Mali" OR "Marshall Islands" OR "Mauritania" OR "Mauritius" OR "Mexico" OR "Micronesia" OR "Moldova" OR "Mongolia" OR "Montenegro" OR "Morocco" OR "Mozambique" OR "Myanmar" OR "Namibia" OR "Nepal" OR "Nicaragua" OR "Niger" OR "Nigeria" OR "Pakistan" OR "Palau" OR "Panama" OR "Papua New Guinea" OR "Paraguay" OR "Peru" OR "Philippines" OR "Principe" OR "Romania" OR "Rwanda" OR "Ruanda" OR "Samoa" OR "Sao Tome" OR "Senegal" OR "Serbia" OR "Sierra Leone" OR "Solomon Islands" OR "Somalia" OR "South Africa" OR "South Sudan" OR "Sri Lanka" OR "St Lucia" OR "St Vincent" OR "Sudan" OR "Surinam" OR "Suriname" OR "Swaziland" OR "Syria" OR "Syrian Arab Republic" OR "Tajikistan" OR "Tadzhikistan" OR "Tajikistan" OR "Tadzhik" OR "Tanzania" OR "Thailand" OR "Timor" OR "Togo" OR "Tonga" OR "Tunisia" OR "Turkey" OR "Turkmen" OR "Turkmenistan" OR "Tuvalu" OR "Uganda" OR "Ukraine" OR "Uzbek" OR "Uzbekistan" OR "Vanuatu" OR "Vietnam" OR "West Bank" OR "Yemen" OR "Zambia" OR "Zimbabwe" OR "Deprived Countries" OR "Deprived Population" OR "Deprived Populations" OR "Developing Countries" OR "Developing Country" OR "Developing Economies" OR "Developing Economy" OR "Developing Nation" OR "Developing Nations" OR "Developing Population" OR "Developing Populations" OR "Developing World" OR "LAMI Countries" OR "LAMI Country" OR "Less Developed Countries" OR "Less Developed Country" OR "Less Developed Economies" OR "Less Developed Nation" OR "Less Developed Nations" OR "Less Developed World" OR "Lesser Developed Countries" OR "Lesser Developed Nations" OR "LMIC" OR "LMICS" OR "Low GDP" OR "Low GNP" OR "Low Gross Domestic" OR "Low Gross National" OR "Low Income Countries" OR "Low Income Country" OR "Low Income Economies" OR "Low Income Economy" OR "Low Income Nations" OR "Low Income Population" OR "Low Income Populations" OR "Lower GDP" OR "lower gross domestic" OR "Lower Income Countries" OR "Lower Income Country" OR "Lower Income Nations" OR "Lower Income Population" OR "Lower Income Populations" OR "Middle Income Countries" OR "Middle Income Country" OR "Middle Income Economies" OR "Middle Income Nation" OR "Middle Income Nations" OR "Middle Income Population" OR "Middle Income Populations" OR "Poor Countries" OR "Poor Country" OR "Poor Economies" OR "Poor Economy" OR "Poor Nation" OR "Poor Nations" OR "Poor Population" OR "Poor Populations" OR "poor world" OR "Poorer Countries" OR "Poorer Economies" OR "Poorer Economy" OR "Poorer Nations" OR "Poorer Population" OR "Poorer Populations" OR "Third World" OR "Transitional Countries" OR "Transitional Country" OR "Transitional Economies" OR "Transitional Economy" OR "Under Developed Countries" OR "Under Developed Country" OR "under developed nations" OR "Under Developed World" OR "Under Served Population" OR "Under Served Populations" OR "Underdeveloped Countries" OR "Underdeveloped Country" OR "underdeveloped economies" OR "underdeveloped nations" OR "underdeveloped population" OR "Underdeveloped World" OR "Underserved Countries" OR "Underserved Nations" OR "Underserved Population" OR "Underserved Populations")
TS=("Afghanistan" OR "Albania" OR "Algeria" OR "American Samoa" OR "Angola" OR "Armenia" OR "Azerbaijan" OR "Bangladesh" OR "Belarus" OR "Byelarus" OR "Belorussia" OR "Belize" OR "Benin" OR "Bhutan" OR "Bolivia" OR "Bosnia" OR "Botswana" OR "Brazil" OR "Bulgaria" OR "Burma" OR "Burkina Faso" OR "Burundi" OR "Cabo Verde" OR "Cape Verde" OR "Cambodia" OR "Cameroon" OR "Central

African Republic" OR "Chad" OR "China" OR "Colombia" OR "Comoros" OR "Comores" OR "Comoro" OR "Congo" OR "Costa Rica" OR "Côte d'Ivoire" OR "Cuba" OR "Djibouti" OR "Dominica" OR "Dominican Republic" OR "Ecuador" OR "Egypt" OR "El Salvador" OR "Eritrea" OR "Ethiopia" OR "Fiji" OR "Gabon" OR "Gambia" OR "Gaza" OR "Georgia" OR "Georgia Republic" OR "Ghana" OR "Grenada" OR "Grenadines" OR "Guatemala" OR "Guinea" OR "Guinea Bissau" OR "Guyana" OR "Haiti" OR "Herzegovina" OR "Hercegovina" OR "Honduras" OR "India" OR "Indonesia" OR "Iran" OR "Iraq" OR "Jamaica" OR "Jordan" OR "Kazakhstan" OR "Kenya" OR "Kiribati" OR "Korea" OR "Kosovo" OR "Kyrgyz" OR "Kirghizia" OR "Kirghiz" OR "Kyrgyzstan" OR "Lao PDR" OR "Laos" OR "Lebanon" OR "Lesotho" OR "Liberia" OR "Libya" OR "Macedonia" OR "Madagascar" OR "Malawi" OR "Malay" OR "Malaya" OR "Malaysia" OR "Maldives" OR "Mali" OR "Marshall Islands" OR "Mauritania" OR "Mauritius" OR "Mexico" OR "Micronesia" OR "Moldova" OR "Mongolia" OR "Montenegro" OR "Morocco" OR "Mozambique" OR "Myanmar" OR "Namibia" OR "Nepal" OR "Nicaragua" OR "Niger" OR "Nigeria" OR "Pakistan" OR "Palau" OR "Panama" OR "Papua New Guinea" OR "Paraguay" OR "Peru" OR "Philippines" OR "Principe" OR "Romania" OR "Rwanda" OR "Ruanda" OR "Samoa" OR "Sao Tome" OR "Senegal" OR "Serbia" OR "Sierra Leone" OR "Solomon Islands" OR "Somalia" OR "South Africa" OR "South Sudan" OR "Sri Lanka" OR "St Lucia" OR "St Vincent" OR "Sudan" OR "Surinam" OR "Suriname" OR "Swaziland" OR "Syria" OR "Syrian Arab Republic" OR "Tajikistan" OR "Tadzhikistan" OR "Tajikistan" OR "Tadzhik" OR "Tanzania" OR "Thailand" OR "Timor" OR "Togo" OR "Tonga" OR "Tunisia" OR "Turkey" OR "Turkmen" OR "Turkmenistan" OR "Tuvalu" OR "Uganda" OR "Ukraine" OR "Uzbek" OR "Uzbekistan" OR "Vanuatu" OR "Vietnam" OR "West Bank" OR "Yemen" OR "Zambia" OR "Zimbabwe" OR "Deprived Countries" OR "Deprived Population" OR "Deprived Populations" OR "Developing Countries" OR "Developing Country" OR "Developing Economies" OR "Developing Economy" OR "Developing Nation" OR "Developing Nations" OR "Developing Population" OR "Developing Populations" OR "Developing World" OR "LAMI Countries" OR "LAMI Country" OR "Less Developed Countries" OR "Less Developed Country" OR "Less Developed Economies" OR "Less Developed Nation" OR "Less Developed Nations" OR "Less Developed World" OR "Lesser Developed Countries" OR "Lesser Developed Nations" OR "LMIC" OR "LMICS" OR "Low GDP" OR "Low GNP" OR "Low Gross Domestic" OR "Low Gross National" OR "Low Income Countries" OR "Low Income Country" OR "Low Income Economies" OR "Low Income Economy" OR "Low Income Nations" OR "Low Income Population" OR "Low Income Populations" OR "Lower GDP" OR "lower gross domestic" OR "Lower Income Countries" OR "Lower Income Country" OR "Lower Income Nations" OR "Lower Income Population" OR "Lower Income Populations" OR "Middle Income Countries" OR "Middle Income Country" OR "Middle Income Economies" OR "Middle Income Nation" OR "Middle Income Nations" OR "Middle Income Population" OR "Middle Income Populations" OR "Poor Countries" OR "Poor Country" OR "Poor Economies" OR "Poor Economy" OR "Poor Nation" OR "Poor Nations" OR "Poor Population" OR "Poor Populations" OR "poor world" OR "Poorer Countries" OR "Poorer Economies" OR "Poorer Economy" OR "Poorer Nations" OR "Poorer Population" OR "Poorer Populations" OR "Third World" OR "Transitional Countries" OR "Transitional Country" OR "Transitional Economies" OR "Transitional Economy" OR "Under Developed Countries" OR "Under Developed Country" OR "under developed nations" OR "Under Developed World" OR "Under Served Population" OR "Under Served Populations" OR "Underdeveloped Countries" OR "Underdeveloped Country" OR "underdeveloped economies" OR "underdeveloped nations" OR "underdeveloped population" OR "Underdeveloped World" OR "Underserved Countries" OR "Underserved Nations" OR "Underserved Population" OR "Underserved Populations")

Ebsco search (CINAHL, PsychINFO, Africa Wide, Academic Search Premier, Health Source Nursing Academic, and EconLit)

**Healthcare terms**
TI ("health" OR "healthcare" OR "primary care" OR "nutrition" OR "nutritional" OR "malnutrition" OR "family planning" OR "primary health care" OR "primary healthcare" OR "community health services") OR AB ("health" OR "healthcare" OR "primary care" OR "nutrition" OR "nutritional" OR "malnutrition" OR "family planning" OR "primary health care" OR "primary healthcare" OR "community health services") OR SU ("health" OR "healthcare" OR "primary care" OR "nutrition" OR "nutritional" OR "malnutrition" OR "family planning" OR "primary health care" OR "primary healthcare" OR "community health services")
**AND Community terms**
TI ("community" OR "communities" OR "community-based" OR "community based" OR "home-based" OR "home based" OR "village") OR AB ("community" OR "communities" OR "community-based" OR "community based" OR "home-based" OR "home based" OR "village") OR SU ("community" OR "communities" OR "community-based" OR "community based" OR "home-based" OR "home based" OR "village")
**AND Community Health Worker terms**
TI ("community health workers" OR "community health worker" OR "community-based health workers" OR "community-based health worker" OR "community based health workers" OR "community based health worker" OR "community healthcare workers" OR "community healthcare worker" OR "community health care workers" OR "community health care worker" OR "community volunteers" OR "community volunteer" OR "community health volunteers" OR "community health volunteer" OR "volunteer health workers" OR

"volunteer health worker" OR "voluntary health workers" OR "voluntary health worker" OR "lay health workers" OR "lay health worker" OR "community relays" OR "community relay" OR "relais communautaires" OR "relais communautaire" OR "lady health workers" OR "lady health worker" OR "lady health visitors" OR "lady health visitor" OR "community based distributors" OR "community based distributor" OR "community-based distribution" OR "community based distribution" OR "community-based distributors" OR "community-based distributor" OR "community health distributors" OR "community health distributor" OR "community based agents" OR "community based agent" OR "community-based agents" OR "community-based agent" OR "health extension workers" OR "health extension worker" OR "community health agents" OR "community health agent" OR "agents de santé" OR "agent de santé" OR "agents de sante" OR "agent de sante" OR "health surveillance assistants" OR "health surveillance assistant" OR "community health assistants" OR "community health assistant" OR "community health surveyors" OR "community health surveyor" OR "community health promoters" OR "community health promoter" OR "rural health auxiliaries" OR "rural health auxiliary" OR "community owned resource persons" OR "community owned resource person" OR "community-owned resource persons" OR "community-owned resource person" OR "health aides" OR "health aide" OR "allied health personnel" OR "voluntary workers" OR "voluntary worker" OR "home nursing aides" OR "home nursing aide" OR "peer groups" OR "peer group" OR "doulas” OR "doula” OR "barefoot doctors" OR "barefoot doctor" OR "backpack health workers" OR "backpack health worker" OR "back pack health workers" OR "back pack health worker" OR "backpack health care workers" OR "backpack health care worker" OR "back pack health care workers" OR "back pack health care worker" OR "backpack healthcare workers" OR "backpack healthcare worker" OR "back pack healthcare workers" OR "back pack healthcare worker" OR "brigadistas" OR "brigadista" OR "community networks" OR "community network" OR "village polio volunteers" OR "village polio volunteer" OR "outreach volunteers" OR "outreach volunteer" OR "outreach workers" OR "outreach worker" OR "community networks" OR "community network" OR "community health nurses" OR "community health nurse" OR "community health officers" OR "community health officer" OR "village health workers" OR "village health worker" OR "village health volunteers" OR "village health volunteer" OR "malaria agents" OR "malaria agent" OR "community mobilizers" OR "community mobilizer" OR "community mobilisers" OR "community mobiliser" OR "polio volunteers" OR "polio volunteer" OR "volunteer health advisors" OR "volunteer health advisor" OR "village-based family planning workers" OR "village-based family planning worker" OR "village based family planning workers" OR "village based family planning worker" OR "community nutrition workers" OR "community nutrition worker" OR "community-based nutrition workers" OR "community-based nutrition worker" OR "community based nutrition workers" OR "community based nutrition worker" OR "community nutrition volunteers" OR "community nutrition volunteer" OR "community-based nutrition volunteers" OR "community-based nutrition volunteer" OR "community based nutrition volunteers" OR "community based nutrition volunteer" OR "accredited social health activists" OR "accredited social health activist" OR "anganwadi workers" OR "anganwadi worker" OR "agentes comunitários de saúde" OR "agente comunitário de saúde" OR "agentes comunitarios de saude" OR "agente comunitario de saude" OR "agentes polivalentes elementares" OR "agente polivalente elementar" OR "agentes comunitários de salud" OR "agente comunitário de salud" OR "agentes comunitarios de salud" OR "agente comunitarios de salud" OR "activistas" OR "activista" OR "barangay health workers" OR "barangay health worker" OR "colaboradores voluntários" OR "colaborador voluntário" OR "community drug distributors" OR "community drug distributor" OR "community health representatives" OR "community health representative" OR "female multipurpose health workers" OR "female multipurpose health worker" OR "female multi-purpose health workers" OR "female multi-purpose health worker" OR "kaders" OR "kader" OR "promotoras" OR "promotora" OR "rural health motivators" OR "rural health motivator" OR "village drug-kit managers" OR "village drug-kit manager" OR "village drug kit managers" OR "village drug kit manager" OR "village health helpers" OR "village health helper" OR "traditional birth attendants" OR "traditional birth attendant" OR "midwives" OR "midwife" OR "midwifery" OR "lay maternal health workers" OR "lay maternal health worker") OR AB ("community health workers" OR "community health worker" OR "community-based health workers" OR "community-based health worker" OR "community based health workers" OR "community based health worker" OR "community healthcare workers" OR "community healthcare worker" OR "community health care workers" OR "community health care worker" OR "community volunteers" OR "community volunteer" OR "community health volunteers" OR "community health volunteer" OR "volunteer health workers" OR "volunteer health worker" OR "voluntary health workers" OR "voluntary health worker" OR "lay health workers" OR "lay health worker" OR "community relays" OR "community relay" OR "relais communautaires" OR "relais communautaire" OR "lady health workers" OR "lady health worker" OR "lady health visitors" OR "lady health visitor" OR "community based distributors" OR "community based distributor" OR "community-based distribution" OR "community based distribution" OR "community-based distributors" OR "community-based distributor" OR "community health distributors" OR "community health distributor" OR "community based agents" OR "community based agent" OR "community-based agents" OR "community-based agent" OR "health extension workers" OR "health extension worker" OR "community health agents" OR "community health agent" OR "agents de santé" OR "agent de santé" OR "agents de sante" OR "agent de sante" OR "health surveillance assistants" OR "health surveillance assistant" OR "community health assistants" OR "community health assistant" OR "community health surveyors" OR "community health surveyor" OR "community health promoters" OR "community health promoter" OR "rural health auxiliaries" OR "rural health auxiliary" OR "community owned resource persons" OR "community owned resource person" OR "community-owned resource persons" OR "community-owned resource person"

OR "health aides" OR "health aide" OR "allied health personnel" OR "voluntary workers" OR "voluntary worker" OR "home nursing aides" OR "home nursing aide" OR "peer groups" OR "peer group" OR "doulas" OR "doula" OR "barefoot doctors" OR "barefoot doctor" OR "backpack health workers" OR "backpack health worker" OR "back pack health workers" OR "back pack health worker" OR "backpack health care workers" OR "backpack health care worker" OR "back pack health care workers" OR "back pack health care worker" OR "backpack healthcare workers" OR "backpack healthcare worker" OR "back pack healthcare workers" OR "back pack healthcare worker" OR "brigadistas" OR "brigadista" OR "community networks" OR "community network" OR "village polio volunteers" OR "village polio volunteer" OR "outreach volunteers" OR "outreach volunteer" OR "outreach workers" OR "outreach worker" OR "community networks" OR "community network" OR "community health nurses" OR "community health nurse" OR "community health officers" OR "community health officer" OR "village health workers" OR "village health worker" OR "village health volunteers" OR "village health volunteer" OR "malaria agents" OR "malaria agent" OR "community mobilizers" OR "community mobilizer" OR "community mobilisers" OR "community mobiliser" OR "polio volunteers" OR "polio volunteer" OR "volunteer health advisors" OR "volunteer health advisor" OR "village-based family planning workers" OR "village-based family planning worker" OR "village based family planning workers" OR "village based family planning worker" OR "community nutrition workers" OR "community nutrition worker" OR "community-based nutrition workers" OR "community-based nutrition worker" OR "community based nutrition workers" OR "community based nutrition worker" OR "community nutrition volunteers" OR "community nutrition volunteer" OR "community-based nutrition volunteers" OR "community-based nutrition volunteer" OR "community based nutrition volunteers" OR "community based nutrition volunteer" OR "accredited social health activists" OR "accredited social health activist" OR "anganwadi workers" OR "anganwadi worker" OR "agentes comunitários de saúde" OR "agente comunitário de saúde" OR "agentes comunitarios de saude" OR "agente comunitario de saude" OR "agentes polivalentes elementares" OR "agente polivalente elementar" OR "agentes comunitários de salud" OR "agente comunitário de salud" OR "agentes comunitarios de salud" OR "agente comunitarios de salud" OR "activistas" OR "activista" OR "barangay health workers" OR "barangay health worker" OR "colaboradores voluntários" OR "colaborador voluntário" OR "community drug distributors" OR "community drug distributor" OR "community health representatives" OR "community health representative" OR "female multipurpose health workers" OR "female multipurpose health worker" OR "female multi-purpose health workers" OR "female multi-purpose health worker" OR "kaders" OR "kader" OR "promotoras" OR "promotora" OR "rural health motivators" OR "rural health motivator" OR "village drug-kit managers" OR "village drug-kit manager" OR "village drug kit managers" OR "village drug kit manager" OR "village health helpers" OR "village health helper" OR "traditional birth attendants" OR "traditional birth attendant" OR "midwives" OR "midwife" OR "midwifery" OR "lay maternal health workers" OR "lay maternal health worker") OR SU ("community health workers" OR "community health worker" OR "community-based health workers" OR "community-based health worker" OR "community based health workers" OR "community based health worker" OR "community healthcare workers" OR "community healthcare worker" OR "community health care workers" OR "community health care worker" OR "community volunteers" OR "community volunteer" OR "community health volunteers" OR "community health volunteer" OR "volunteer health workers" OR "volunteer health worker" OR "voluntary health workers" OR "voluntary health worker" OR "lay health workers" OR "lay health worker" OR "community relays" OR "community relay" OR "relais communautaires" OR "relais communautaire" OR "lady health workers" OR "lady health worker" OR "lady health visitors" OR "lady health visitor" OR "community based distributors" OR "community based distributor" OR "community-based distribution" OR "community based distribution" OR "community-based distributors" OR "community-based distributor" OR "community health distributors" OR "community health distributor" OR "community based agents" OR "community based agent" OR "community-based agents" OR "community-based agent" OR "health extension workers" OR "health extension worker" OR "community health agents" OR "community health agent" OR "agents de santé" OR "agent de santé" OR "agents de sante" OR "agent de sante" OR "health surveillance assistants" OR "health surveillance assistant" OR "community health assistants" OR "community health assistant" OR "community health surveyors" OR "community health surveyor" OR "community health promoters" OR "community health promoter" OR "rural health auxiliaries" OR "rural health auxiliary" OR "community owned resource persons" OR "community owned resource person" OR "community-owned resource persons" OR "community-owned resource person" OR "health aides" OR "health aide" OR "allied health personnel" OR "voluntary workers" OR "voluntary worker" OR "home nursing aides" OR "home nursing aide" OR "peer groups" OR "peer group" OR "doulas" OR "doula" OR "barefoot doctors" OR "barefoot doctor" OR "backpack health workers" OR "backpack health worker" OR "back pack health workers" OR "back pack health worker" OR "backpack health care workers" OR "backpack health care worker" OR "back pack health care workers" OR "back pack health care worker" OR "backpack healthcare workers" OR "backpack healthcare worker" OR "back pack healthcare workers" OR "back pack healthcare worker" OR "brigadistas" OR "brigadista" OR "community networks" OR "community network" OR "village polio volunteers" OR "village polio volunteer" OR "outreach volunteers" OR "outreach volunteer" OR "outreach workers" OR "outreach worker" OR "community networks" OR "community network" OR "community health nurses" OR "community health nurse" OR "community health officers" OR "community health officer" OR "village health workers" OR "village health worker" OR "village health volunteers" OR "village health volunteer" OR "malaria agents" OR "malaria agent" OR "community mobilizers" OR "community mobilizer" OR "community mobilisers" OR "community mobiliser" OR "polio volunteers" OR "polio volunteer" OR "volunteer health advisors" OR

"volunteer health advisor" OR "village-based family planning workers" OR "village-based family planning worker" OR "village based family planning workers" OR "village based family planning worker" OR "community nutrition workers" OR "community nutrition worker" OR "community-based nutrition workers" OR "community-based nutrition worker" OR "community based nutrition workers" OR "community based nutrition worker" OR "community nutrition volunteers" OR "community nutrition volunteer" OR "community-based nutrition volunteers" OR "community-based nutrition volunteer" OR "community based nutrition volunteers" OR "community based nutrition volunteer" OR "accredited social health activists" OR "accredited social health activist" OR "anganwadi workers" OR "anganwadi worker" OR "agentes comunitários de saúde" OR "agente comunitário de saúde" OR "agentes comunitarios de saude" OR "agente comunitario de saude" OR "agentes polivalentes elementares" OR "agente polivalente elementar" OR "agentes comunitários de salud" OR "agente comunitário de salud" OR "agentes comunitarios de salud" OR "agente comunitarios de salud" OR "activistas" OR "activista" OR "barangay health workers" OR "barangay health worker" OR "colaboradores voluntários" OR "colaborador voluntário" OR "community drug distributors" OR "community drug distributor" OR "community health representatives" OR "community health representative" OR "female multipurpose health workers" OR "female multipurpose health worker" OR "female multi-purpose health workers" OR "female multi-purpose health worker" OR "kaders" OR "kader" OR "promotoras" OR "promotora" OR "rural health motivators" OR "rural health motivator" OR "village drug-kit managers" OR "village drug-kit manager" OR "village drug kit managers" OR "village drug kit manager" OR "village health helpers" OR "village health helper" OR "traditional birth attendants" OR "traditional birth attendant" OR "midwives" OR "midwife" OR "midwifery" OR "lay maternal health workers" OR "lay maternal health worker")
**AND Humanitarian Settings terms**
TI ("emergency” OR "emergencies” OR "humanitarian” OR "disaster” OR "disasters” OR "outbreak” OR "outbreaks” OR "epidemic” OR "epidemics” OR "crisis" OR "crises” OR "insecurity” OR "war” OR "wars” OR "war-torn” OR "warfare” OR "conflict" OR "conflicts" OR "tsunami" OR "tsunamis" OR "flooding” OR "flood” OR "floods” OR "cyclone” OR "cyclones” OR "hurricane” OR "hurricanes” OR "earthquake” OR "earthquakes” OR "drought” OR "droughts” OR "famine” OR "famines” OR "refugee” OR "refugees” OR "displaced” OR "displacement" OR “armed conflict” "emergency” OR "emergencies” OR "humanitarian” OR "disaster” OR "disasters” OR "outbreak” OR "outbreaks” OR "epidemic” OR "epidemics” OR "crisis" OR "crises” OR "insecurity” OR "war” OR "wars” OR "war-torn” OR "warfare” OR "conflict" OR "conflicts" OR "tsunami" OR "tsunamis" OR "flooding” OR "flood” OR "floods” OR "cyclone” OR "cyclones” OR "hurricane” OR "hurricanes” OR "earthquake” OR "earthquakes” OR "drought” OR "droughts” OR "famine” OR "famines” OR "refugee” OR "refugees” OR "displaced” OR "displacement" OR “armed conflict”) OR AB ("emergency” OR "emergencies” OR "humanitarian” OR "disaster” OR "disasters” OR "outbreak” OR "outbreaks” OR "epidemic” OR "epidemics” OR "crisis" OR "crises” OR "insecurity” OR "war” OR "wars” OR "war-torn” OR "warfare” OR "conflict" OR "conflicts" OR "tsunami" OR "tsunamis" OR "flooding” OR "flood” OR "floods” OR "cyclone” OR "cyclones” OR "hurricane” OR "hurricanes” OR "earthquake” OR "earthquakes” OR "drought” OR "droughts” OR "famine” OR "famines” OR "refugee” OR "refugees” OR "displaced” OR "displacement" OR “armed conflict” "emergency” OR "emergencies” OR "humanitarian” OR "disaster” OR "disasters” OR "outbreak” OR "outbreaks” OR "epidemic” OR "epidemics” OR "crisis" OR "crises” OR "insecurity” OR "war” OR "wars” OR "war-torn” OR "warfare” OR "conflict" OR "conflicts" OR "tsunami" OR "tsunamis" OR "flooding” OR "flood” OR "floods” OR "cyclone” OR "cyclones” OR "hurricane” OR "hurricanes” OR "earthquake” OR "earthquakes” OR "drought” OR "droughts” OR "famine” OR "famines” OR "refugee” OR "refugees” OR "displaced” OR "displacement" OR “armed conflict”) OR SU ("emergency” OR "emergencies” OR "humanitarian” OR "disaster” OR "disasters” OR "outbreak” OR "outbreaks” OR "epidemic” OR "epidemics” OR "crisis" OR "crises” OR "insecurity” OR "war” OR "wars” OR "war-torn” OR "warfare” OR "conflict" OR "conflicts" OR "tsunami" OR "tsunamis" OR "flooding” OR "flood” OR "floods” OR "cyclone” OR "cyclones” OR "hurricane” OR "hurricanes” OR "earthquake” OR "earthquakes” OR "drought” OR "droughts” OR "famine” OR "famines” OR "refugee” OR "refugees” OR "displaced” OR "displacement" OR “armed conflict” "emergency” OR "emergencies” OR "humanitarian” OR "disaster” OR "disasters” OR "outbreak” OR "outbreaks” OR "epidemic” OR "epidemics” OR "crisis" OR "crises” OR "insecurity” OR "war” OR "wars” OR "war-torn” OR "warfare” OR "conflict" OR "conflicts" OR "tsunami" OR "tsunamis" OR "flooding” OR "flood” OR "floods” OR "cyclone” OR "cyclones” OR "hurricane” OR "hurricanes” OR "earthquake” OR "earthquakes” OR "drought” OR "droughts” OR "famine” OR "famines” OR "refugee” OR "refugees” OR "displaced” OR "displacement" OR “armed conflict”)
**AND Low- and Middle-Income Country terms**
TI ("Afghanistan" OR "Albania" OR "Algeria" OR "American Samoa" OR "Angola" OR "Armenia" OR "Azerbaijan" OR "Bangladesh" OR "Belarus" OR "Byelarus" OR "Belorussia" OR "Belize" OR "Benin" OR "Bhutan" OR "Bolivia" OR "Bosnia" OR "Botswana" OR "Brazil" OR "Bulgaria" OR "Burma" OR "Burkina Faso" OR "Burundi" OR "Cabo Verde" OR "Cape Verde" OR "Cambodia" OR "Cameroon" OR "Central African Republic" OR "Chad" OR "China" OR "Colombia" OR "Comoros" OR "Comores" OR "Comoro" OR "Congo" OR "Costa Rica" OR "Côte d'Ivoire" OR "Cuba" OR "Djibouti" OR "Dominica" OR "Dominican Republic" OR "Ecuador" OR "Egypt" OR "El Salvador" OR "Eritrea" OR "Ethiopia" OR "Fiji" OR "Gabon" OR "Gambia" OR "Gaza" OR "Georgia" OR "Georgia Republic" OR "Ghana" OR "Grenada" OR "Grenadines" OR "Guatemala" OR "Guinea" OR "Guinea Bissau" OR "Guyana" OR "Haiti" OR "Herzegovina" OR "Hercegovina" OR "Honduras" OR "India" OR "Indonesia" OR "Iran" OR "Iraq" OR "Jamaica" OR "Jordan" OR "Kazakhstan" OR "Kenya" OR "Kiribati" OR "Korea" OR "Kosovo" OR "Kyrgyz" OR "Kirghizia" OR "Kirghiz" OR "Kyrgyzstan" OR "Lao PDR" OR "Laos" OR "Lebanon" OR "Lesotho" OR

"Liberia" OR "Libya" OR "Macedonia" OR "Madagascar" OR "Malawi" OR "Malay" OR "Malaya" OR "Malaysia" OR "Maldives" OR "Mali" OR "Marshall Islands" OR "Mauritania" OR "Mauritius" OR "Mexico" OR "Micronesia" OR "Moldova" OR "Mongolia" OR "Montenegro" OR "Morocco" OR "Mozambique" OR "Myanmar" OR "Namibia" OR "Nepal" OR "Nicaragua" OR "Niger" OR "Nigeria" OR "Pakistan" OR "Palau" OR "Panama" OR "Papua New Guinea" OR "Paraguay" OR "Peru" OR "Philippines" OR "Principe" OR "Romania" OR "Rwanda" OR "Ruanda" OR "Samoa" OR "Sao Tome" OR "Senegal" OR "Serbia" OR "Sierra Leone" OR "Solomon Islands" OR "Somalia" OR "South Africa" OR "South Sudan" OR "Sri Lanka" OR "St Lucia" OR "St Vincent" OR "Sudan" OR "Surinam" OR "Suriname" OR "Swaziland" OR "Syria" OR "Syrian Arab Republic" OR "Tajikistan" OR "Tadzhikistan" OR "Tajikistan" OR "Tadzhik" OR "Tanzania" OR "Thailand" OR "Timor" OR "Togo" OR "Tonga" OR "Tunisia" OR "Turkey" OR "Turkmen" OR "Turkmenistan" OR "Tuvalu" OR "Uganda" OR "Ukraine" OR "Uzbek" OR "Uzbekistan" OR "Vanuatu" OR "Vietnam" OR "West Bank" OR "Yemen" OR "Zambia" OR "Zimbabwe" OR "Deprived Countries" OR "Deprived Population" OR "Deprived Populations" OR "Developing Countries" OR "Developing Country" OR "Developing Economies" OR "Developing Economy" OR "Developing Nation" OR "Developing Nations" OR "Developing Population" OR "Developing Populations" OR "Developing World" OR "LAMI Countries" OR "LAMI Country" OR "Less Developed Countries" OR "Less Developed Country" OR "Less Developed Economies" OR "Less Developed Nation" OR "Less Developed Nations" OR "Less Developed World" OR "Lesser Developed Countries" OR "Lesser Developed Nations" OR "LMIC" OR "LMICS" OR "Low GDP" OR "Low GNP" OR "Low Gross Domestic" OR "Low Gross National" OR "Low Income Countries" OR "Low Income Country" OR "Low Income Economies" OR "Low Income Economy" OR "Low Income Nations" OR "Low Income Population" OR "Low Income Populations" OR "Lower GDP" OR "lower gross domestic" OR "Lower Income Countries" OR "Lower Income Country" OR "Lower Income Nations" OR "Lower Income Population" OR "Lower Income Populations" OR "Middle Income Countries" OR "Middle Income Country" OR "Middle Income Economies" OR "Middle Income Nation" OR "Middle Income Nations" OR "Middle Income Population" OR "Middle Income Populations" OR "Poor Countries" OR "Poor Country" OR "Poor Economies" OR "Poor Economy" OR "Poor Nation" OR "Poor Nations" OR "Poor Population" OR "Poor Populations" OR "poor world" OR "Poorer Countries" OR "Poorer Economies" OR "Poorer Economy" OR "Poorer Nations" OR "Poorer Population" OR "Poorer Populations" OR "Third World" OR "Transitional Countries" OR "Transitional Country" OR "Transitional Economies" OR "Transitional Economy" OR "Under Developed Countries" OR "Under Developed Country" OR "under developed nations" OR "Under Developed World" OR "Under Served Population" OR "Under Served Populations" OR "Underdeveloped Countries" OR "Underdeveloped Country" OR "underdeveloped economies" OR "underdeveloped nations" OR "underdeveloped population" OR "Underdeveloped World" OR "Underserved Countries" OR "Underserved Nations" OR "Underserved Population" OR "Underserved Populations") OR AB ("Afghanistan" OR "Albania" OR "Algeria" OR "American Samoa" OR "Angola" OR "Armenia" OR "Azerbaijan" OR "Bangladesh" OR "Belarus" OR "Byelarus" OR "Belorussia" OR "Belize" OR "Benin" OR "Bhutan" OR "Bolivia" OR "Bosnia" OR "Botswana" OR "Brazil" OR "Bulgaria" OR "Burma" OR "Burkina Faso" OR "Burundi" OR "Cabo Verde" OR "Cape Verde" OR "Cambodia" OR "Cameroon" OR "Central African Republic" OR "Chad" OR "China" OR "Colombia" OR "Comoros" OR "Comores" OR "Comoro" OR "Congo" OR "Costa Rica" OR "Côte d'Ivoire" OR "Cuba" OR "Djibouti" OR "Dominica" OR "Dominican Republic" OR "Ecuador" OR "Egypt" OR "El Salvador" OR "Eritrea" OR "Ethiopia" OR "Fiji" OR "Gabon" OR "Gambia" OR "Gaza" OR "Georgia" OR "Georgia Republic" OR "Ghana" OR "Grenada" OR "Grenadines" OR "Guatemala" OR "Guinea" OR "Guinea Bissau" OR "Guyana" OR "Haiti" OR "Herzegovina" OR "Hercegovina" OR "Honduras" OR "India" OR "Indonesia" OR "Iran" OR "Iraq" OR "Jamaica" OR "Jordan" OR "Kazakhstan" OR "Kenya" OR "Kiribati" OR "Korea" OR "Kosovo" OR "Kyrgyz" OR "Kirghizia" OR "Kirghiz" OR "Kyrgyzstan" OR "Lao PDR" OR "Laos" OR "Lebanon" OR "Lesotho" OR "Liberia" OR "Libya" OR "Macedonia" OR "Madagascar" OR "Malawi" OR "Malay" OR "Malaya" OR "Malaysia" OR "Maldives" OR "Mali" OR "Marshall Islands" OR "Mauritania" OR "Mauritius" OR "Mexico" OR "Micronesia" OR "Moldova" OR "Mongolia" OR "Montenegro" OR "Morocco" OR "Mozambique" OR "Myanmar" OR "Namibia" OR "Nepal" OR "Nicaragua" OR "Niger" OR "Nigeria" OR "Pakistan" OR "Palau" OR "Panama" OR "Papua New Guinea" OR "Paraguay" OR "Peru" OR "Philippines" OR "Principe" OR "Romania" OR "Rwanda" OR "Ruanda" OR "Samoa" OR "Sao Tome" OR "Senegal" OR "Serbia" OR "Sierra Leone" OR "Solomon Islands" OR "Somalia" OR "South Africa" OR "South Sudan" OR "Sri Lanka" OR "St Lucia" OR "St Vincent" OR "Sudan" OR "Surinam" OR "Suriname" OR "Swaziland" OR "Syria" OR "Syrian Arab Republic" OR "Tajikistan" OR "Tadzhikistan" OR "Tajikistan" OR "Tadzhik" OR "Tanzania" OR "Thailand" OR "Timor" OR "Togo" OR "Tonga" OR "Tunisia" OR "Turkey" OR "Turkmen" OR "Turkmenistan" OR "Tuvalu" OR "Uganda" OR "Ukraine" OR "Uzbek" OR "Uzbekistan" OR "Vanuatu" OR "Vietnam" OR "West Bank" OR "Yemen" OR "Zambia" OR "Zimbabwe" OR "Deprived Countries" OR "Deprived Population" OR "Deprived Populations" OR "Developing Countries" OR "Developing Country" OR "Developing Economies" OR "Developing Economy" OR "Developing Nation" OR "Developing Nations" OR "Developing Population" OR "Developing Populations" OR "Developing World" OR "LAMI Countries" OR "LAMI Country" OR "Less Developed Countries" OR "Less Developed Country" OR "Less Developed Economies" OR "Less Developed Nation" OR "Less Developed Nations" OR "Less Developed World" OR "Lesser Developed Countries" OR "Lesser Developed Nations" OR "LMIC" OR "LMICS" OR "Low GDP" OR "Low GNP" OR "Low Gross Domestic" OR "Low Gross National" OR "Low Income Countries" OR "Low Income Country" OR "Low Income Economies" OR "Low Income Economy" OR "Low Income Nations" OR "Low Income Population" OR "Low Income Populations" OR "Lower GDP" OR "lower gross domestic" OR "Lower Income Countries" OR "Lower Income Country" OR "Lower Income Nations" OR "Lower Income Population" OR "Lower Income Populations" OR "Middle Income Countries" OR "Middle Income

Country" OR "Middle Income Economies" OR "Middle Income Nation" OR "Middle Income Nations" OR "Middle Income Population" OR "Middle Income Populations" OR "Poor Countries" OR "Poor Country" OR "Poor Economies" OR "Poor Economy" OR "Poor Nation" OR "Poor Nations" OR "Poor Population" OR "Poor Populations" OR "poor world" OR "Poorer Countries" OR "Poorer Economies" OR "Poorer Economy" OR "Poorer Nations" OR "Poorer Population" OR "Poorer Populations" OR "Third World" OR "Transitional Countries" OR "Transitional Country" OR "Transitional Economies" OR "Transitional Economy" OR "Under Developed Countries" OR "Under Developed Country" OR "under developed nations" OR "Under Developed World" OR "Under Served Population" OR "Under Served Populations" OR "Underdeveloped Countries" OR "Underdeveloped Country" OR "underdeveloped economies" OR "underdeveloped nations" OR "underdeveloped population" OR "Underdeveloped World" OR "Underserved Countries" OR "Underserved Nations" OR "Underserved Population" OR "Underserved Populations") OR SU ("Afghanistan" OR "Albania" OR "Algeria" OR "American Samoa" OR "Angola" OR "Armenia" OR "Azerbaijan" OR "Bangladesh" OR "Belarus" OR "Byelarus" OR "Belorussia" OR "Belize" OR "Benin" OR "Bhutan" OR "Bolivia" OR "Bosnia" OR "Botswana" OR "Brazil" OR "Bulgaria" OR "Burma" OR "Burkina Faso" OR "Burundi" OR "Cabo Verde" OR "Cape Verde" OR "Cambodia" OR "Cameroon" OR "Central African Republic" OR "Chad" OR "China" OR "Colombia" OR "Comoros" OR "Comores" OR "Comoro" OR "Congo" OR "Costa Rica" OR "Côte d'Ivoire" OR "Cuba" OR "Djibouti" OR "Dominica" OR "Dominican Republic" OR "Ecuador" OR "Egypt" OR "El Salvador" OR "Eritrea" OR "Ethiopia" OR "Fiji" OR "Gabon" OR "Gambia" OR "Gaza" OR "Georgia" OR "Georgia Republic" OR "Ghana" OR "Grenada" OR "Grenadines" OR "Guatemala" OR "Guinea" OR "Guinea Bissau" OR "Guyana" OR "Haiti" OR "Herzegovina" OR "Hercegovina" OR "Honduras" OR "India" OR "Indonesia" OR "Iran" OR "Iraq" OR "Jamaica" OR "Jordan" OR "Kazakhstan" OR "Kenya" OR "Kiribati" OR "Korea" OR "Kosovo" OR "Kyrgyz" OR "Kirghizia" OR "Kirghiz" OR "Kyrgyzstan" OR "Lao PDR" OR "Laos" OR "Lebanon" OR "Lesotho" OR "Liberia" OR "Libya" OR "Macedonia" OR "Madagascar" OR "Malawi" OR "Malay" OR "Malaya" OR "Malaysia" OR "Maldives" OR "Mali" OR "Marshall Islands" OR "Mauritania" OR "Mauritius" OR "Mexico" OR "Micronesia" OR "Moldova" OR "Mongolia" OR "Montenegro" OR "Morocco" OR "Mozambique" OR "Myanmar" OR "Namibia" OR "Nepal" OR "Nicaragua" OR "Niger" OR "Nigeria" OR "Pakistan" OR "Palau" OR "Panama" OR "Papua New Guinea" OR "Paraguay" OR "Peru" OR "Philippines" OR "Principe" OR "Romania" OR "Rwanda" OR "Ruanda" OR "Samoa" OR "Sao Tome" OR "Senegal" OR "Serbia" OR "Sierra Leone" OR "Solomon Islands" OR "Somalia" OR "South Africa" OR "South Sudan" OR "Sri Lanka" OR "St Lucia" OR "St Vincent" OR "Sudan" OR "Surinam" OR "Suriname" OR "Swaziland" OR "Syria" OR "Syrian Arab Republic" OR "Tajikistan" OR "Tadzhikistan" OR "Tajikistan" OR "Tadzhik" OR "Tanzania" OR "Thailand" OR "Timor" OR "Togo" OR "Tonga" OR "Tunisia" OR "Turkey" OR "Turkmen" OR "Turkmenistan" OR "Tuvalu" OR "Uganda" OR "Ukraine" OR "Uzbek" OR "Uzbekistan" OR "Vanuatu" OR "Vietnam" OR "West Bank" OR "Yemen" OR "Zambia" OR "Zimbabwe" OR "Deprived Countries" OR "Deprived Population" OR "Deprived Populations" OR "Developing Countries" OR "Developing Country" OR "Developing Economies" OR "Developing Economy" OR "Developing Nation" OR "Developing Nations" OR "Developing Population" OR "Developing Populations" OR "Developing World" OR "LAMI Countries" OR "LAMI Country" OR "Less Developed Countries" OR "Less Developed Country" OR "Less Developed Economies" OR "Less Developed Nation" OR "Less Developed Nations" OR "Less Developed World" OR "Lesser Developed Countries" OR "Lesser Developed Nations" OR "LMIC" OR "LMICS" OR "Low GDP" OR "Low GNP" OR "Low Gross Domestic" OR "Low Gross National" OR "Low Income Countries" OR "Low Income Country" OR "Low Income Economies" OR "Low Income Economy" OR "Low Income Nations" OR "Low Income Population" OR "Low Income Populations" OR "Lower GDP" OR "lower gross domestic" OR "Lower Income Countries" OR "Lower Income Country" OR "Lower Income Nations" OR "Lower Income Population" OR "Lower Income Populations" OR "Middle Income Countries" OR "Middle Income Country" OR "Middle Income Economies" OR "Middle Income Nation" OR "Middle Income Nations" OR "Middle Income Population" OR "Middle Income Populations" OR "Poor Countries" OR "Poor Country" OR "Poor Economies" OR "Poor Economy" OR "Poor Nation" OR "Poor Nations" OR "Poor Population" OR "Poor Populations" OR "poor world" OR "Poorer Countries" OR "Poorer Economies" OR "Poorer Economy" OR "Poorer Nations" OR "Poorer Population" OR "Poorer Populations" OR "Third World" OR "Transitional Countries" OR "Transitional Country" OR "Transitional Economies" OR "Transitional Economy" OR "Under Developed Countries" OR "Under Developed Country" OR "under developed nations" OR "Under Developed World" OR "Under Served Population" OR "Under Served Populations" OR "Underdeveloped Countries" OR "Underdeveloped Country" OR "underdeveloped economies" OR "underdeveloped nations" OR "underdeveloped population" OR "Underdeveloped World" OR "Underserved Countries" OR "Underserved Nations" OR "Underserved Population" OR "Underserved Populations")

Appendix S2: Key information from included documents

Lead author	Title	Year	Publishing Organization	Type of document	Journal/ conference	World Health Organization region	Country	Type of crisis	Crisis setting	Thematic areas	Interventions provided by CHWs	CHW cadre	Study methods	Key findings
Adam	Relationship between implementing interpersonal communication and mass education campaigns in emergency settings and use of reproductive healthcare services: evidence from Darfur, Sudan	2015	Tokyo Medical and Dental University	Journal article	BMJ Open	Eastern Mediterranean	Sudan	Conflict	Camp	Reproductive health	Home visits and mass education activities	Maternal community health workers	Pre-post surveys, no comparison	− Health communication and health services not appropriate for displaced persons who may be from different ethnic groups, have different culture.<br>− CHWs used in IDP camp setting to do behavior change interventions for maternal health.<br>− Awareness and use of maternal health services over time.<br>− Significant associations between receiving behavior change interventions and use of maternal health services.
Adam	The influence of maternal health education on the place of delivery in conflict settings of Darfur, Sudan	2015	Tokyo Medical and Dental University, University of Khartoum	Journal article	Conflict and Health	Eastern Mediterranean	Sudan	Conflict	Camp	Maternal health, reproductive health	Health education	Maternal health workers	Household survey	− Female community members were trained to provide maternal health education and to promote facility delivery among IDP women. Receiving a home visit for maternal health education was statistically significant with place of delivery.
Ager	Health service resilience in Yobe state, Nigeria in the context of the Boko Haram insurgency: a systems dynamics analysis using group model building	2015	Columbia University, Queen Margaret University, University of the Western Cape, Partnership for Reviving Routine Immunization in Northern Nigeria and Maternal Newborn Child Health, University of Montreal	Journal article	Conflict and Health	Africa	Nigeria	Conflict	General population	Not specified	Not specified	Community health workers	Qualitative interviews, group model building	− CHWs coordinated with community members to warn health workers when it was not safe to travel to communities.
Ahmadzai	Scaling up TB DOTS in a fragile state: post-conflict Afghanistan	2008	Afghanistan Ministry of Public Health, Management Sciences for Health	Journal article	International Journal of Tuberculosis and Lung Disease	Eastern Mediterranean	Afghanistan	Conflict	General population	Infectious diseases	Diagnosis and treatment of tuberculosis	Community health workers	Analysis of quantitative program data	− CHWs were trained to diagnose and treat TB (with DOTS) in communities. CHWs were see as an important part of the effort that increased the number of districts implementing DOTS by 600% over four years, increased the number of patients treated for TB by 380% over two years, and increased the cure rate from 59% to 87% over 3 years.
Ahmed	Community midwives' acceptability in their communities: A qualitative study from two provinces of Pakistan	2017	Arabian Gulf University, United Nations Children's Fund, Human Development Programme	Journal article	Midwifery	Eastern Mediterranean	Pakistan	Conflict	General population	Maternal health, newborn health	Assisted home delivery, newborn care	Community midwives	Qualitative interviews, focus group discussions	− Insecurity contributed to difficulties in traveling for community midwives.

Akseer	Achieving maternal and child health gains in Afghanistan: a Countdown to 2015 country case study	2016	Hospital for Sick Children, London School of Hygiene & Tropical Medicine, United Nations Children's Fund, Afghanistan Ministry of Public Health, Aga Khan University, University of Toronto	Journal article	Lancet Global Health	Eastern Mediterranean	Afghanistan	Conflict	General population	Maternal health, newborn health, child health, reproductive health	Not specified	Community health workers	Document review, analysis of quantitative data	− Afghanistan has only recently invested heavily in CHWs, so they had not played a role in RMNCH outcomes at the national level in 2016.
Allam	An Outbreak of Cholera due to Contaminated Water, Medak District, Andhra Pradesh, India, 2013	2015	SHARE India, Hyderabad Directorate of Health, India National Institute of Epidemiology	Commentary	Indian Journal of Community Medicine	South-East Asia	India	Disease outbreak	General population	Infectious diseases	Active case finding	Community health workers	Retrospective cohort study, household census	− CHWs carried out active case finding during a cholera outbreak.
Almedom	Maternal psychosocial well-being in Eritrea: application of participatory methods and tools of investigation and analysis in complex emergency settings	2003	Tufts University, Eritrea Ministry of Environment, Water and Land, Eritrea Ministry of Health, Eritrean Relief and Refugee Commission	Journal article	Bulletin of the World Health Organization	Africa	Eritrea	Conflict	Camp	Maternal health, mental health	Assisted home delivery, referral of delivering women to health facility	Traditional birth attendants	Group discussions	− TBAs were more trusted and were seen as giving more respectful care than health facility workers.<br>− Post-partum support provided by TBAs was seen as supportive of mothers' mental health.
Alonge	The effect of contracting for health services on the equity of utilization and out-of-pocket health expenditure in rural Afghanistan	2012	Johns Hopkins University	Dissertation	N/A	Eastern Mediterranean	Afghanistan	Conflict	General population	Not specified	Not specified	Community health workers	Analysis of household survey, health facility survey, and HMIS data	− Health facilities with more than 10 CHWs compared to those with none were significantly pro-poor.
Amthor	The Use of Home-Based Therapy with Ready-to-Use Therapeutic Food to Treat Malnutrition in a Rural Area during a Food Crisis	2009	Washington University	Journal article	Journal of the American Dietetic Association	Africa	Malawi	Nutrition emergency	General population	Nutrition	Diagnosis of child malnutrition and prescription of RUTF	Village health aides	Longitudinal tracking of malnutrition status of children	− Using CHWs to diagnose and prescribe RUTF for home-based treatment for severely malnourished children was feasible and resulted in high treatment and recovery rates.<br>− The program was rolled-out rapidly, with only 10 days for preparation and training of CHWs.<br>− Besides the initial training of village health aides, no medical personnel were needed to manage the intervention.
Anderson	Role of community health workers in trachoma control: Case study from a Somali refugee camp	1986	Institute of Ophthalmology, Save the Children	Journal article	Tropical Doctor	Eastern Mediterranean	Somalia	Conflict	Camp	Infectious diseases	Diagnosis and treatment of trachoma, health education	Community health workers	Pre-post household surveys, no comparison	− CHWs were trained to diagnose and treat trachoma and provide education on prevention of trachoma.<br>− Before and after surveys showed a statistically significant decrease in active and severe trachoma.

Anwari	Implementing people-centred health systems governance in 3 provinces and 11 districts of Afghanistan: a case study	2015	Management Sciences for Health, Wardak Provincial Public Health Office, Khost Provincial Public Health Office, Herat Provincial Public Health Office, Afghanistan Ministry of Public Health	Journal article	Conflict and Health	Eastern Mediterranean	Afghanistan	Conflict	General population	Not specified	Not specified	Community health workers	Pre-post surveys, analysis of quantitative program data, focus group discussions	N/A
Ardalan	Effectiveness of a Primary Health Care Program on Urban and Rural Community Disaster Preparedness, Islamic Republic of Iran: A Community Intervention Trial	2013	Iranian National Institute of Health Research, Tehran University of Medical Sciences, Golestan University of Medical Sciences, Kerman University of Medical Sciences, Harvard University, Yale University	Journal article	Disaster Medicine and Public Health Preparedness	Eastern Mediterranean	Iran	Natural disaster	General population	Disaster preparedness	Disaster preparedness education	Community health workers	Pre-post household surveys, with comparison	− CHWs were used to carry out a community education intervention for disaster awareness and preparedness.<br>− All measures of household awareness and readiness increased significantly in the intervention area compared to the control area over the 6-month study period.
Balaluka	Community volunteers can improve breastfeeding among children under six months of age in the Democratic Republic of Congo crisis	2012	Centre de Recherche en Sciences Naturelles de Lwiro, Université Libre de Bruxelles	Journal article	International Breastfeeding Journal	Africa	Democratic Republic of Congo	Conflict	General population	Newborn health, nutrition	Promotion of exclusive breastfeeding, growth monitoring	Community volunteers	Unclear	− Community volunteers were trained to promote exclusive breastfeeding in an area affected by chronic conflict and insecurity.<br>− The proportion of infants who were exclusively breastfed from birth at 4, 5, and 6 months of age was also significantly higher in the intervention area than in the comparison area.
Barbera	Phase IV progress report: "Medicine at our doorstep" January-December 2010	Not specified	International Rescue Committee	Report	N/A	Africa	Ivory Coast	Conflict	General population	Child health	Treatment of childhood pneumonia, diarrhea, and malaria; bednet distribution; vitamin A supplementation	Community based distributors	Analysis of quantitative program data	− Post-election violence and insecurity in Ivory Coast caused stockouts of iCCM drugs. In response, larger stocks were given to CHWs and extra incentives were given to supervisors.<br>− Geographic expansion to the second health district was disrupted by the military and political crisis linked to the presidential elections.
Bass	Controlled Trial of Psychotherapy for Congolese Survivors of Sexual Violence	2013	Johns Hopkins Bloomberg School of Public Health, International Rescue Committee, University of Washington, University of Texas	Journal article	New England Journal of Medicine	Africa	Democratic Republic of Congo	Conflict	General population	Mental health	Psychosocial support	Psychosocial assistants	Randomized controlled trial	− CHWs provided psychosocial support to women in areas with high levels of illiteracy and insecurity.<br>− Psychosocial health status of participants improved among those receiving both group and individual therapy, with the largest improvements among those in group therapy.
Becker	Psychosocial care for adult and child survivors of the tsunami disaster in India	2007	Georgetown University	Journal article	Journal of Child and Adolescent Psychiatric Nursing	South-East Asia	India	Natural disaster	General population, camp	Mental health	Psychosocial support	Community workers, teachers	Documentation of experience	− Teachers and other community members were trained to provide psychosocial support, through play therapy for children and group therapy for adults.

Bell	"Ebola kills generations": Qualitative discussions with Liberian healthcare providers	2017	University of Michigan School of Nursing, Africare	Journal article	Midwifery	Africa	Liberia	Disease outbreak	General population	Not specified	Not specified	General community health volunteers, traditional birth attendants	Focus group discussions	− During Ebola, CHWs were at increased risk of infection because of their contact with sick people in the community and the lack of safety equipment, such as PPE and non-contact thermometers.
Bhadra	Psychosocial Support for the Children Affected by Communal Violence in Gujarat, India	2012	Oxfam India	Journal article	International Journal of Applied Psychoanalytic Studies	South-East Asia	India	Conflict	General population, camp	Mental health	Psychosocial support	Community volunteers	Documentation of experience	− Community members were trained to provide psychosocial support to children and their caregivers in the aftermath of large-scale communal violence.
Bhatia	A social and demographic study of Tibetan refugees in India	2002	Tibetan Government in Exile, University of Adelaide	Journal article	Social Science and Medicine	South-East Asia	India	Conflict	Camp	Maternal health, newborn health, child health	Community-based surveillance, verbal autopsy	Community health workers	Documentation of experience	− CHWs carried out surveillance of births, morbidity, mortality, and coverage of health interventions among the refugee community.
Bhutta	Reproductive, maternal, newborn, and child health in Pakistan: challenges and opportunities	2013	Aga Khan University, Health Services Academy, John Snow, Save the Children, National Institute of Population Studies, Jinnah Postgraduate Medical Centre, Pakistan Institute of Medical Sciences, Zlauddin Medical University	Journal article	Lancet	Eastern Mediterranean	Pakistan	Conflict	General population	Child health, newborn health, maternal health, reproductive health	Health education, provision of contraceptives, nutritional assessment and education, promotion of immunization	Lady health workers	Retrospective case study	− LHW program is institutionalized and covers 60% of the rural population.<br>− Evaluations have found better health indicators in LHW covered areas.<br>− Key constraints include poor support from health facilities, financial constraints, and political interference.<br>− Polio workers have been targeted by armed actors.
Bisimwa	Nutritional monitoring of preschool-age children by community volunteers during armed conflict in the Democratic Republic of the Congo	2009	Centre de Recherche en Sciences Naturelles de Lwiro, Centre Scientifique et Médical del'Université Libre de Bruxelles	Journal article	Food and Nutrition Bulletin	Africa	Democratic Republic of Congo	Conflict	General population	Nutrition	Growth monitoring, nutrition education	Community volunteers	Documentation of experience, analysis of quantitative program data	− CHWs carried out growth monitoring and nutrition education in their communities in a context of conflict and insecurity.<br>− Nearly 95% of monthly growth monitoring reports were received over a 2-year period.
Bisimwa	The effectiveness of community volunteers in counting populations and assessing their nutritional vulnerability during armed conflict: District health in D.R. Congo, Central Africa	2009	Universite´ libre de Bruxelles, Centre de recherche en sciences naturelles de Lwiro	Journal article	Cahiers Santé	Africa	Democratic Republic of Congo	Conflict	General population	Nutrition	Community census, nutrition assessment	Community volunteers	Documentation of experience	− Community volunteers carried out a household census and nutrition assessment in their communities in a context of conflict and insecurity.
Boddam-Whetham	Vouchers in Fragile States: Reducing Barriers to Long-Acting Reversible Contraception in Yemen and Pakistan	2016	Options Consultancy Services, Marie Stopes Society Pakistan	Journal article	Global Health: Science and Practice	Eastern Mediterranean	Pakistan, Yemen	Conflict	General population	Reproductive health	Provision of contraceptives	Community midwives	Documentation of experience, analysis of quantitative program data	− Community midwives distributed vouchers for contraceptives.

Bowden	Implementation and utilisation of community-based mortality surveillance: a case study from Chad	2012	Imperial College London, Cochrane Medical Education Centre, Médecins Sans Frontières, London School of Hygiene and Tropical Medicine,	Journal article	Conflict and Health	Africa	Chad	Conflict	Camp	Surveillance	Mortality surveillance	Community health workers	Qualitative interviews, document review	− CHWs carried out mortality surveillance in IDP camps.<br>− Using local CHWs may have helped to overcome some of the social, political, economic and cultural barriers to data collection on deaths.<br>− Implementation of the community-based mortality surveillance took several months to initiate, indicating that implementation of community-based surveillance may be too slow to be useful in an acute emergency.
Boyd	TB control in humanitarian emergencies: Lessons from the Syria displacement crisis	2019	U.S. Centers for Disease Control and Prevention	Journal article	Journal of Clinical Tuberculosis and Other Mycobacterial Diseases	Eastern Mediterranean, Europe	Iraq, Jordan, Lebanon, Syria, Turkey	Conflict	General population, camp	Infectious diseases	TB screening and detection, provision of DOTS, monitoring treatment adherence, community awareness	Community health workers	Documentation of experience	− CHWs carried out screening and detection of presumptive TB cases, provision of DOTS, monitored treatment adherence, and community awareness on TB.
Brentlinger	Pregnancy outcomes in El Salvador during the post-war period	1997	St. Anne’s Hospital, Columbia University College of Physicians and Surgeons	Journal article	International Journal of Gynecology and Obstetrics	Americas	El Salvador	Conflict	General population	Maternal health, newborn health	Assisted home delivery	Trained lay midwives	Household surveys	− Lay midwives were trained to facilitate home deliveries during civil war.<br>− The project achieved high rates of assisted delivery and the project area had very low rates of delivery complications.
Brentlinger	Health sector response to security threats during the civil war in El Salvador	1996	St. Anne's Hospital	Journal article	British Medical Journal	Americas	El Salvador	Conflict	General population	Child health, infectious diseases, WASH, first aid	Treatment of pneumonia, diarrhea, malaria, and malnutrition; first aid; WASH promotion	Health promoters	Documentation of experience	− There was widespread targeting of health workers, including CHWs during the war in El Salvador.<br>− CHWs were trained to overcome a lack of access to health services through health facilities.<br>− CHWs took several measures to maintain a low profile to reduce the risk of attacks or confiscation of commodities.
Bristol	Reconstructing Afghanistan’s health system	2005	N/A	Commentary	The Lancet	Eastern Mediterranean	Afghanistan	Conflict	General population	Maternal health, newborn health, child health	Immunization, health education	Community health workers	Documentation of experience	− CHWs were trained to provide basic healthcare services in rural areas.
Britten	Health care in Nicaragua	1989	Health Network of the Nicaragua Solidarity Campaign	Journal article	Medicine and War	Americas	Nicaragua	Conflict	General population	Newborn health, child health	Immunization, treatment of diarrhea, health education	Brigadistas	Documentation of experience	− CHWs carried out childhood immunization in communities, which led to high rates of immunization.<br>− Health workers, including CHWs were targeted for killing by militias.
Broholm	Health Effects of the War in Two Rural Communities in Nicaragua	1989	Nicaragua Health Study Collaborative	Journal article	American Journal of Public Health	Americas	Nicaragua	Conflict	General population	Not specified	Not specified	Brigadistas	Survey with comparison, qualitative interviews, focus group discussions, document review	− In one area with 20 brigadistas, 10 had been kidnapped and 5 had quit because of threats of violence.

Budosan	A mixed methods field based assessment to design a mental health intervention after the 2005 earthquake in Mansehra, North-West Frontier Province, Pakistan	2009	International Medical Corps,	Journal article	Intervention	Eastern Mediterranean	Pakistan	Natural disaster	Camp	Mental health	Mental health education	Community volunteers	Household survey, qualitative interviews, focus group discussions, participant observation, health worker knowledge test	− Community volunteers were trained to provide community education on mental health issues following an earthquake.
Budosan	Strategy for providing integrated mental health/psychosocial support in post earthquake Haiti	2011	Cordaid	Journal article	Intervention	Americas	Haiti	Natural disaster	General population, camp	Mental health	Not specified	Community health workers	Household survey, qualitative interviews, focus group discussions, health worker knowledge test	− CHWs were trained to provide psychosocial support following the Haiti earthquake.
Callister	The Mobile Obstetric Maternal (MOM) Health Workers Project: Improving Maternal and Newborn Health in Eastern Burma	2012	Brigham Young University	Commentary	Global Health and Nursing	South-East Asia	Myanmar	Conflict	General population	Maternal health, newborn health	Not specified	Not specified, traditional birth attendants	Documentation of experience	− Community-based health workers were part of a program to improve maternal and newborn health in conflict-affected areas.<br>− The number of women receiving prenatal care increased from 39% before the project to 72%, and provider attendance at birth increased from 5% to 49%.
Campbell	Promoting community health in Sudan	1999	GOAL	Journal article	Primary Health Care	Eastern Mediterranean	Sudan	Conflict	Camp	Nutrition, infectious diseases	Health education, malnutrition screening and referral, follow-up of clinic defaulters, disease surveillance	Community health promoters	Documentation of experience	− Community volunteers carried out health education, nutrition screening and referral, and disease surveillance in IDP camps.<br>− Some volunteers went on strike to protest the lack of salary.
Campbell	Responding to Cyclone Nargis: Key lessons from Merlin's experience	2008	Merlin	Journal article	Humanitarian Practice Network	South-East Asia	Myanmar	Natural disaster	General population	Maternal health, child health	Treatment of childhood pneumonia, diarrhea, and malaria; health education; first aid	Community health workers	Documentation of experience	− After the cyclone hit, CHWs were providing care in their communities before any mobile teams from NGOs were able to access the areas.<br>− CHWs were a crucial part of the emergency relief effort at the community level. They helped carry out needs assessments and distribute aid based on their knowledge of the community.

Cancedda	Strengthening Health Systems While Responding to a Health Crisis: Lessons Learned by a Nongovernmental Organization During the Ebola Virus Disease Epidemic in Sierra Leone	2016	Partners In Health, Brigham and Women's Hospital, Harvard Medical School, Boston Children's Hospital, Boston University School of Medicine, University of California–San Francisco School of Medicine, Stanford University School of Medicine, Stanford University, Wellbody Alliance, Koidu Government Hospital, Koidu District Health Management Team, Princess Christian Maternity Hospital, Sierra Leone Ministry of Health and Sanitation, Port Loko Government Hospital, University of Washington	Journal article	Journal of Infectious Diseases	Africa	Sierra Leone	Disease outbreak	General population	Infectious diseases	Infectious disease control, psychosocial support	Community health workers	Documentation of experience	− CHWs conducted over 1.1 million home visits in Ebola-affected communities to identify individuals with symptoms of EVD, referred 357 of those individuals to health facilities, monitored hundreds of family members of patients with Ebola who were under quarantine at home, and delivered educational messages on Ebola to nearly 900,000 Sierra Leoneans.<br>− CHWs were trained to provide psychosocial support to Ebola-affected households and individuals.<br>− CHWs were able to leverage local knowledge, trust, and bonds of solidarity to educate entire villages on the importance of identifying individuals with symptoms of Ebola and seeking early diagnosis and referral to health facilities.
Capps	Evaluation of a programme to train village health workers in El Salvador	1989	Columbia University, Holy Cross Hospital	Journal article	Health Policy and Planning	Americas	El Salvador	Conflict	General population	Child health, infectious diseases, WASH, first aid	Treatment of pneumonia, diarrhea, malaria, and malnutrition; first aid; WASH promotion	Village health promoters	Documentation of experience, survey of CHW attitudes and practices, review of CHW reports	− CHWs were trained to overcome a lack of access to health services through health facilities.<br>− Challenges in maintaining regular supervision due to distances, insecurity, and fear of gathering in large groups.<br>− CHWs did not feel they received the support they needed to work as effectively as possible.<br>− There was a preference among CHWs and community members for curative services over health promotion activities.
Carter	Treatment Seeking and Ebola Community Care Centers in Sierra Leone: A Qualitative Study	2017	Oxfam International	Journal article	Journal of Health Communication	Africa	Sierra Leone	Disease outbreak	General population	Infectious diseases	Community outreach, promotion of care seeking	Community health volunteers	Qualitative interviews, focus group discussions	− Community health volunteers served as a link between communities and Ebola community care centers. The CHVs would share information about community care centers and encourage people to seek care and bring back information on family members who were in community care centers.

Caulker	Life goes on: the resilience of maternal primary care during the Ebola outbreak in rural Sierra Leone	2017	Sierra Leone Ministry of Health and Sanitation, University of Toronto, Institute of Tropical Medicine, Makerere University, Baroda Medical College, Dignitas International	Journal article	Public Health Action	Africa	Sierra Leone	Disease outbreak	General population	Infectious diseases	Contact tracing	Community health workers	Document review, analysis of quantitative program data	− CHWs were diverted from routine activities to Ebola contact tracing.
Chaiken	The promise of a community-based approach to managing severe malnutrition: A case study from Ethiopia	2006	Indiana University of Pennsylvania, Save the Children	Journal article	Food and Nutrition Bulletin	Africa	Ethiopia	Nutrition emergenc y	General population	Nutrition	Malnutrition screening, health education	Outreach workers	Analysis of quantitative program data	− Community outreach workers were used to screen for malnutrition and carry out follow-up visits during a nutrition emergency.<br>− High cure rates and low default rates were attributed to the intensive monitoring of children in the community by the outreach workers.
Chase	Building back better? Taking stock of the post-earthquake mental health and psychosocial response in Nepal	2018	SOAS University of London, World Health Organization, Psychbigyaan Network Nepal, Transcultural Psychosocial Organization Nepal, Nepal Ministry of Health and Population	Journal article	International Journal of Mental Health Systems	South-East Asia	Nepal	Natural disaster	General population	Mental health	Psychosocial counseling and support	Female community health volunteers	Literature review, focus group discussions	− Pre-existing female community health volunteers were trained to provide psychosocial counseling and support to earthquake survivors in communities.
Chaudhary	Humanitarian response to reproductive and sexual health needs in a disaster: the Nepal Earthquake 2015 case study	2017	Nepal Ministry of Health, United Nations Population Fund, World Health Organization, Deutsche Gesellschaft fuer internationale Zusammenarbeit, Midwifery Society of Nepal, United Nations Children's Fund	Journal article	Reproductive Health Matters	South-East Asia	Nepal	Natural disaster	General population	Sexual and reproductive health	Community mobilization, distribution of items to community members	Female community health volunteers	Literature review	− Female community health volunteers carried out community mobilization and distribution of reproductive health items as part of the earthquake response.
Chen	Reproductive health for refugees by refugees in Guinea II: sexually transmitted infections	2008	London School of Hygiene and Tropical Medicine, Gesellschaft für Technische Zusammenarbeit, Reproductive Health Group	Journal article	Conflict and Health	Africa	Guinea	Conflict	Camp	Sexual and reproductive health, infectious diseases	Provision of contraceptives, health education	Lay health workers	Survey of men and women	− Refugee lay health workers provided contraceptives and health education in refugee camps.

Chi	The evolving role of traditional birth attendants in maternal health in post-conflict Africa: A qualitative study of Burundi and northern Uganda	2018	Karolinska Institutet, Peace Research Institute Oslo	Journal article	SAGE Open Medicine	Africa	Burundi, Uganda	Conflict	General population	Maternal health	Assisted home delivery, newborn care	Traditional birth attendants	Qualitative interviews, focus group discussions	− In response to decreased access to facility-based care during conflict, TBAs were trained and supplied to assist childbirth in communities.<br>− TBAs were able to support deliveries during acute conflict when there was no access to facilities and when communities were displaced.<br>− Some respondents associated increased utilization of TBAs for delivery with high levels of maternal and neonatal mortality and morbidity due to TBAs' low level of competence.
Coleridge	Community Based Rehabilitation in a Complex Emergency: Study of Afghanistan	2002	International Labor Organization	Journal article	Asia Pacific Disability Rehabilitation Journal	Eastern Mediterran ean	Afghanist an	Conflict	General population	Injury rehabilitation	Community mobilization, identification and referral of disabled people, vocational training	Community rehabilitation and development workers, volunteers	Documentation of experience	− Community rehabilitation and development workers and volunteers carried out community mobilization, identification and referral of disabled people, vocational training in communities.
Contreras	Community strengthening and mental health system linking after flooding in two informal human settlements in Peru: a model for small-scale disaster response	2018	Socios En Salud, Harvard Medical School	Journal article	Global Mental Health	Americas	Peru	Natural disaster	General population	Mental health	Screening and referral for depression and domestic violence, community theater	Community health workers, volunteers	Documentation of experience	− CHWs carried out community-based screening and referral for depression and domestic violence.
Corbeau	Mission in the Afghan camps	1989	Association Internationale Contre La Faim, United Nations High Commissioner for Refugees	Journal article	Revue de l'Infirmiere	Eastern Mediterran ean	Afghanist an	Conflict	Camp	Maternal health, newborn health	Assisted home delivery, newborn care	Female community health workers	Documentation of experience	− Traditional midwives were trained to support deliveries in Afghan refugee camps.<br>− Training methodologies had to be adapted to the conservative traditional norms.
Corish	Safe motherhood in a refugee setting	1997	Not specified	Journal article	Safe Motherhood Newsletter	Africa	Kenya	Conflict	Camp	Maternal health	Assisted home delivery, newborn care	Community health workers, traditional birth attendants	Documentation of experience	− Refugee TBAs were trained to assist deliveries in the refugee camp.<br>− Trained TBAs faced harassment and threats by untrained TBAs who felt that the trained TBAs threatened their livelihoods.
Cropley	The effect of health education interventions on child malaria treatment-seeking practices among mothers in rural refugee villages in Belize, Central America	2004	University of New Orleans	Journal article	Health Promotion International	Americas	Belize	Not specified	General population, camp	Child health	Treatment of malaria, health education, disease surveillance	Voluntary collaborators	Quasi-experimental study	− Voluntary collaborators provided education on prevention of malaria and importance of care seeking and provided malaria treatment.<br>− Despite high knowledge of availability of malaria treatment by voluntary collaborators, utilization was low.

Crowe	A Plan for Community Event-Based Surveillance to Reduce Ebola Transmission — Sierra Leone, 2014–2015	2015	U.S. Centers for Disease Control and Prevention, International Rescue Committee, Sierra Leone Ministry of Health and Sanitation, World Health Organization	Journal article	Morbidity and Mortality Weekly Report	Africa	Sierra Leone	Disease outbreak	General population	Infectious diseases	Community events-based surveillance	Community health monitors	Documentation of experience	− Community members were trained to carry out community events-based surveillance for Ebola.<br>− Preliminary assessments indicated that the program had a high level of acceptance by key community leaders, villagers, and the case detection and response team members.
Cutts	Training Community Health Workers in refugee camps: A case study from Pakistan	1984	London School of Hygiene and Tropical Medicine	Journal article	Disasters	Eastern Mediterranean	Pakistan	Conflict	Camp	Maternal health, child health, infectious diseases	Health education; screening for TB, malaria, and malnutrition; immunization promotion; first aid; registration of births and deaths	Community health workers, Dais	Documentation of experience	− CHWs and Dais are trained to provide health education; identifying cases of TB, malaria and malnutrition; immunization promotion; first aid; and registrations of births and deaths in Afghan refugee camps in Pakistan.<br>− Training women was a sensitive issue due to the cultural background.
D'Agnes	Community-based approach to refugee relief: experiences from Thailand	1982	Community Based Emergency Relief Services	Journal article	International Planned Parenthood Medical Bulletin	South-East Asia	Thailand	Conflict	Camp	Reproductive health	Provision of contraceptives, promotion of family planning	Community volunteers	Documentation of experience	− Community volunteers provided contraceptives to Cambodian refugees.
Deressa	Community participation in malaria epidemic control in highland areas of southern Oromia, Ethiopia	2005	Addis Ababa University, Oromia Regional State Health Bureau	Journal article	Ethiopian Journal of Health and Development	Africa	Ethiopia	Disease outbreak	General population	Child health, newborn health, maternal health	Diagnosis and treatment of uncomplicated malaria, referral of severe malaria cases, community mobilization and health education, environmental management, surveillance, death registration	Village malaria workers	Document review	− CHWs were recruited and quickly trained to carry out malaria treatment and prevention activities, as well as surveillance of malaria cases and deaths, during a malaria epidemic.<br>− CHWs were supervised on a weekly basis, which allowed for frequent support, supply of medications, and collection of surveillance reports.<br>− CHWs treated large numbers of people and reported many deaths.
Doherty	Providing Palliative Care in a Humanitarian Crisis Situation: Using Community Health Workers in Rohingya Refugee Camps	2018	World Child Cancer and Fasiuddin Khan Research Foundation	Web blog	N/A	South-East Asia	Bangladesh	Conflict	Camp	Palliative care	Palliative care	Palliative care assistants	Documentation of experience	− Palliative care assistants (PCAs) provided basic palliative care to patients with chronic or life-threatening illnesses in their homes.<br>− The PCAs support patients by delivering medications, providing basic nursing care, and monitoring symptoms.<br>− Patients are referred to PCAs from a health center or through active case finding in the community by PCAs.
Dudgeon	Birth after Death: Men and Reproduction in Two K'iche' Maya Communities	2013	Emory University	Dissertation	N/A	Americas	Guatemala	Conflict	General population	Reproductive health, maternal health, newborn health	Antenatal care	Traditional birth attendants	Household surveys, participant observation	− Prenatal care was provided to the Maya communities after the Guatemalan civil war mainly by TBAs.

Edmond	Can community health worker home visiting improve care-seeking and maternal and newborn care practices in fragile states such as Afghanistan? A population-based intervention study	2018	United Nations Children's Fund, Afghanistan Ministry of Public Health, Save The Children, Harvard School of Public Health	Journal article	BMC Medicine	Eastern Mediterranean	Afghanistan	Conflict	General population	Maternal health, newborn health	Antenatal care; postnatal care; provision of contraceptives; treatment of pneumonia and diarrhea; health education; reporting pregnancies, births, and deaths	Community health workers	Quasi-experimental study	− CHWs were trained to conduct home visits and provide behavior change communication with pregnant or postpartum women.<br>− The intervention resulted in improved care seeking and service utilization for facility delivery.<br>− The levels of conflict and insecurity restricted movement of some CHWs.<br>− Cultural practices limited access to some mothers.<br>− Competing workload priorities and other employment also restricted the time the CHWs could devote to the home visiting program.
Edward	Toward universal coverage in Afghanistan: A multi-stakeholder assessment of capacity investments in the community health worker system	2015	Johns Hopkins University, Management Sciences for Health, Texas Department of State Health Services, Babson College, Afghanistan Ministry of Public Health	Journal article	Social Science and Medicine	Eastern Mediterranean	Afghanistan	Conflict	General population	Child health, maternal health, reproductive health	Treatment of childhood illnesses, provision of contraceptives, health promotion, demand-creation for health services	Community health workers	Survey of health facilities, qualitative interviews, focus group discussions; capacity assessments of CHW health posts	− Support to CHWs in terms of training, supervision and supplies were adequate.<br>− Keys to the success of the program were strong community engagement and community support of CHWs, strong MoH leadership on community health, and adapting CHW services to the cultural context.<br>− Lack of transport, weather and security challenges, human resources for supervision, and stock-outs of commodities were key challenges.
Fallah	Interrupting Ebola Transmission in Liberia Through Community-Based Initiatives	2016	Liberia Ministry of Health, United Nations Development Programme, University of Liberia, Yale School of Public Health	Journal article	Annals of Internal Medicine	Africa	Liberia	Disease outbreak	General population	Infectious diseases	Community events-based surveillance	Not specified	Documentation of experience	− The initial top-down Ebola response that did not take into account the cultural context was not successful.<br>− In a setting where trust for government is low, but trust for community leaders is high, it was crucial to engage community leaders in the response.<br>− Community leaders recruited community members to be trained on active surveillance.<br>− The community-based surveillance intervention quickly increased the number of Ebola cases detected and isolated.<br>− The success of the intervention in West Point led to its replication in Montserrado County, leading to increased case detection and faster isolation.
Ferreyra	Implementing test and start programme in a rural conflict affected area of South Sudan: the experience of Médecins Sans Frontières	2017	Médecins Sans Frontières, South Sudan Ministry of Health	Conference abstract	Journal of the International AIDS Society	Africa	South Sudan	Conflict	General population	Infectious diseases	Distribution of treatment for AIDS	Community health workers	Analysis of quantitative program data	− The contingency plan for an HIV/AIDS program included coordinating with CHWs to distribute ‘run-away bags’ with 3 months of ART for people needing treatment during periods of conflict.

Fredricks	Community Health Workers and Disasters: Lessons Learned from the 2015 Earthquake in Nepal	2017	Massachusetts General Hospital, Harvard University, Dhulikhel Hospital, Kathmandu University	Journal article	Prehospital and Disaster Medicine	South-East Asia	Nepal	Natural disaster	General population	Maternal health, newborn health, child health, nutrition, family planning, WASH	Treatment of childhood diarrhea and malnutrition, provision of contraceptives, health promotion and education, growth monitoring, first aid	Female community health volunteers	Qualitative interviews, focus group discussions	− External relief arrived to remote communities weeks after the earthquake.<br>− Without external support, CHWs immediately carried out relief activities following the earthquake.<br>− CHWs did not receive any emergency preparedness training and did not feel prepared when the disaster happened.<br>− Some aid agencies used CHWs to plan and deliver relief services in their communities, but most did not engage the CHWs.
Garfield	Health education and community participation in mass drug administration for malaria in Nicaragua	1986	Columbia University	Journal article	Social Science and Medicine	Americas	Nicaragua	Conflict	General population	Infectious diseases	Treatment of malaria, health education, household registration	Brigadistas	Documentation of experience	− Community-based brigadistas supported a mass anti-malarial treatment campaign by conducting a household census, providing health education, and providing malaria treatments.
Geiger	A new medical mission to El Salvador	1989	City University of New York, Harvard Medical School, University of Washington, University of California Los Angeles, Wisconsin Department of Health, Harvard Center for Population Studies, Physicians for Human Rights	Journal article	New England Journal of Medicine	Americas	El Salvador	Conflict	General population	Not specified	Simple preventive and curative care (not specified)	Community health promoters	Documentation of experience, qualitative interviews	− Community health promoters provided preventive and curative care in conflict settings.<br>− Health workers were faced with a variety of obstacles, including lack of access and security issues.<br>− The military limited movement of commodities and interfered with immunization campaigns.
George	Ongoing Cholera Outbreak — Kenya, 2014–2016	2016	Kenya Ministry of Health, US Centers for Disease Control and Prevention	Journal article	Morbidity and Mortality Weekly Report	Africa	Kenya	Disease outbreak	General population	Infectious diseases	Health education, distribution of supplies	Community health extension workers	Documentation of experience	− Community health extension workers were integral to the promotion of cholera prevention messaging and distribution of supplies.
Ghebreyes us	Community participation in malaria control in Tigray region Ethiopia	1996	Ethiopia National Malaria Control Organization, World Health Organization	Journal article	Acta Tropica	Africa	Ethiopia	Conflict	General population	Child health, maternal health, nutrition, WASH	Treatment of malaria, environmental management	Community health workers, traditional birth attendants	N/A	N/A
Gilmartin	South Sudan Boma Health Initiative Costing and Investment Case Analysis	2018	Management Sciences for Health	Report	N/A	Africa	South Sudan	Conflict	General population	Maternal health, newborn health, child health	Treatment of childhood pneumonia, diarrhea, and malaria; bednet distribution; promotion of ANC and PNC; distribution of contraceptives; community mobilization	Community health workers	Document analysis, modeling of costs of scale-up, qualitative interviews	− In South Sudan, insecurity, along with poor management, high distribution costs, and inadequate storage facilities, led to stockouts of iCCM commodities. This reduces utilisation and damages community and CHW confidence.

Gopalan	Maternal and neonatal service usage and determinants in fragile and conflict-affected situations: a systematic review of Asia and the Middle-East	2017	London School of Hygiene and Tropical Medicine, The World Bank	Journal article	BMC Women's Health	Various	Various	Conflict	General population	Maternal health, newborn health	Assisted home delivery	Community health workers, traditional birth attendants	Literature review	− TBAs provided maternal and neonatal health services and provided assistance in deliveries in numerous countries within conflict settings.<br>− Presence of a female TBA or CHW increased the possibility of receiving skilled birth attandance.
Grainger	Providing safe motherhood services to underserved and neglected populations in Yemen: the case for vouchers	2017	Yemen Reproductive Health Programme, Yemen Reproductive Health Voucher Programme, Marie Stopes International	Journal article	Journal of International Humanitarian Action	Eastern Mediterranean	Yemen	Conflict	General population	Maternal health, newborn health	Distribution of vouchers	Community midwives	Documentation of experience, analysis of quantitative program data	− Community midwives were utilized to distribute vouchers for maternal and newborn health services.
Gulzar	Role of community health nurse in earthquake affected areas	2012	Aga Khan University School of Nursing and Midwifery, The Aman Foundation	Journal article	Journal of Pakistan Medical Association	Eastern Mediterranean	Pakistan	Natural disaster	General population	Not specified	Curative care (not specified), health education	Community health nurses, lady health visitors, community health workers	Documentation of experience	− Community-based health workers were trained to provide curative care and health promotion activities following an earthquake.
Gupta	Importance of surveillance for early detection of a cholera outbreak	1993	National Institute of Cholera and Enteric Diseases	Journal article	Journal of Communicable Diseases	South-East Asia	India	Disease outbreak	General population	Infectious diseases	Treatment of diarrhea, health education, disease surveillance	Village health workers	Documentation of experience	− VHWs were trained to prepare ORS and recognize signs of dehydration.<br>− A cholera outbreak was detected early due to an ongoing surveillance system through VHWs.
Gyo	Humanitarian struggles in Burma's conflict zones	2017	Back Pack Health Worker Team	Journal article	Special Operations Medical Journal	South-East Asia	Myanmar	Conflict	General population	Maternal health, newborn health, child health, family planning, nutrition, WASH	Treatment of pneumonia, diarrhea, malaria, helminth infection, and anemia; trauma care; assisted home delivery; antenatal care; postnatal care; birth registration; contraceptive distribution; health education; installation of latrines and water systems; distribution of personal hygiene kits; distribution of nutritional supplements; disease surveillance	Community health workers, traditional birth attendants, village health workers	Documentation of experience	− A flexible system with mobile CHWs who are supported from outside the country, in addition to local volunteer health workers and TBAs has been able to provide continuity of health services in conflict-affected areas.<br>− The services provided become more limited as the intensity of conflict in an area increases.<br>− CHWs are able to move with communities when there is population displacement.<br>− Mobile CHWs face a number of security risks, including violence by armed forces, landmines, and adverse weather. CHWs' local knowledge as members of communities they serve helps to mitigate the risk.<br>− The program operates with infrequent supervision and re-supply of commodities (every 6 months).

Habboush	The motivational factors for Community Health Workers (CHWs) in the Syrian conflict context	2018	University of Birmingham	Dissertation	N/A	Eastern Mediterranean	Syria	Conflict	General population	Not specified	Not specified	Community health workers	Qualitative interviews	− Respondents reported that: 1) recruiting CHWs from their home communities improved trust from community members and CHW motivation, 2) highly qualified professionals recruited as CHWs were more likely to leave the position for an alternative job, 3) conducting trainings was challenging in the conflict setting, 4) availability of supplies was an important factor affecting the motivation of CHWs.
Hadi	Raising institutional delivery in war-torn communities: Experience of BRAC in Afghanistan	2007	Bangladesh Rural Advancement Committee	Journal article	Asia Pacific Journal of Family Medicine	Eastern Mediterranean	Afghanistan	Conflict	General population	Maternal health	Health education, promotion of antenatal care, treatment of common childhood illnesses, promotion of facility delivery, promotion of family planning	Community health workers	Pre-post household surveys	− Male CHWs were tasked with raising awareness among men and community leaders regarding the needs of reproductive health services, immunization, and family planning.<br>− Female CHWs were trained to raise health awareness among women, identify pregnant women and motivate them to receive antenatal care services, identify sick children, provide treatment of common illnesses, promote the benefits of having institutional delivery and refer complicated cases to the nearby clinics or hospitals.<br>− CHWs reached 95.5% of pregnant women in 2 years, up from 40.3%.<br>− The proportion of pregnant women who received antenatal care rose from 37.3% to 91.2%.<br>− CHWs were also responsible for calling an ambulance in difficult pregnancy cases.<br>− CHWs also served as social marketing agents, selling health products such as ORS, iodized salt, safe delivery kits, and contraceptives.
Hanscom	Treating Survivors of War Trauma and Torture	2001	Advocates for Survivors of Torture and Trauma	Journal article	American Psychologist	Americas	Guatemala	Conflict	General population	Mental health	Psychosocial support	Lay counselors, midwives	Documentation of experience	− Lay counselors and midwives were trained to provide psychosocial support to survivors of human rights abuses.
Harrison	'Against all odds': UNHCR's mental health and psychosocial support programme for Iraqi refugees and internally displaced Syrians	2013	United Nations High Commissioner for Refugees,	Journal article	Intervention	Eastern Mediterranean	Syria	Conflict	Camp	Mental health	Psychosocial support	Psychosocial outreach volunteers	Documentation of experience	− Psychosocial outreach volunteers were a part of a program to help improve the psychosocial wellbeing and mental health of vulnerable refugees and Syrians affected by conflict.<br>− A pre-existing program for Iraqi refugees in Syria was expanded to support Syrians as conflict worsened in Syria.<br>− Outreach volunteers also provided social, recreational, educational and livelihood activities.<br>− Security concerns limited the access of outreach volunteers.

Hassan	Personal reflections on a psychosocial community outreach programme and centre in Damascus, Syria	2013	United Nations High Commissioner for Refugees	Journal article	Intervention	Eastern Mediterranean	Syria	Conflict	Camp	Mental health	Psychosocial support	Psychosocial outreach volunteers	Documentation of experience	− Psychosocial outreach volunteers served as a connection between the community and the non-governmental organization.<br>− Volunteers faced cultural barriers as not all families accepted volunteers in their homes.
Hawkes	Use and limitations of malaria rapid diagnostic testing by community health workers in war-torn Democratic Republic of Congo	2009	University of Toronto, Hospital for Sick Children, Institut Supérieur des Techniques Médicales de Kisangani, HEAL Africa	Journal article	Malaria Journal	Africa	Democratic Republic of Congo	Conflict	General population	Child health	Treatment of malaria	Community health workers	Prospective cohort study, satisfaction questionnaire and decision analysis	− It was feasible to train and deploy CHWs to use RDTs to diagnose malaria in an insecure setting.
Heggenhougen	Will primary health care efforts be allowed to succeed?	1984	London School of Hygiene and Tropical Medicine	Journal article	Social Science and Medicine	Americas	Guatemala	Conflict	General population	Maternal health, child health	Not specified	Promotores de salud	Documentation of experience	− In Guatemala, many CHWs and members of their families were targeted and killed.<br>− Community members said they trusted the village health workers from their communities and understood the treatments since they were communicated to them in a way they could understand.
Howard	Reproductive health services for refugees by refugees in Guinea I: family planning	2008	London School of Hygiene and Tropical Medicine, Reproductive Health Group, Gesellschaft für Technische Zusammenarbeit	Journal article	Conflict and Health	Africa	Guinea	Conflict	Camp	Reproductive health	Provision of contraceptives, health education	Lay health workers	Survey of men and women	− Refugee lay health workers provided contraceptives and health education in refugee camps
Howard	Reproductive health for refugees by refugees in Guinea III: maternal health	2011	London School of Hygiene and Tropical Medicine, Reproductive Health Group, Gesellschaft für Technische Zusammenarbeit, Institute of Tropical Medicine and International Health, Institute of Tropical Medicine	Journal article	Conflict and Health	Africa	Guinea	Conflict	Camp	Maternal health	Provision of contraceptives, health education	Lay health workers	Survey of men and women	− Refugee lay health workers provided contraceptives and health education in refugee camps
Huber	Achieving success with family planning in rural Afghanistan	2010	Management Sciences for Health, United Nations Development Programme, STEP Health and Development Organization	Journal article	Bulletin of the World Health Organization	Eastern Mediterranean	Afghanistan	Conflict	General population	Reproductive health	Provision of contraceptives, promotion of family planning	Community health workers	Pre-post LQAS household surveys	− CHWs provided injectable contraceptives, pills, and condoms in the community.<br>− The contraceptive prevalence rate increased by 24–27% in 8 months in the project areas.<br>− Meetings with religious and other community leaders, including health committees, were important for developing trust and obtaining acceptance of innovations, such as permitting CHWs to initiate injectable contraceptives.

Husum	Training pre-hospital trauma care in low-income countries: the 'Village University' experience	2009	University Hospital of Northern Norway, Hammerfest Hospital	Journal article	Medical Teacher	Eastern Mediterranean, Western Pacific	Iraq, Cambodia	Conflict	General population	Trauma	Trauma care	Village first responders	Analysis of patient medical records, patient survey	− Village first responders were trained to provide emergency trauma care for victims of landmines in communities.<br>− The mortality rate for landmine victims in the program areas was 21.2%, down from a pre-intervention mortality rate estimated of 40%.
Hynes	Reproductive health indicators and outcomes among refugee and internally displaced persons in postemergency phase camps	2002	U.S. Centers for Disease Control and Prevention, Johns Hopkins School of Public Health	Journal article	Journal of the American Medical Association	Various	Various	Conflict	Camp	Reproductive health	Not specified	Community health workers	Analysis of survey data, analysis of routine program data, qualitative interviews	− Camps with more CHWs had a lower percentage of newborns with low birth weight.
International Federation of Red Cross and Red Crescent Societies	Reproductive, maternal, newborn and child health today: Overview and implementation analysis	2014	International Federation of the Red Cross and Red Crescent Societies	Report	N/A	Eastern Mediterranean, Americas	Afghanistan, Haiti, Somalia	Conflict, natural disaster	General population	Maternal health, newborn health, child health	Treatment of childhood pneumonia, diarrhea, and malaria; malnutrition screening; health education; screening and referral of pregnant women and newborns	Red Cross/Red Crescent volunteers, health promoters	Document analysis	− In Afghanistan, recruitment of female volunteers and female trainers to train female volunteers increased access to health services for women.<br>− In Haiti, most Red Cross volunteers ceased their activities following the earthquake.<br>− In Somalia, Red Crescent has stopped immunization activities for several years because the militant group in the area refused to allow house-to-house visits or mass public campaign activities.
International Federation of Red Cross and Red Crescent Societies	CBHFA Asia Pacific Workshop- Lessons Learnt and Monitoring and Evaluation	2010	International Federation of the Red Cross and Red Crescent Societies	Conference paper	N/A	South-East Asia	Various	Not specified	Not specified	Not specified	Not specified	Red Cross/Red Crescent volunteers	Summary of presentations and discussion	− IFRC review of experiences found that volunteer protection needs to be included in the volunteer training. They should also be able to provide psychosocial support to volunteers who experience traumatic situations. They also offer volunteer insurance through IFRC.
International Rescue Committee	Enabling treatment of severe acute malnutrition in the community: Study of a simplified algorithm and tools in South Sudan	2018	International Rescue Committee	Report	N/A	Africa	South Sudan	Conflict	General population	Child health	Treatment of childhood malnutrition	Community based distributors	Direct observation of case management, qualitative interviews, focus group discussions, cost analysis	− Special tools for low-literacy CHWs to diagnose and treat childhood malnutrition in a setting of insecurity. The CHWs showed high levels of accuracy in treating malnourished children with these tools.
Ikram	Communicable disease control in Afghanistan	2013	US Agency for International Development, World Health Organization, Malaysian Armed Forces	Journal article	Global Public Health	Eastern Mediterranean	Afghanistan	Conflict	General population	Infectious diseases	Active case finding, DOTS for TB, health education	Community health workers	Documentation of experience, analysis of quantitative program data, qualitative interviews	− CHWs supported the national TB program through active case finding, provision of DOTS, and health education.
Imaja	A design of a mobile health intervention for the prevention and treatment of Cholera in South Kivu in DRC	2017	University of KwaZulu-Natal	Conference paper	IEEE Global Humanitarian Technology Conference	Africa	Democratic Republic of Congo	Disease outbreak	General population	Infectious diseases	Health education, disease surveillance	Community health workers	Documentation of experience	− CHWs provided health education on cholera prevention and conduct surveillance for cholera cases.

Jackson	War and community health in the Philippines	1989	Christian Aid Asia/Pacific	Journal article	Health Visitor	Western Pacific	Philippines	Conflict	General population	Child health	Treatment of infectious diseases	Community health workers	Documentation of experience	− CHWs were harassed and targeted for violence because of perceived political affiliations. − CHWs often used traditional or homemade medicines to increase community self-reliance and to reduce costs in communities that were persecuted by the government.
Janneck	Human Resources in Humanitarian Health Working Group Report	2009	Harvard School of Public Health, The George Washington University, Boston University, Albert Einstein College of Medicine	Journal article	Prehospital and Disaster Medicine	Various	Various	Conflict, natural disaster	Not specified	Not specified	Not specified	Community health workers	Documentation of experience	− Involving CHWs in service and health delivery in humanitarian settings can extend the reach of healthcare and improve recruitment, retention and local capacity. − CHWs need to be properly compensated to alleviate personal and financial stress.
Jayatissa	Community-based management of severe and moderate acute malnutrition during emergencies in Sri Lanka: Challenges of implementation	2012	Sri Lanka Ministry of Health, United Nations Children's Fund	Journal article	Food and Nutrition Bulletin	South-East Asia	Sri Lanka	Conflict, nutrition emergency	General population, camp	Nutrition	Community mobilization	Health volunteers	Pre-post nutrition surveys	− Community volunteers were used for community mobilization for a community nutrition campaign during a nutrition emergency.
Jones	Rebuilding people-centred maternal health services in post-Ebola Liberia through participatory action research	2018	International Rescue Committee, London School of Hygiene and Tropical Medicine, Redemption Hospital, Training and Research Support Centre	Journal article	Global Public Health	Africa	Liberia	Disease outbreak	General population	Maternal health	Promotion of facility delivery, assisted home delivery	Trained traditional midwives	Group discussions, surveys of key informants	− During the Ebola outbreak, TTMs' role shifted from promotion of facility delivery to attending deliveries to fill the gaps in delivery care. − The TTMs and TBAs were the first points of care for pregnant and delivering women, carrying out most deliveries and continuing to do so even after the Ebola epidemic. − The TTMs and TBAs expressed their anger at not being recognized for providing care during Ebola.
Joseph	Tungiasis in rural Haiti: a community-based response	2006	Brigham and Women's Hospital, Zanmi Lasante, Partners in Health	Journal article	Transactions of the Royal Society of Tropical Medicine and Hygiene	Americas	Haiti	Disease outbreak	General population	Infectious diseases	Immunization, provision of DOTS for TB, DOT for AIDS, disease surveillance, treatment of tungiasis, health education	Community health workers	Documentation of experience	− CHWs were the first to report an outbreak of tungiasis to clinic staff. − CHWs informed the community about the disease and provided treatment.
Kasi	A Decade of Disasters: Lessons from the Indian Experience	2007	National Institute of Mental Health and Neuro Sciences, American Red Cross, East Tennessee State University	Journal article	Southern Medical Journal	South-East Asia	India	Conflict, natural disaster	General population	Mental health	Psychosocial support	Community level workers	Documentation of experience	− Community level workers were trained to respond to the psychosocial needs of the survivors of disasters.

Keller	Evaluative Research in a Refugee Camp: The Effectiveness of Community Health Workers in Khao I Dang - Holding Center, Thailand	1988	Not specified	Journal article	Disasters	South-East Asia	Thailand	Conflict	Camp	Not specified	Case finding, simple curative treatments, immunization, antenatal care, postnatal care, family planning, health education	Community health workers	Documentation of experience	− Community members preferred older female CHWs rather than the young male CHWs that were hired. Men and young people were not culturally acceptable to perform the role.<br>− Class conflicts were identified between educated, upper-class CHWs and the uneducated camp majority.<br>− Poor community acceptance of the CHWs impaired their effectiveness, and was attributed to age, sex and socioeconomic barriers.
Khan	Evaluating feasibility and acceptability of a local psycho-educational intervention for pregnant women with common mental problems affected by armed conflict in Swat, Pakistan: A parallel randomized controlled feasibility trial	2017	Khyber Medical University, University of Liverpool, Human Development Research Foundation	Journal article	International Journal of Social Psychiatry	Eastern Mediterranean	Pakistan	Conflict	General population	Mental health, maternal health	Psychosocial support	Lady health workers	Randomized controlled trial, qualitative interviews	− LHWs were the preferred source of information on women's health concerns.<br>− LHWs were able to deliver the psychosocial intervention, and they expressed satisfaction at understanding more about the mental health needs of pregnant women.<br>− The intervention was acceptable to the community and as to the LHWs who delivered it.<br>− Psychological distress was lower in the intervention than in the control arm, but this was not statistically significant.
Khan	Newborn survival in Pakistan: a decade of change and future implications	2012	Save the Children, Pakistan Institute of Medical Sciences, Health Services Academy, John Snow, Inc., The Aga Khan University	Journal article	Health Policy and Planning	Eastern Mediterranean	Pakistan	Conflict	General population	Maternal health, newborn health, child health	Maternal and child health services (not specified), preventative services, (not specified) referral and link to skilled birth attendants	Lady health workers, community health workers	Literature review	− The LHWs played a key role following the earthquake of 2005 with provision of family health services.<br>− A number of important research studies demonstrated that simple community-based interventions increased newborn survival and improved newborn care practices.<br>− LHWs helped increased immunization coverage for tetanus elimination.<br>− LHWs had shortcomings in terms of knowledge and skills, as well as absenteeism and lack of equipment.
Kim	Evaluation of an Interactive Electronic Health Education Tool in Rural Afghanistan	2008	VA Boston Healthcare System, Brigham and Women's Hospital and Harvard Medical School, International Medical Corps, Johns Hopkins University, F. Edward Hébert School of Medicine	Journal article	Prehospital and Disaster Medicine	Eastern Mediterranean	Afghanistan	Conflict	General population	Not specified	Health education	Community health workers	Pre-post household surveys	− Both an interactive electronic health education tool and health education by CHWs were effective in increasing community health knowledge.<br>− CHWs were preferred by Afghans because of the value placed on personal contact and interaction.
Kodish	Understanding low usage of micronutrient powder in the Kakuma Refugee Camp, Kenya: Findings from a qualitative study	2011	Johns Hopkins Bloomberg School of Public Health, Sight and Life, World Food Programme, Tufts University	Journal article	Food and Nutrition Bulletin	Africa	Kenya	Conflict	Camp	Nutrition	Distribution of food rations and micronutrient powders	Community health workers	Qualitative interviews, focus group discussions, direct observation	− CHWs distributed food rations and micronutrient powders in a refugee camp.<br>− CHWs were inadequately trained, which led to inadequate communication regarding the health benefits and use of micronutrient powder to the beneficiaries.

Kodish	Implications of the Ebola virus disease outbreak in Guinea: Qualitative findings to inform future health and nutrition-related responses	2018	GroundWork, United Nations Children's Fund, Guinea Ministry of Health	Journal article	PLOS One	Africa	Guinea	Disease outbreak	General population	Nutrition	Health education	Community health workers	Qualitative interviews	− CHWs delivered nutrition-related social and behavioral change communication during the Ebola outbreak.
Koenig	Tuberculosis in the aftermath of the 2010 earthquake in Haiti	2015	Haitian Study Group for Kaposi's Sarcoma and Opportunistic Infections, Haitian National Tuberculosis Program, Stanford University	Journal article	Bulletin of the World Health Organization	Americas	Haiti	Natural disaster	General population, camp	Infectious diseases	Active case finding	Community health workers	Documentation of experience, analysis of quantitative program data	− Community health workers conducted active case finding for TB following the Haiti earthquake.
Kohli	A Congolese community-based health program for survivors of sexual violence	2012	Johns Hopkins Bloomberg School of Public Health, Foundation RamaLevina, Johns Hopkins University School of Nursing, Johns Hopkins Center for Global Health	Journal article	Conflict and Health	Africa	Democratic Republic of Congo	Conflict	General population	Gender-based violence	Community mobilization, community education	Community health workers	Documentation of experience	− CHWs supported a program to bring health services to GBV survivors through mobile clinics.<br>− CHWs assisted providers in prioritizing services based on women's report of symptoms.<br>− CHWs informed communities of the services available and the mobile clinic schedule.<br>− CHWs helped with prioritization of services and community education topics based on their knowledge of the communities.
Kolaczinski	Adherence of community caretakers of children to pre-packaged antimalarial medicines (HOMAPAK) among internally displaced people in Gulu district, Uganda	2006	Malaria Consortium, London School of Hygiene & Tropical Medicine, Gulu District Health Services	Journal article	Malaria Journal	Africa	Uganda	Conflict	Camp	Child health	Treatment of malaria	Community drug distributors	Household survey	− CHWs were able to correctly prescribe antimalarial medication for sick children in an IDP camp setting.<br>− Most caretakers sought care within 24 hours for children falling ill with fever.

Kozuki	The resilience of integrated community case management in acute emergency: A case study from Unity State, South Sudan	2018	International Rescue Committee, United Nations Children's Fund	Journal article	Journal of Global Health	Africa	South Sudan	Conflict	General population	Child health	Treatment of childhood pneumonia, diarrhea, and malaria	Community based distributors	Qualitative interviews, focus group discussions, analysis of quantitative program data	− The large majority of CHWs continued their work during the crisis period.<br>− The number of children treated declined by about 50% at the peak of the crisis, but then quickly recovered. The number of children treated by CHWs was consistently higher than by health facilities, before, during, and after the crisis.<br>− Supervisors were able to use community networks to assess the security situation in specific areas and determine whether it was safe to travel and to track down CHWs to provide supervision and resupply them with drugs.<br>− CHWs often moved with communities when they were displaced and continued treating sick children. CHWs in areas that received IDPs treated both their original host population and IDPs. Displaced CHWs also treated their displaced community members and the host population.<br>− CHWs in areas that experienced a large influx of IDPs were not able to meet the demand for services due to limited drug supplies.<br>− Caregivers preferred to seek care in the community to avoid traveling in insecure areas and because of a perception that health facilities were closed or lacked essential medicines.

Kozuki	Integrated Community Case Management in Acute and Protracted Emergencies Case study for South Sudan	2017	International Rescue Committee, United Nations Children's Fund	Report	N/A	Africa	South Sudan	Conflict	General population	Child health, nutrition	Treatment of childhood pneumonia, diarrhea, and malaria	Community based distributors	Qualitative interviews, focus group discussions, analysis of quantitative program data	− The large majority of CHWs continued their work during the crisis period.<br>− The number of children treated declined by about 50% at the peak of the crisis, but then quickly recovered. The number of children treated by CHWs was consistently higher than by health facilities, before, during, and after the crisis.<br>− Supervisors were able to use community networks to assess the security situation in specific areas and determine whether it was safe to travel and to track down CHWs to provide supervision and resupply them with drugs.<br>− CHWs often moved with communities when they were displaced and continued treating sick children. CHWs in areas that received IDPs treated both their original host population and IDPs. Displaced CHWs also treated their displaced community members and the host population.<br>− CHWs in areas that experienced a large influx of IDPs were not able to meet the demand for services due to limited drug supplies.<br>− Caregivers preferred to seek care in the community to avoid traveling in insecure areas and because of a perception that health facilities were closed or lacked essential medicines.
Kumar	Managing Child Malnutrition in a Drought Affected District of Rajasthan – A Case Study	2005	United Nations Children's Fund	Journal article	Indian Journal of Public Health	South-East Asia	India	Drought	General population	Child health, nutrition	Growth monitoring and promotion, treatment of childhood malnutrition, treatment of childhood diarrhea, immunization, vitamin A supplementation, nutrition and health education	Nutrition care centre workers, Anganwadi workers	Documentation of experience, pre-post surveys	− Local community members were quickly trained and deployed to manage childhood malnutrition and provide a number of other malnutrition-related services during a nutrition emergency.
Lantagne	Effective Use of Household Water Treatment and Safe Storage in Response to the 2010 Haiti Earthquake	2013	Tufts University, London School of Hygiene and Tropical Medicine	Journal article	American Society of Tropical Medicine and Hygiene	Americas	Haiti	Natural disaster	General population	WASH	Provision of water treatment and safe storage containers	Community health workers	Household surveys, household water testing	− CHWs distributed chlorine products and safe storage containers following the Haiti earthquake.

Lawton	Health care in Nicaragua	1988	World Health Organization	Journal article	Nurse Practitioner	Americas	Nicaragua	Conflict	General population	Child health, maternal health, WASH, infectious diseases, nutrition	Immunization, provision of malaria chemoprophylaxis, malaria and TB screening, nutrition screening, health education, WASH promotion, assisted home delivery, antenatal care, postnatal care	Brigadistas, traditional birth attendants	Documentation of experience	− Brigadistas provided a wide range of preventive, promotive, and curative health services in the community.<br>− TBAs assisted in home-deliveries when referral to a health facility was not possible.<br>− The healthcare system, which relied heavily on community-based health workers, led to high access to healthcare services and improved health indicators.<br>− War decreased availability of healthcare services, equipment and supplies for civilians.
Lee	Mortality rates in conflict zones in Karen, Karenni, and Mon states in eastern Burma	2006	University of California, Los Angeles; Bloomberg School of Public Health; Albert Einstein College of Medicine; University of California, Berkeley; Backpack Health Worker Team	Journal article	Tropical Medicine and International Health	South-East Asia	Myanmar	Conflict	General population	Research	Data collection for mortality survey	Backpack health workers	Household survey	− BPHWs members collected mortality data in areas affected by conflict that were inaccessible to outside data collectors.
Lee	Internally displaced human resources for health: Villager health worker partnerships to scale up a malaria control programme in active conflict areas of eastern Burma	2009	Global Health Access Program; Karen Department of Health and Welfare; University of California, San Francisco; University of California, Los Angeles	Journal article	Global Public Health	South-East Asia	Myanmar	Conflict	General population	Child health, infectious diseases	Treatment of malaria, health education	Village health workers	Documentation of experience	− Village health workers were trained, supervised, and supplied by mobile health workers who received training and supplies across the border in Thailand.<br>− Village-based health workers were less likely to be targeted by the Burmese military or militias than mobile health workers because of they are based in one community and were less conspicuous.
Lembani	A Case Study of Health Service Provision in Yobe State, Nigeria in the Context of the Boko Haram Insurgency	2014	University of Western Cape, Partnership for Reviving Routine Immunization in Northern Nigeria and Maternal Newborn Child Health Programme, Columbia University, University of Montreal	Report	N/A	Africa	Nigeria	Conflict	General population	Not specified	Not specified	Community health workers	Qualitative interviews	− CHWs coordinated with community members to warn health workers when it was not safe to travel to communities.
Lhussier	Development and implementation of a nutrition intervention programme in North West Pakistan: a realist framework	2011	Northumbria University, Nahaqi Emergency Satellite Hospital, University of Central Lancashire, Khyber Medical College	Journal article	Health Promotion International	Eastern Mediterranean	Pakistan	Conflict	General population	Maternal health, newborn health, nutrition	Cooking demonstrations, provision of nutrition supplementation, health and nutrition education	Lady health workers	Focus group discussions	− LHWs provided nutritional support and education in areas with high levels of malnutrition.<br>− The program had a positive impact on knowledge gained by women in the community.

Lim	Trauma and mental health of medics in eastern Myanmar's conflict zones: a cross-sectional and mixed methods investigation	2013	University of California Berkeley School of Public Health, University of California San Francisco School of Medicine, Global Health Access Program, University of California Los Angeles School of Medicine, Karen Department of Health and Welfare	Journal article	Conflict and Health	South-East Asia	Myanmar	Conflict	General population	Not specified	Basic healthcare (not specified), health education	Backpack health workers	Survey of BPHWs, qualitative interviews	− BPHWs discussed a variety of hardships resulting from their roles, including challenges related to their work, personal/family-related issues, security, violence, and threats from the Burmese military, and early life trauma.<br>− Work-related sources of distress resulted from feelings of incompetence, lack of medical resources, transportation barriers and personnel shortages.<br>− Medics who spoke of feelings of ineptness in patient care also expressed feelings of shame, helplessness, and embarrassment.<br>− The majority of BPHWs seldom saw their families for extended periods of time, ranging from once every three months to over a decade.<br>− BPHWs felt unprepared in cases of complex medical situations.<br>− Security issues and threats of violence from the Burmese army were a major stressor for the medics. These continuing security threats limited medics' ability to provide health services.<br>− The fear of being attacked while traveling was a significant source of anxiety.<br>− BPHWs were often at risk of injury from antipersonnel landmines, with many reportedly triggering or nearly detonating landmines.<br>− BPHWs felt that the relations developed within medic teams were essential for psychological wellbeing and social support, especially in isolated or unstable areas.<br>− Forms of coping included confiding in friends and family, group activities, personal hobbies, and religious activities.
Lim	Vicarious traumatization and resilience of health workers	2015	N/A	Book chapter	Dartmouth College Press	South-East Asia	Myanmar	Conflict	General population	Not specified	Not specified	Backpack health workers	Qualitative research (not specified)	− BPHWs experienced manifestations of psychiatric symptoms due to violence, a loss of security, and other stressors.<br>− BPHWs recounted traumatic events that left lasting impressions, such as being beaten by Burmese soldiers, witnessing family members abused or killed by soldiers, and seeing dead bodies in the aftermath of destroyed villages.<br>− BPHWs used emotion-focused coping mechanisms, centered on social engagement, peer support, and personal time, along with problem-focused coping, motivated by altruism in their community-oriented work.

Lin	Stimulating demand: An assessment of the conditional cash transfer project in Afghanistan	2016	Johns Hopkins University	Disserta tion	N/A	Eastern Mediterran ean	Afghanist an	Conflict	General population	Maternal health, child health	Referral for facility delivery, referral for immunization	Community health workers	Household survey, qualitative interviews, health facility assessments, document review	− Cash payments to CHWs for DPT3 and institutional delivery referrals were not associated with service utilization.<br>− The incentive program made it possible to recruit CHWs in communities that did not have CHWs previously.
Lori	Patient Satisfaction With Maternity Waiting Homes in Liberia: A Case Study During the Ebola Outbreak	2017	University of Michigan, Africare, Management Sciences for Health	Journal article	Journal of Midwifery & Women's Health	Africa	Liberia	Disease outbreak	General population	Maternal health, infectious diseases	Support to pregnant women at maternal waiting homes	Traditional birth attendants	Patient satisfaction surveys, qualitative interviews	− TBAs stayed with women during their time in maternal waiting homes and described a positive environment at MWHs.
Low	Human resources for health: task shifting to promote basic health service delivery among internally displaced people in ethnic health program service areas in eastern Burma/Myanmar	2014	Community Partners International, Burma Medical Association, Health Information System Working Group, Back Pack Health Worker Team, Mae Tao Clinic, Karen Department of Health and Welfare, London School of Hygiene and Tropical Medicine	Journal article	Global Health Action	South-East Asia	Myanmar	Conflict	General population	Maternal health, newborn health, child health, reproductive health	Assisted home delivery, antenatal care, postnatal care, provision of contraceptives, immunization, health education, deworming, vitamin A supplementation, WASH promotion	Community health workers, maternal health workers, traditional birth attendants, village health workers	Qualitative interviews, analysis of quantitative program data, record review	− Task shifting resulted in a broad range of services being delivered by CHWs, MHWs, and TBAs, including medical care, maternal and child health services, and community health education and prevention.
Lucchi	Moving from the ‘why’ to the ‘how’: reflections on humanitarian response in urban settings	2012	Médecins Sans Frontières	Journal article	Disasters	Various	Various	Conflict, disease outbreak, natural disaster	General population	Not specified	Community outreach (not specified)	Community health workers, community outreach workers	Documentation of experience, qualitative interviews, document review	− Community outreach and interaction with communities is key to the urban medical strategy.<br>− Community-based workers can play a key role in monitoring and measuring achievements.
Lucknow	Implementation research on community health workers’ provision of maternal and child health services in rural Liberia	2017	Dartmouth College, Last Mile Health, University of Oxford, Harvard University, University of Minnesota, Liberia Ministry of Health, University of Georgetown	Journal article	Bulletin of the World Health Organization	Africa	Liberia	Disease outbreak	General population	Maternal health, newborn health, child health	Treatment of childhood pneumonia, diarrhea, and malaria; malnutrition screening; health education; promotion of facility delivery; screening pregnant women and newborns for danger signs; promotion of exclusive breastfeeding	Community health workers	Pre-post household surveys	− During the Ebola outbreak CHWs were trained to carry out active surveillance in the communities.<br>− No touch iCCM was enacted to prevent transmission of Ebola between patients and CHWs.

Magidson	Adaptation of community health worker-delivered behavioral activation for torture survivors in Kurdistan, Iraq	2015	Massachusetts General Hospital, Harvard Medical School, University of Maryland, University of Sulaimani, Johns Hopkins Bloomberg School of Public Health, University of Massachusetts Medical School	Journal article	Global Mental Health	Eastern Mediterranean	Iraq	Conflict	General population	Mental health	Psychosocial support	Community health workers	Documentation of experience	− CHWs delivered behavioral activation treatment for depression in a conflict setting with poor resources in Kurdistan, Iraq.<br>− Of those that initiated treatment, 52% completed all 12 sessions.<br>− Training CHWs in behavioral activation treatment for depression was less complicated and time consuming than training a more comprehensive treatment package that included both behavioral and cognitive strategies.
Maheen	Rural Women’s Experience of Living and Giving Birth in Relief Camps in Pakistan	2017	Deakin University	Journal article	PLOS Currents Disasters	Eastern Mediterranean	Pakistan	Conflict	Camp	Maternal health	Assisted home delivery	Traditional birth attendants	Qualitative interviews	− Woman who could not afford to go to a hospital or did not have the means of transportation, were assisted by TBAs.<br>− None of the TBAs had received clean delivery kits.
Malaria Consortium	Addressing emergency nutritional needs in young children	Not specified	Malaria Consortium	Report	N/A	Africa	South Sudan	Conflict	General population	Child health, nutrition	Treatment of childhood pneumonia, diarrhea, malaria, and malnutrition; health education; community mobilization	Community nutrition workers	Not specified	− CHWs were able to add treatment of childhood malnutrition on the existing iCCM services in an insecure contexts.
Mayhew	Determinants of Skilled Birth Attendant Utilization in Afghanistan: A Cross-Sectional Study	2008	Vancouver Coastal Health, University of British Columbia, Johns Hopkins Bloomberg School of Public Health, Indian Institute of Health Management Research, Afghanistan Ministry of Public Health	Journal article	American Journal of Public Health	Eastern Mediterranean	Afghanistan	Conflict	General population	Maternal health, newborn health	Not specified	Community health workers, traditional birth attendants	Household surveys	− There was a limited number of female CHWs.<br>− Male CHWs were associated with lower levels of skilled birth attendance.
Mbaeyi	Strengthening Acute Flaccid Paralysis Surveillance Through the Village Polio Volunteers Program in Somalia	2018	US Centers for Disease Control and Prevention, World Health Organization	Journal article	Clinical Infectious Diseases	Eastern Mediterranean	Somalia	Disease outbreak	General population	Infectious diseases	Active case finding, community sensitization	Village polio volunteers	Analysis of quantitative program data, document review	− VPVs carried out active polio case finding and community sensitization for immunization campaigns.<br>− VPVs accounted for a high proportion of AFP cases reported in Somalia.<br>− VPVs were especially important in areas with security and access limitations.
Mbonye	Ebola Viral Hemorrhagic Disease Outbreak in West Africa- Lessons from Uganda	2014	Makerere University, Uganda Ministry of Health, World Health Organization	Journal article	African Health Sciences	Africa	Uganda	Disease outbreak	General population	Infectious diseases	Active case finding, contact tracing, health education	Village health teams	Documentation of experience, document review	− Village health teams were an important part of the disease surveillance system in Uganda, and carried out active case finding, contact tracing, health education.
McBride	In Pakistan's flood-devastated Sindh province, female health workers play key role	2010	United Nations Children’s Fund	Web blog	N/A	Eastern Mediterranean	Pakistan	Natural disaster	General population	Child health, WASH, reproductive health	Health education, treatment of childhood diarrhea	Lady health workers	Documentation of experience	− Following a flooding emergency, LHWs provided community education on prevention and treatment of diarrhea.

McMahon	"We and the nurses are now working with one voice": How community leaders and health committee members describe their role in Sierra Leone's Ebola response	2017	Heidelberg University, Johns Hopkins School of Public Health, The International Rescue Committee, Durham University, Njala University, Mercy Hospital Research Laboratory	Journal article	BMC Health Services Research	Africa	Sierra Leone	Disease outbreak	General population	Infectious diseases	Contact tracing, case finding, health education, encouraging care seeking, labor-related tasks	Community volunteers	Focus group discussions	− During the Ebola outbreak, community volunteers carried out contact tracing, case finding, screening for symptoms, notifying burial teams of deaths, health education, promotion of care seeking, and labor-related tasks.<br>− Some activities were coordinated and compensated through programs supported by NGOs or the government, while others sprang from volunteers' own initiative.<br>− Community volunteers served to transmit the concerns of the community members to health providers and to answer sensitive questions.<br>− Community volunteers were motivated through a sense of service to their community, a fear of Ebola, and financial compensation.<br>− Emotional and logistical barriers were discouraging for community volunteers and made their tasks more difficult to execute.
McPherson	Are Birth-Preparedness Programmes Effective? Results From a Field Trial in Siraha District, Nepal	2006	Save the Children	Journal article	Journal of Health, Population and Nutrition	South-East Asia	Nepal	Conflict	General population	Maternal health	Health education, birth preparedness promotion	Community health workers	Pre-post household surveys, no comparison	− CHWs provided health education and promoted birth preparedness for women in the community.<br>− Fifty-four percent of respondents (n=162) were directly exposed to BPP materials while pregnant.<br>− Women's knowledge, use of health services, preparation for emergencies, and key newborn practices all increased significantly.<br>− Skilled birth attendance and use of emergency obstetric care did not change.

Médecins Sans Frontières	Yambio HIV community-based test and treat pilot project	2018	Médecins Sans Frontières	Report	N/A	Africa	South Sudan	Conflict	General population	Infectious diseases	Health education, HIV testing, follow-up with patients, active HIV case finding	Community health workers	Documentation of experience	− CHWs carried out education on HIV, active case finding, patient follow-up, and in exceptional cases, HIV testing.<br>− The programme was successful in achieving higher ART coverage for those with HIV.<br>− Hiring CHWs from the area of operation improved the follow-up of patients to ensure adherence to treatment.<br>− In periods of active insecurity, CHWs provided patients with a 'runaway' bag containing three months of additional ARV as well as their regular stock. During the period of the project, the contingency plan was triggered several times. Evaluation of the context at regular intervals allowed the team to activate the contingency plan on time, before the situation deteriorated to a point where restrictions imposed on the movements of the CHWs would make them unable to pick up the medication from the clinic. Evaluation of the context at regular intervals allowed the team to activate the contingency plan on time, before the situation deteriorated to a point where restrictions imposed on the movements of the CHWs would make them unable to pick up the medication from the clinic.
Memon	Residual Barriers for Utilization of Maternal and Child Health Services: Community Perceptions From Rural Pakistan	2016	Aga Khan University	Journal article	Global Journal of Health Science	Eastern Mediterran ean	Pakistan	Conflict	General population	Maternal health, newborn health, child health	Health education	Lady health workers	Focus group discussions	− Home based deliveries were preferred unless complications occurred or TBAs were not competent enough.<br>− Awareness of the importance of key MNCH practices was low in areas with and without LHWs, although awareness of the importance of care seeking was higher in areas with LHWs.
Miller	Trained traditional birth attendants as educators of refugee mothers	1995	Mercy Corps International, Tufts University School of Medicine	Magazin e article	World Health Forum	Eastern Mediterran ean	Pakistan	Conflict	Camp	Maternal health, child health	Health education; promotion of immunization, nutrition, and hygiene; prenatal evaluation; postnatal visits	Traditional birth attendants	Survey of TBAs and households	− Trained birth attendants visited pregnant women frequently, advising on immunizations and hygiene and conducting prenatal evaluations, and performing postnatal visits.<br>− Trained birth attendants had better knowledge of disease prevention and breastfeeding than untrained birth attendants.<br>− Mothers reported that trained birth attendants used recommended sterilization procedures, while untrained birth attendants had unsafe practices during delivery and failed to follow appropriate postpartum procedures, and didn't consistently carry out postnatal visits.

Miller	Community health workers during the Ebola outbreak in Guinea, Liberia, and Sierra Leone	2018	United Nations Children's Fund, Anthrologica, Republic of Guinea Ministry of Health, Republic of Sierra Leone Ministry of Health and Sanitation, Republic of Liberia Ministry of Health	Journal article	Journal of global health	Africa	Guinea, Liberia, Sierra Leone	Disease outbreak	General population	Child health, maternal health, newborn health, infectious diseases	Treatment of childhood pneumonia, diarrhea, and malaria; health education; malnutrition screening; immunization; infectious disease control; postnatal visits; monitoring and referral of pregnant women	Community health workers, traditional birth attendants	Qualitative interviews, focus group discussions, analysis of quantitative program data	− CHWs and TBAs continued their work during Ebola, including in the early outbreak period when they did not receive any instruction or support.<br>− Case management services declined sharply (and ceased altogether in some areas) because of explicit instruction to discontinue treatments or because of lack of supplies of drugs. However, when CHWs received clear instructions to continue services and supplies, services quickly rebounded.<br>− Services that did not depend on drug supplies continued, demonstrating that CHWs continued to be active and continued providing their routine services that were not dependent on outside support or supplies.<br>− World Health Organization, United Nations Children's Fund, and MoHs developed "no touch" iCCM policy. However, CHWs received training on the "not touch" policy late in the outbreak and there was a great deal of confusion over the policy.<br>− Routine services were more likely to be disrupted in districts that were affected earlier in the outbreak. In districts that had later transmission, they were better prepared and policies and structures were already in place, CHWs could be trained on the "no touch" policy, and routine services were more likely to continue.<br>− In Guinea, Liberia, and Sierra Leone during Ebola, supply chain and supervision weaknesses that hampered service delivery before the outbreak were further exacerbated by the outbreak.<br>− Because of their ties to health facilities, communities displayed elevated levels of fear and mistrust toward CHWs. However, the close relationships CHWs had with community members were more resilient than the relationships communities had with facility-based health workers and community members preferred to seek care from CHWs than from health facilities. The trust of CHWs as community members also helped overcome confusion and fear about Ebola and many respondents believed CHWs' activities helped break chains of transmission.<br>− Women increased home deliveries with TBAs rather than go to health facilities. However, TBAs were rarely given any training, support, or protective materials.

Miller	Implementing integrated community case management during conflict in Yemen	2019	United Nations Children's Fund, Save the Children	Manuscript	N/A	Eastern Mediterranean	Yemen	Conflict	General population	Child health	Treatment of childhood pneumonia, diarrhea, and malaria; health education; malnutrition screening	Community health workers	Qualitative interviews, focus group discussions	− CHWs provided treatment for uncomplicated pneumonia, diarrhea, and malaria; provided education on health, nutrition, and hygiene; screened children for acute malnutrition.<br>− Policy, coordination, and funding were challenged by the fact that iCCM was not integrated into the national health system and was implemented as a short-term emergency program.<br>− Villages that received services from a CHW who was based in a different community experienced reduced access to services, especially during times of heightened conflict and insecurity, when CHWs could not travel.<br>− Supervision, supply chain, and monitoring were all challenges that were exacerbated by difficulties in travel due to the conflict. Potential solutions to these included the use of mobile technology for supervision and data collection and pre-positioning of buffer stocks in locations closer to CHWs.<br>− Travel was seen as the primary threat to the safety of CHWs and supervisors. Measures taken to reduce the risk included limiting travel during periods of heightened insecurity, safety training for CHWs, and use of mobile technology for communication.

Miller	Community health workers during the Ebola outbreak in Guinea	2017	Anthrologica, United Nations Children's Fund, Republic of Guinea Ministry of Health	Report	N/A	Africa	Guinea	Disease outbreak	General population	Child health, maternal health, newborn health, infectious diseases	Treatment of childhood pneumonia, diarrhea, and malaria; health education; malnutrition screening; immunization; infectious disease control; postnatal visits; monitoring and referral of pregnant women	Community health workers, traditional birth attendants	Qualitative interviews, focus group discussions, analysis of quantitative program data	− Whether case management services continued was dependent on several factors, including instructions from the NGO partner and whether an area experienced Ebola transmission earlier or later in the outbreak.<br>− When CHWs were instructed to continue and were trained on the 'no touch' protocol, many CHWs remained active in their communities and were willing to continue providing health-related services.<br>− Although CHWs faced mistrust and stigma because of their ties to health facilities, they were better able to gain the trust of community members because of their longstanding relationships.<br>− CHWs carried out contact tracing, case finding, social mobilisation and community engagement, and informal caregiving to sick community members.<br>− Engagement of trusted and respected community leaders was also crucial to mounting an effective community response to the emergency.<br>− TBAs played an important role in supporting maternal health and traditional healers gained increased prominence as trust in health workers diminished, often performing their duties without adequate infection protection.

Miller	Community health workers during the Ebola outbreak in Liberia	2017	Anthrologica, United Nations Children's Fund,	Report	N/A	Africa	Liberia	Disease outbreak	General population	Child health, maternal health, newborn health, infectious diseases	Treatment of childhood pneumonia, diarrhea, and malaria; health education; malnutrition screening; immunization; infectious disease control; postnatal visits; monitoring and referral of pregnant women	General community health volunteers, trained traditional midwives	Qualitative interviews, focus group discussions, analysis of quantitative program data	− During the Ebola outbreak, many community-based MNCH services in 'hard hit' or 'hotspot' communities were largely discontinued.<br>− Confusion over the intent and practical implementation of the 'no touch policy' was also highlighted by study participants as a key factor in the discontinuation of MNCH services, and many interpreted the policy as an instruction to cease iCCM entirely.<br>− During the height of the outbreak, TTMs were often the only source of care available to women in need of assistance during pregnancy or delivery, largely because health facilities were closed, health workers refused care for pregnant and delivering women, and women were frightened of seeking care from health workers. However, TTMs were not provided with materials to perform community-based deliveries during the outbreak, nor were they given materials for enhanced infection prevention control (IPC), despite their high-risk work.<br>− Caregivers concluded that despite their fear, they were more willing to seek care at health facilities on the recommendation of 'strong' gCHVs who they had known before the outbreak.<br>− Community members also became more willing to follow the health advice of gCHVs after they had experienced their neighbours dying in the community from Ebola.<br>− CHVs shifted into new roles to perform a wide variety of activities in the response including social mobilisation, contact tracing, active case finding and caretaking.<br>− Many respondents in the study confirmed that the role of CHVs during the Ebola response was central to building trust between communities and the responders, government and health system.<br>− Communities with a CHDC in place prior to the Ebola outbreak appeared to be more resilient, were better able to rapidly mobilize trusted local personnel, and were more likely to self-mobilize and organised community-led solutions.

Miller	Community health workers during the Ebola outbreak in Sierra Leone	2017	Anthrologica, United Nations Children's Fund,	Report	N/A	Africa	Sierra Leone	Disease outbreak	General population	Child health, maternal health, newborn health, infectious diseases	Treatment of childhood pneumonia, diarrhea, and malaria; health education; malnutrition screening; immunization; infectious disease control; postnatal visits; monitoring and referral of pregnant women	Community health workers, traditional birth attendants	Qualitative interviews, focus group discussions, analysis of quantitative program data	− Participants gave numerous reasons for the withdrawal or reduction of iCCM services, including CHWs' fear or lack of confidence in their ability to provide services safely without becoming infected; preoccupation with or reorientation towards Ebola-related activities; lack of support for the continuation of routine services; and because of a clear directive from an implementing partner or supervising health facility.<br>− Several months after the start of the outbreak, CHWs were trained on a revised 'no touch' iCCM protocol. The policy was widely supported by service providers, yet a number of district- and national-level stakeholders engaged in this study conceded that it had been introduced too late in the response and only after a number of CHWs had died after being infected by Ebola during the course of their work.<br>− Quantitative programme data from the four counties show that iCCM treatments and malnutrition screening declined from the early period of the outbreak in June 2014. However, services did continue at a reduced level. iCCM services started to recover around November 2014.<br>− TBAs played an important role during the Ebola outbreak, monitoring pregnant women in their communities and accompanying them to the health facility for delivery if possible.<br>− The long established close and trusting relationship between communities and their CHWs was significantly more resilient than the relationship communities had with facility-based health workers.<br>− CHWs were slow to be included in the formal response, and in the early phases of the response, there was no formal coordination of CHWs.<br>− Informally CHWs functioned as 'caregivers' in their communities bringing food and water to quarantined families, supporting the sick whilst awaiting the ambulance, and encouraging those who were afraid to attend health facilities. More formally, CHWs primarily contributed to the Surveillance Pillar as contact tracers and 'case finders' providing community events based surveillance. They also worked on social mobilisation and community engagement.

Minden	Midwives for refugees	1997	American Refugee Committee	Journal article	World Health	South-East Asia	Myanmar	Conflict	Camp	Maternal health	Treatment of common illnesses (not specified), provision of contraceptives, newborn growth monitoring, health education	Community health workers, MCH midwives	Documentation of experience	− Due to the high numbers of baby deaths and other conditions in which child bearing women suffered, female CHWs were selected to be a part of a special training in maternal health.
Miyake	Community midwifery initiatives in fragile and conflict-affected countries: a scoping review of approaches from recruitment to retention	2017	London School of Hygiene and Tropical Medicine, Jhpiego	Journal article	Health Policy and Planning	Various	Various	Conflict	General population	Maternal health	Assisted home delivery, health education	Community midwives	Literature review	− Community nomination of candidates was credited with facilitating community acceptance.<br>− As the CMW cadre worked closely with communities, CMWs provided services beyond midwifery, such as health promotion, child health and broader reproductive health issues.<br>− Common challenges to training and implementation included lacking funding, trainers, equipment, and other materials.<br>− Insecurity caused challenges in recruitment, retention, the ability of CMWs to travel to households, and supervision.<br>− Reasons for leaving the CMW position included family opposition to deployment, overwhelming responsibilities beyond midwifery duties, discrimination, lack of incentives, spouse relocation, and seeking better paid/urban employment.<br>− CMW candidate eligibility was problematic because of women's limited access to education.<br>− Receiving education and an independent income was noted as contributing to women's empowerment and improved social status.

Mullany	Impact of Community-Based Maternal Health Workers on Coverage of Essential Maternal Health Interventions among Internally Displaced Communities in Eastern Burma: The MOM Project	2010	Johns Hopkins Center for Public Health and Human Rights, UCLA School of Medicine, Global Health Access Program, Burma Medical Association, Karen Department of Health and Welfare, Mae Tao Clinic	Journal article	PLOS Medicine	South-East Asia	Myanmar	Conflict	General population	Maternal health	Emergency obstetric care, blood transfusion, antenatal care, postnatal care, counseling and provision of family planning	Maternal health workers, lay health workers, traditional birth attendants	Pre-post household surveys, no comparison	− Non-resident MHWs provided essential maternal health interventions including emergency obstetric care.<br>− MHWs provided five of the six components of basic emergency obstetric care, plus blood transfusion.<br>− MHWs trained and worked with less skilled lay health workers and TBAs, who were closer to the community than MHWs and provided less technical services.<br>− MHWs were able to provide, at the community level, services normally considered feasible only in facility-based settings.<br>− Women mostly delivered at home (82.9%), or at one of the mobile locations (12.0%) set up by the MHWs and hospital delivery was rare (4.2%).<br>− 44% of women reported that their delivery was attended by a MHW.<br>− Attendance at birth by those trained to deliver elements of emergency obstetric care, antenatal care, postnatal care, use of modern contraceptives, urine testing, malaria screening, and deworming all increased substantially and significantly between baseline and endline.<br>− Security-related obstacles prevented MHWs from reaching a higher number of deliveries.

Mullany	The MOM Project: Delivering Maternal Health Services among Internally Displaced Populations in Eastern Burma	2008	Johns Hopkins Center for Public Health and Human Rights, Global Health Access Program, Karen Department of Health and Welfare, Mae Tao Clinic	Journal article	Reproductive Health Matters	South-East Asia	Myanmar	Conflict	General population	Maternal health	Emergency obstetric care, blood transfusion, antenatal care, postnatal care, counseling and provision of family planning	Maternal health workers, lay health workers, traditional birth attendants	Documentation of experience	− Non-resident MHWs provided essential maternal health interventions including emergency obstetric care.<br>− MHWs provided five of the six components of basic emergency obstetric care, plus blood transfusion.<br>− MHWs trained and worked with less skilled lay health workers and TBAs, who were closer to the community than MHWs and provided less technical services.<br>− MHWs were able to provide, at the community level, services normally considered feasible only in facility-based settings.<br>− MHWs were trained in Thailand and then trained the lay health workers and TBAs in the communities.<br>− Supervision of the lay health workers and TBAs was carried out by the MHWs. MHWs received supervision from team leaders in Myanmar and during annual follow-up trainings in Thailand.<br>− Using local data collectors for data collection for monitoring and evaluation activities was crucial for access and community acceptance.<br>− Military conflict and intervention hindered coordination of activities and supplies, along with training of MHWs.<br>− However, some continuity was maintained because local health workers moved with displaced populations and continued providing services.
Muneghina	From individual to collective healing	2014	War Trauma Foundation	Magazine article	Therapy Today	Eastern Mediterranean	Sudan	Conflict	General population	Mental health	Psychosocial support	Community-based social workers	Documentation of experience	− Community-based social workers were trained to provide psychosocial support in communities through narrative theater and individual psychosocial counseling.
Murad	Prehospital trauma care reduces mortality. Ten year results from a time-cohort and trauma audit study in Iraq	2012	Trauma Care Foundation Iraq, University of Tromso, Norwegian School of Veterinary Science, University Hospital North Norway	Journal article	Scandinavian Journal of Trauma, Resuscitation and Emergency Medicine	Eastern Mediterranean	Iraq	Conflict	General population	Trauma	Trauma care	Lay trauma first responders	Cohort study	− Lay trauma first responders were trained to provide basic life support measures for trauma.<br>− Improved response time because of lay trauma first responders may have contributed to improved survival.
Murad	Trained Lay First Responders Reduce Trauma Mortality: A Controlled Study of Rural Trauma in Iraq	2010	Trauma Care Foundation Iraq, Suleimaniah Institute of Clinical Medicine, University of Tromsoe, University Hospital North Norway	Journal article	Prehospital and Disaster Medicine	Eastern Mediterranean	Iraq	Conflict	General population	Trauma	Trauma care	Lay trauma first responders	Quasi-experimental study	− Lay trauma first responders were trained to provide basic life support measures for trauma.<br>− The mortality rate was significantly lower among patients initially managed in-field by first responders compared to patients without first-responder support.<br>− Trained layperson first responders improve trauma outcomes where prehospltal evacuation times are long.

Nahimana	Knowledge, attitude and practice of hygiene and sanitation in a Burundian refugee camp: implications for control of a Salmonella typhi outbreak	2017	World Health Organization, Rwanda Biomedical Center, University of Rwanda School of Public Health, UNHCR	Journal article	Pan African Medical Journal	Africa	Rwanda	Disease outbreak	Camp	Infectious diseases	Hygiene education	Community health workers, community hygiene promoters	Household survey	− CHWs and community hygiene promoters poor knowledge and skills because of inadequate training, which contributed to poor knowledge about disease prevention among the refugees and to disease outbreaks.
Najafizada	Community health workers of Afghanistan: a qualitative study of a national program	2014	University of Ottowa	Journal article	Conflict and Health	Eastern Mediterranean	Afghanistan	Conflict	General population	Maternal health, newborn health, child health, reproductive health, nutrition, WASH	Health education; immunization; treatment of pneumonia, diarrhea, and anemia; promotion of skilled birth attendance; postnatal visits; distribution of contraceptives; first aid; vitamin A supplementation	Community health workers	Document analysis, qualitative interviews, focus group discussions, non-participant observation	− Different roles for male and female CHWs were not delineated in national policy, but in practice male and female CHWs tended to carried out different tasks, with women focused more on maternal and child health.<br>− Being a CHWs empowered women by increasing their social status and allowing them greater mobility. However, few women were CHW supervisors or in managerial or policy-making positions.<br>− Drug shortages were a major barrier to service provision and stockouts led to a lack of trust in CHW services. Areas supported by NGOs were less likely to experience stockouts.<br>− Community involvement in the community health program, through village health councils, were an important facilitator that allowed CHWs to work effectively.
Nawaz	The role of Community Health Workers in the heart of crisis	2017	FHI360	Web blog	N/A	Africa	Nigeria	Conflict	General population, camp	Child health, maternal health	Treatment of minor ailments (not specified), referral for antenatal care, labor and delivery and family planning services (not specified), health education, hygiene promotion, disease surveillance, distribution of mosquito nets	Community health extension workers, environmental health assistant volunteers	Documentation of experience	− Community health extension workers and environmental health assistant volunteers were trained to treat minor ailments; refer women for antenatal care, labor and delivery, or family planning services; provide health education; conduct hygiene promotion; carry out disease surveillance; and distribute mosquito nets in communities and IDP camps.

Naziri	Scaling up newborn care in Afghanistan: opportunities and challenges for the health sector	2018	United Nations Children's Fund Afghanistan, Afghanistan Ministry of Public Health, U.S Agency for International Development	Journal article	Health Policy and Planning	Eastern Mediterranean	Afghanistan	Conflict	General population	Maternal health, newborn health, child health, reproductive health, nutrition, WASH	Health education; immunization; treatment of pneumonia, diarrhea, and anemia; promotion of skilled birth attendance; postnatal visits; distribution of contraceptives; first aid; vitamin A supplementation	Community health workers	Literature review, analysis of survey data	− CHWs in Afghanistan provided basic health education and simple medical treatment since being trained and deployed in Afghanistan.<br>− Female CHWs accomplished MNCH tasks more easily than male CHWs.<br>− Insecurity and traditional attitudes to gender in Afghanistan make it difficult to attract and retain female health workers.<br>− Community participation facilitated the tasks of CHWs, but also posed challenges, such as traditional leaders influencing the recruitment of CHWs who may not be the most appropriate candidate.
Newbrander	Compliance with referral of sick children: a survey in five districts of Afghanistan	2012	Management Sciences for Health, Lawndale Christian Health Center, United Nations Children's Fund	Journal article	BMC Pediatrics	Eastern Mediterranean	Afghanistan	Conflict	General population	Child health	Assessment, management, and referral of sick children (not specified)	Community health workers	Household survey	− The majority of children brought to a CHW were referred to another healthcare provider, which may indicate a lack of confidence to treat uncomplicated illnesses.<br>− Caretakers were more likely to comply with referral recommendations from community members (relative, friend, CHW, traditional healer) than with recommendations from health workers.
O'Brien	Provision of antiretroviral treatment in conflict settings: the experience of Médecins Sans Frontières	2010	Médecins Sans Frontières, Geelong Hospital, Royal Melbourne Hospital, Red Cross Children's' Hospital, University of Auckland	Journal article	Conflict and Health	Not specified	Not specified	Conflict	General population, camp	Infectious diseases	HIV counseling and adherence monitoring	Community health workers	Documentation of experience	− CHWs were trained to provide HIV counseling and adherence monitoring.
O'Heir	Pregnancy and childbirth care following conflict and displacement: care for refugee women in low-resource settings	2004	N/A	Journal article	Journal of Midwifery & Women's Health	Africa	Kenya, Tanzania	Conflict	General population, camp	Maternal health	Antenatal care, assisted home delivery	Traditional birth attendants	Documentation of experience	− In refugee settings in Tanzania and Kenya, the majority of deliveries are attended by trained TBAs.
Ohly	Developing health service delivery in a poor and marginalised community in North West Pakistan	2018	Abaseen Foundation, Institute of Public Health, Lady Reading Hospital, University of Central Lancashire, Khyber Medical University	Journal article	Pakistan Journal of Medical Sciences	Eastern Mediterranean	Pakistan	Conflict	General population	Maternal health, child health, reproductive health	Health education, referral for ANC	Community health workers, traditional birth attendants	Documentation of experience, pre-post household surveys, analysis of routine quantitative data, focus group discussions	− Volunteer staff promoted health awareness and health education within the community.<br>− Trust was developed through engagement with village health committees and heads of households, which enabled pregnant women to attend the health center.

Oladele	An assessment of the emergency response among health workers involved in the 2010 cholera outbreak in northern Nigeria	2012	Nigerian Institute of Medical Research, Nigeria Federal Ministry of Health	Journal article	Journal of Infection and Public Health	Africa	Nigeria	Disease outbreak	General population	Infectious diseases	Referral to health facilities	Community health extension workers	Health worker survey	− Healthcare workers (most of whom were CHWs) reported lack of funding, resources, supplies, and training hindering them from patient case management.<br>− Most of the healthcare workers involved in case management are either poorly trained or not trained at all.<br>− Cases of cholera infection were reported in healthcare workers during the outbreak.
Ongwae	Use of Dedicated Mobile Teams and Polio Volunteer Community Mobilizers to Increase Access to Zero-Dose Oral Poliovirus Vaccine and Routine Childhood Immunizations in Settlements at High Risk for Polio Transmission in Northern Nigeria	2017	United Nations Children's Fund, World Health Organization, Nigeria Federal Ministry of Health,	Journal article	Journal of Infectious Diseases	Africa	Nigeria	Conflict	General population	Child health	Conduct household microcensus, home visits, immunization, tracking and linking of newborns with facility	Volunteer community mobilizers	Document review, pre-post household surveys	− To address gaps in polio immunization coverage, mobile health teams and a network of volunteer community mobilizers were used to target settlements at high risk for polio transmission in northern Nigeria.<br>− Volunteer community mobilizers conducted household microcensus, home visits, administration of oral polio vaccine, tracking and linking of newborns with routine services in the nearby health facilities.<br>− The proportion of children fully immunized increased from 19% to 55% and the number of children receiving polio vaccine increased.<br>− Community engagement involving traditional and religious leaders reduced resistance to the poliovirus vaccine and mobilized communities to utilize the services.
Orach	Maternal mortality estimated using the Sisterhood method in Gulu district, Uganda	2000	Makerere University	Journal article	Tropical Doctor	Africa	Uganda	Conflict	General population	Maternal health	Assisted home delivery	Traditional birth attendants	Household survey	− About half of deliveries were attended by untrained TBAs and only a few deliveries were attended by trained TBAs.<br>− A significant number of maternal deaths occurred at the homes of untrained TBAs. No deaths occurred at the homes of trained TBAs.<br>− Training and support to TBAs can avert deaths in conflict settings.
Pacichana-Quinayáz	Common Elements Treatment Approach based on a Cognitive Behavioral Intervention: implementation in the Colombian Pacific	2016	Universidad del Valle	Journal article	Ciência & Saúde Coletiva	Americas	Colombia	Conflict	General population	Mental health	Psychosocial support	Lay psychosocial community workers	Qualitative interviews	− LPCW were trained to provide a Common Elements Treatment Approach psychosocial support intervention for people displaced by conflict.<br>− After implementation of CETA by LPCWs, victims reported improvements in their symptoms, including less anxiety and better feelings about the future.<br>− Training LPCWs on mental health issues had positive findings due to low cost, greater availability, and adequate effectiveness in assisting and accompanying people with mental disorders.

Parmar	Health and Human Rights in Eastern Myanmar after the Political Transition: A Population-Based Assessment Using Multistaged Household Cluster Sampling	2015	Brigham and Women's Hospital, Harvard Medical School, Community Partners International, University of Washington, Burma Medical Association, Health Information Systems Information Group, Back Pack Health Worker Team, Mae Tao Clinic, Karen Department of Health and Welfare, Karenni Mobile Health Committee, Mon National Health Committee, Harvard School of Public Health, University of California Los Angeles	Journal article	PLOS One	South-East Asia	Myanmar	Conflict	General population	Maternal health, reproductive health	Antenatal care, assisted home delivery	Traditional birth attendants	Household survey	− Ethnic minority TBAs addressed large delivery gaps and providing reproductive health to women in Myanmar.
Patel	Ebola Outbreak in Nigeria: Increasing Ebola Knowledge of Volunteer Health Advisors	2016	Healthy Sunrise Foundation, Global Solutions for Prevention, Education, Treatment, Training and Research, University of Nevada School of Medicine, University of Nevada, Las Vegas, Association of Nigeria Physicians in the Americas	Journal article	Global Journal of Health Science	Africa	Nigeria	Disease outbreak	General population	Infectious diseases	Health education	Volunteer health advisors	Pre-post knowledge tests	− VHAs' knowledge of Ebola improved after they received training.

Perry	Community health worker programmes after the 2013–2016 Ebola outbreak	2016	Johns Hopkins Bloomberg School of Public Health, Brigham and Women's Hospital, Harvard Medical School, Columbia University, United Nations Children's Fund, Last Mile Health, Partners In Health, Sierra Leone Ministry of Health and Sanitation, Guinea Ministry of Health and Hygiene, Liberia Ministry of Health and Social Welfare	Journal article	Bulletin of the World Health Organization	Africa	Guinea, Liberia, Sierra Leone, Nigeria	Disease outbreak	General population	Infectious diseases	Case detection, contact tracing, health education, community sensitization, promotion of protective practices, and data collection	Community health workers	Documentation of experience	− During the West Africa Ebola outbreak, CHWs carried out case detection, contact tracing, health education, community sensitization, promotion of protective practices, and data collection.<br>− In Nigeria, CHWs who were normally engaged in polio eradication initiatives were rapidly redeployed to detect patients with Ebola virus and trace their contacts.<br>− CHWs can help limit the spread of future outbreaks through early detection and rapid containment of cases. Community health workers are particularly important in remote villages or hard-to-reach populations, where people often live far from the nearest health facility. The identification of suspected cases by a community health worker can serve as an early warning system for disease outbreaks.<br>− CHWs can play an important role in controlling outbreaks by engaging and educating communities.
Pett	Our community health workers are the MSF project	2017	Médecins Sans Frontières	Web blog	N/A	Africa	South Sudan	Conflict	General population	Child health	Treatment of childhood diarrhea, malaria, and skin diseases	Community health workers	Documentation of experience	− When communities are displaced because of fighting, CHWs move with them and continue providing services.
Plucinski	Effect of the Ebola-virus-disease epidemic on malaria case management in Guinea, 2014: a cross-sectional survey of health facilities	2015	Centers for Disease Control and Prevention, President's Malaria Initiative, Guinea Ministry of Health, Mafèrinyah Rural Health Research Center, Catholic Relief Services, Emory University	Journal article	Lancet Infectious Diseases	Africa	Guinea	Disease outbreak	General population	Child health	Treatment of malaria	Community health workers	Health facility survey, analysis of surveillance data, qualitative interviews	− The number of patients treated for malaria during the Ebola outbreak decreased by 24% compared to the previous year. Overall HF attendance decreased by 11%.<br>− The % of CHWs in Ebola-affected areas reported being active fell from 98% before the outbreak to 74% during the outbreak.<br>− The % of CHWs doing malaria case management fell from 68% to 48%. Only 30% reported using RDTs, down from 70% before the outbreak.
Plucinski	Ebola epidemic impedes malaria care delivery in Guinea	2015	Centers for Disease Control and Prevention, Guinea Ministry of Health, Mafèrinyah Rural Health Research Center, Catholic Relief Services, Emory University	Confere nce abstract	American Society of Tropical Medicine and Hygiene	Africa	Guinea	Disease outbreak	General population	Child health	Treatment of malaria	Community health workers	Health facility survey, analysis of surveillance data, qualitative interviews	− In Ebola-affected areas of Guinea, the number of patients treated with oral and injectable antimalarials during the Ebola outbreak decreased by 58% and 69%, respectively, compared to the previous year.<br>− Overall health facility attendance decreased by 42% in Ebola-affected areas.<br>− In Ebola-affected prefectures, only 74% of community health workers were operational, and only 48% of those working reported actively treating malaria cases.

Plummer	Community health for Rwandan refugees	1995	Not specified	Journal article	The Canadian Nurse	Africa	Rwanda	Conflict, disease outbreak	Camp	Infectious diseases	Active case finding, distribution of ORS, health education	Community health workers	Documentation of experience	− Community Health Workers (CHW), were trained in active case finding for patients with major illnesses and distributed ORS to patients suffering from dehydration.<br>− During a cholera outbreak, CHWs carried out active case finding and conducted a mass education campaign, which was seen as key to reducing mortality.
Purdin	Surveillance beyond Camp Settings in Humanitarian Emergencies: Findings from the Humanitarian Health Information Management Working Group	2009	International Rescue Committee, United Nations High Commissioner for Refugees, Harvard School of Public Health, Brigham and Women's Hospital, Massachusetts General Hospital	Journal article	Prehospital and Disaster Medicine	Various	Various	Various	General population	Infectious diseases	Community-based surveillance	Various	Documentation of expert meeting	− Community volunteers/CHWs have been used to collect data on births, deaths, pregnancies, immunizations, and latrine use.<br>− The needs and resources required for community-based surveillance may be different for rural vs. urban, low-income vs. middle-income, and stable vs. unstable settings.
Rabadi	A community health worker approach: Timed and targeted counseling improves caregiver knowledge and behaviors related to child nutrition and management of illness in emergency-affected areas of West Bank, Palestine	Not specif ied	World Vision	Web blog	N/A	Eastern Mediterran ean	Palestine	Conflict	General population	Maternal health, newborn health, child health, nutrition	Behavior change communication, antenatal visits, postnatal visits	Community health workers	Pre-post household surveys	− CHWs in emergency-affected areas of Palestine conducted household visits during and after pregnancy to delivery behavior change messages related to maternal and child health and nutrition.<br>− Exclusive breastfeeding, vitamin D supplementation, correct management of diarrhea, and supplementation with iron/folate during pregnancy all increased significantly.
Rahman	Effect of a Multicomponent Behavioral Intervention in Adults Impaired by Psychological Distress in a Conflict-Affected Area of Pakistan	2016	University of Liverpool, Human Development Research Foundation, Lady Reading Hospital, University of New South Wales, VU University Amsterdam, Liverpool School of Tropical Medicine, Keele University, World Health Organization	Journal article	Journal of the American Medical Association	Eastern Mediterran ean	Pakistan	Conflict	General population	Mental health	Psychosocial support	Lay health workers	Randomized controlled trial	− Lay health workers were trained to provide psychosocial support to people experiencing psychological distress.<br>− After 3 months of treatment, the intervention group had significantly lower scores than the control group for anxiety, depression, posttraumatic, functional impairment, problems for which the person sought help, and symptoms of depressive disorder.
Raoot	Measles Outbreak in High Risk Areas of Delhi: Epidemiological Investigation and Laboratory Confirmation	2015	Government of NCT Delhi, Maulana Azad Medical College	Journal article	Indian Journal of Pediatrics	South-East Asia	India	Disease outbreak	General population	Infectious diseases	Active case finding and containment	Accredited social health activists, Anganwadi workers	Documentation of experience, analysis of surveillance data	− CHWs were part of field health teams conducting active case finding and containment of cases during a measles outbreak.

Ratnayake	Assessment of Community Event–Based Surveillance for Ebola Virus Disease, Sierra Leone, 2015	2016	International Rescue Committee, Centers for Disease Control and Prevention, Action contre la Faim, Sierra Leone Ministry of Health and Sanitation	Journal article	Emerging Infectious Diseases	Africa	Sierra Leone	Disease outbreak	General population	Infectious diseases	Disease surveillance	Community health monitors	Analysis of surveillance data	− Volunteer community health monitors were trained to detect 6 trigger events suggestive of Ebola virus transmission and report them to supervisors.<br>− The CEBS system had relatively low sensitivity and low positive predictive value. However, it did detect a large number of deaths in the community as well as measles outbreaks.
Richters	Sexual transgression and social disconnection: healing through community-based sociotherapy in Rwanda	2013	Leiden University Medical Center, Amsterdam Institute for Social Science Research, Institute of Higher Education in Mental Health	Journal article	Culture, Health and Sexuality	Africa	Rwanda	Conflict	General population	Mental health	Facilitation of sociotherapy group sessions	Group facilitators, community volunteers	Qualitative interviews, focus group discussions, participant observation, documentation of group sessions	− Group facilitators, which include community volunteers, lead group sociotherapy sessions with victims of genocide and rape.
Rodger	DOTS-based tuberculosis treatment and control during civil conflict and an HIV epidemic, Churachandpur District, India	2002	Burnet Institute for Medical Research and Public Health, Society for HIV/AIDS Lifeline Operation in Manipur	Journal article	Bulletin of the World Health Organization	South-East Asia	India	Conflict	General population	Infectious diseases	DOTS for TB	Community outreach workers	Analysis of quantitative program data	− Community outreach workers treated patients with TB in a conflict setting.<br>− There was a successful outcome of treatment in 89% of patients.<br>− The use of outreach workers from each ethnic group was important in facilitating free access to all areas and patients.<br>− Recognition by local leaders that TB was a major problem in their communities was also key to success of the program.
Rosales	Role of an international non-governmental organization in strengthening health systems in fragile-state context: Evaluation results from South Sudan	2015	World Vision	Journal article	African Evaluation Journal	Africa	South Sudan	Conflict	General population	Maternal health, newborn health, child health	Treatment of childhood pneumonia, diarrhea, and malaria; home birth assistance; newborn care; health education; community mobilization	Home health promoters	Pre-post household surveys, qualitative interviews, focus group discussions, document analysis	− In South Sudan, an intervention delivered by CHWs improved key maternal and child health indicators in a fragile context.<br>− The weakness of the government supply chain during the conflict forced the NGO to create a parallel supply chain.
Rosenberg	Let's talk about sex work in humanitarian settings: piloting a rights-based approach to working with refugee women selling sex in Kampala	2017	AIDS Information Center of Uganda	Journal article	Reproductive Health Matters	Africa	Uganda	Conflict	General population	Sexual and reproductive health	Health education	Peer educators	Qualitative interviews, focus group discussions	− Refugee sex workers were trained as peer educators to provide education on sexual and reproductive health and GBV.

Ruckstuhl	Malaria case management by community health workers in the Central African Republic from 2009–2014: overcoming challenges of access and instability due to conflict	2017	Swiss Tropical and Public Health Institute, University of Basel, Central African Republic National Malaria Control Programme, The MENTOR Initiative	Journal article	Malaria journal	Africa	Central African Republic	Conflict	General population	Maternal health, child health	Treatment of malaria, diarrhea, and helminth infection; health education; malnutrition screening and referral; vitamin A supplementation; provision of home delivery kits; bednet distribution	Community health workers	Analysis of quantitative program data	− CHWs treated large numbers of children and pregnant women in an area of periodic conflict and continued instability.<br>− Register review showed that CHWs overwhelmingly provided appropriate treatment for malaria and unnecessary antimalarial treatment was minimal.<br>− The CHW reporting rate fell from 95% to 70% during the peak of the conflict.<br>− Supervisors were able to continue supervision and consistent drug supplies through periods of heightened conflict and insecurity.<br>− CHWs were given extra buffer stock during the periods of increased insecurity.<br>− However, supervision and other support to CHWs did decrease during the height of the crisis because expat staff were evacuated from the country and access to program funds was limited.
Sabo	Political violence and Eritrean health care	1989	University of Missouri-Columbia	Journal article	Social Science and Medicine	Africa	Eritrea	Conflict	General population	Not specified	Basic healthcare (not specified), health education	Village health workers, traditional midwives	Documentation of experience	− Village health workers and traditional midwives were trained to provide healthcare services in communities.
Sadler	A comparison of the programme coverage of two therapeutic feeding interventions implemented in neighbouring districts of Malawi	2007	Valid International, Institute of Child Health, University College London	Journal article	Public Health Nutrition	Africa	Malawi	Nutrition emergency	General population	Nutrition	Malnutrition screening and referral	Community health workers	Household surveys in intervention and comparison areas	− CHWs were used for malnutrition screening and referral during a nutrition emergency.<br>− Coverage of therapeutic feeding was substantially higher in the community-based program that included CHWs than in the facility-based program.<br>− The community-based program also had more even coverage across the district, suggesting that the community-based model improved equity in access to services.
Sami	"You have to take action": changing knowledge and attitudes towards newborn care practices during crisis in South Sudan	2017	Johns Hopkins Bloomberg School of Public Health, Save the Children, US Centers for Disease Control and Prevention, United Nations Children's Fund, University of the Western Cape, South Sudan Ministry of Health, International Medical Corps	Journal article	Reproductive Health Matters	Africa	South Sudan	Conflict	Camp	Newborn health	Newborn care, promotion of ANC, assisted home delivery	Community health workers	Pre-post knowledge tests, qualitative interviews	− After receiving training, CHWs displayed higher levels of knowledge of newborn care practices and danger signs.<br>− A challenge was social stigma related to disclosing pregnancy. CHWs expressed that establishing trust and educating women of reproductive age regarding benefits of ANC would result in early identification.<br>− CHWs cited lack of staffing and prioritization as the main barriers to conducting timely home visits, particularly with competing activities such as vaccination campaigns or disease surveillance.

Sami	Understanding health systems to improve community and facility level newborn care among displaced populations in South Sudan: a mixed methods case study	2018	Johns Hopkins Bloomberg School of Public Health, Save the Children, South Sudan Ministry of Health, United Nations Children's Fund, University of the Western Cape, International Medical Corps, US Centers for Disease Control and Prevention	Journal article	BMC Pregnancy and Children	Africa	South Sudan	Conflict, disease outbreak	Camp	Newborn health	Newborn care, promotion of ANC and facility delivery	Community health workers	Qualitative interviews, focus group discussions, observation of health facilities	− CHWs demonstrated motivation to implement newborn interventions. However, the failure to prioritize newborn activities among program supervisors was described as a key challenge.<br>− CHWs were unable to reach all newborns for a postnatal home visit within the first 48h after birth because of competing priorities.<br>− CHWs stated that a wide scope of work and few workers to cover the large populations were factors that limited time spent on newborn care.<br>− A cholera outbreak and increase in GBV cases further increased CHWs' workloads, making it more difficult to carry out newborn care activities.
Schaider	International Maternal Mortality Reduction: Outcome of Traditional Birth Attendant Education and Intervention in Angola	1999	Cook County Hospital, International Medical Corps	Journal article	Journal of Medical Systems	Africa	Angola	Conflict	General population	Maternal health	Antenatal care, postnatal care, assisted home delivery, immunization, registration of births and fetal and maternal deaths, health education	Traditional birth attendants	Analysis of quantitative program data	− TBAs were trained to provide antenatal, delivery, and postnatal care.
Seddiq	Implementing a successful tuberculosis programme within primary care services in a conflict area using the stop TB strategy: Afghanistan case study	2014	Afghanistan National TB Control Programme, International Union against Tuberculosis and Lung Disease, Stop-TB, Pakistan Health Services Academy, World Health Organization Regional Office for the Eastern Mediterranean	Journal article	Conflict and Health	Eastern Mediterran ean	Afghanist an	Conflict	General population	Infectious diseases	Treatment of TB	Community health workers	Documentation of experience, literature review, qualitative interviews, analysis of quantitative program data	− CHWs were trained to provide directly-observed treatment for TB and treatment follow-up.
Sethi	Community-Based Noncommunicable Disease Care for Syrian Refugees in Lebanon	2017	Medical Teams International	Journal article	Global Health Science and Practice	Eastern Mediterran ean	Lebanon	Conflict	General population, camp	Non-communicabl e diseases	Monitoring of chronic disease markers and treatment adherence, health education	Refugee outreach volunteers	Documentation of experience	− Refugee outreach volunteers monitored patients with chronic diseases, provided referrals, and conducted health education in informal refugee settlements.

Shah	Approaches to support continued iCCM implementation during a flooding emergency in rural Bangladesh	2019	Save the Children, United Nations Children's Fund	Journal article	Journal of Global Health	South-East Asia	Banglades h	Natural disaster	General population	Child health	Treatment of childhood pneumonia, diarrhea, and malaria	Community health care providers, village doctors	Qualitative interviews, focus group discussions, analysis of quantitative program data	− CHWs continued providing services during the flooding emergency, although they had a decrease in the number of children treated.<br>− CHWs traveled to reach households and set up temporary clinics.<br>− Community clinics were flooded or were inaccessible, so CHWs visited households or set up temporary clinics in accessible areas.<br>− Private village doctors, who acquire their own drug stocks, planned for the floods by buying extra drug stocks and identifying safe storage. Community health care providers, who are government workers and rely on the government supply chain, could not prepare as effectively.<br>− Supervisors had difficulty reaching CHWs during the floods, so they communicated with CHWs by phone.
Shriram	Community-centred approach for the control of Aedes spp. in a peri-urban zone in the Andaman and Nicobar Islands using temephos	2009	Indian Council of Medical Research	Journal article	The National Medical Journal of India	South-East Asia	India	Disease outbreak	General population	Infectious diseases	Household container water treatment, health education	Community volunteers	Pre-post entomological surveys	− Community volunteers disseminated information to the residents on the Chikungunya outbreak.<br>− Community volunteers were responsible for applying Temephos and calculating the surface area of breeding sources.<br>− After the intervention, the Breteau and house indices dropped from 104.8% to 2.7% and 44.23% to 2.6%, respectively.
Shukla	Impact of a Health Governance Intervention on Provincial Health System Performance in Afghanistan: A Quasi-Experimental Study	2018	Management Sciences for Health	Journal article	Health Systems and Reform	Eastern Mediterran ean	Afghanist an	Conflict	General population	Not specified	Home visits (not specified)	Community health workers	Quasi-experimental study using routine data	− The health governance intervention had a negative impact on community health worker home visits.

Siekmans	Community-based health care is an essential component of a resilient health system: evidence from Ebola outbreak in Liberia	2017	HealthBridge, Canadian Red Cross Society, Liberian Ministry of Health and Social Welfare, Liberia Red Cross Society	Journal article	BMC Public Health	Africa	Liberia	Disease outbreak	General population	Child health	Treatment of childhood pneumonia and diarrhea	General community health volunteers	Survey of CHWs, qualitative interviews, focus group discussions, analysis of quantitative program data	− During Ebola, most community members were reluctant or refused to go to HFs for fear of infection.<br>− CHWs continued their treatment of children during the outbreak, but there was a decrease in the number treated at the peak of the outbreak.<br>− CHWs also carried out community education, contract tracing, and active case finding.<br>− CHWs reported that utilization reduced because of community members' fear of contracting Ebola while seeking treatment. A smaller number of CHWs reported that the number of children treated increased because people were afraid to go to health facilities.<br>− Some CHWs reported that they stopped treating children because of a directive from the government to stop treating.<br>− Drug stockouts were another reason that CHWs stopped treating children.<br>− CHWs felt confidence in continuing treatment services after the "no touch" iCCM training. However, most CHWs were trained late in the outbreak.
Singh	Evaluating the effectiveness of sexual and reproductive health services during humanitarian crises: A systematic review	2018	London School of Hygiene and Tropical Medicine, World Health Organization	Journal article	PLOS One	Various	Various	Various	Various	Sexual and reproductive health	Various	Community health workers	Literature review	− The review found high-quality evidence for training of lower-level health care providers, the use of CHWs to promote SRH services, a three-tiered network of health workers providing SRH services.<br>− The review found high-quality evidence for community-based SRH education and CHW delivery of injectable contraceptives.
Smith-Nonini	Primary Health Care and its Unfulfilled Promise of Community Participation: Lessons from a Salvadoran War Zone	1997	University of North Carolina	Journal article	Human Organization	Americas	El Salvador	Conflict	General population	Not specified	Health education, community mobilization, treatment of common illnesses	Lay health promoters	Documentation of experience	− CHWs provided basic healthcare services and health education in communities that did not access to professional health workers during the civil war.
Smith-Nonini	"Popular" health and the state: Dialectics of the peace process in El Salvador	1997	University of North Carolina	Journal article	Social Science and Medicine	Americas	El Salvador	Conflict	General population	Primary healthcare	Health education, curative and preventive services (not specified)	Lay health promoters	Documentation of experience	− During the war, community-based lay health promoters were the key health providers in rebel-held areas.

Soe	International non-governmental organizations' provision of community based tuberculosis care for hard-to-reach populations in Myanmar, 2013–2014	2017	Myanmar Department of Medical Research, Institute of Tropical Medicine, Chinese Center for Disease Control and Prevention	Journal article	Infectious Diseases of Poverty	South-East Asia	Myanmar	Conflict	General population	Infectious diseases	Health education, detection of suspected TB cases, DOTS for TB, counseling on treatment adherence	Community health volunteers	Analysis of quantitative program data, document review	− Community health volunteers were recruited and managed by NGOs to provide community-based TB care.<br>− The community-based TB control program in challenging contexts was seen as successful.
Speakman	Development of the Community Midwifery Education initiative and its influence on women's health and empowerment in Afghanistan: a case study	2014	London School of Hygiene and Tropical Medicine, Rumi Consultancy, Royal Tropical Institute, Kabul Medical University	Journal article	BMC Women's Health	Eastern Mediterranean	Afghanistan	Conflict	General population	Maternal health	Antenatal care, assisted home delivery, postnatal care, newborn care, provision of contraceptives	Community midwives	Qualitative interviews, document review	− Increase in the number of midwives has been reported as a key contributor to the decrease in maternal mortality in Afghanistan.<br>− Midwives were seen as positive role models for women and girls.<br>− Women were empowered by providing training and economic opportunity to midwives and by prioritizing women's health in communities.<br>− Sustainability issues included security, corruption, quality-maintenance, and financing.<br>− Security issues challenged deployment, demand, and data collection, which led to inequitable maternal healthcare provision, weakened data reliability, and potentially underestimated mortality.<br>− It was suggested that community midwifery could be made safer and more attractive through enhanced salaries, a higher ratio of midwives to population in insecure areas, negotiated protection with local leaders.
Steege	Gender and Community Health Worker programmes in fragile and conflict-affected settings: Findings from Sierra Leone, the Democratic Republic of Congo and Liberia	2018	Liverpool School of Tropical Medicine	Report	N/A	Africa	Democratic Republic of Congo, Liberia, Sierra Leone	Conflict, disease outbreak	General population	Maternal health, newborn health, child health	Not specified	Community health workers, community health assistants	Document review, qualitative interviews, photography, community mapping	− Even though women were prioritized for CHW recruitment, it was often to find women who had the minimum educational/literacy requirements.<br>− Crises may increase gender inequity in CHW hiring practice because disruptions in education make it difficult for women to obtain minimum education requirements.<br>− Women's household responsibilities may increase even more during times of conflict, when men may be more likely to be absent from the home.<br>− Community-based selection of CHWs may favor men because of gender norms.<br>− Male family members may prevent women from taking work as a CHW.<br>− In insecure settings, women may face greater risk to travel long distances unescorted. Cultural norms may also restrict women's travel.<br>− Women rarely become peer supervisors because of lower literacy and educational levels.

Stehling-Ariza	The impact of active surveillance and health education on an Ebola virus disease cluster — Kono District, Sierra Leone, 2014–2015	2016	Centers for Disease Control and Prevention, University of New South Wales, Sierra Leone Ministry of Health and Sanitation, International Federation of Red Cross and Red Crescent Societies	Journal article	BMC Infectious Diseases	Africa	Sierra Leone	Disease outbreak	General population	Infectious diseases	Community-based surveillance, health education	Community health workers	Qualitative interviews, analysis of quantitative program data, record review	− CHWs carried out active disease surveillance and health education in their communities.<br>− CHWs were part of a surveillance team that contributed to the reduced time from onset of disease to reporting of suspected cases.
Stone	Community Event-Based Surveillance for Ebola Virus Disease in Sierra Leone: Implementation of a National-Level System During a Crisis	2016	International Rescue Committee, Save the Children, Sierra Leone Ministry of Heath and Sanitation, CARE International, ABC Development	Journal article	PLOS Currents Outbreaks	Africa	Sierra Leone	Disease outbreak	General population	Infectious diseases	Community-based surveillance	Community health monitors	Qualitative interviews, analysis of surveillance data, knowledge test	− Community health monitors were in charge of detecting triggers and reporting them to the Community Surveillance Supervisor.<br>− Community health monitors were active in the community through visits to households and speaking with key informants.<br>− Because of logistical delays, it took six months for the system to be fully operational and it began reporting well after the peak in caseload.<br>− Monthly reporting was high, with over 80% reporting in most months.<br>− Most alerts were for deaths that did not meet the criteria of a trigger event.<br>− Lack of communication, logistical constraints, and physical barriers all presented obstacles to the community health monitors.<br>− Community ownership of the program was important because of the sensitive nature.
Tanabe	Piloting community-based medical care for survivors of sexual assault in conflict-affected Karen State of eastern Burma	2013	Women's Refugee Commission, Global Health Access Program, Burma Medical Association, Karen Department of Health and Welfare	Journal article	Conflict and Health	South-East Asia	Myanmar	Conflict	General population	Sexual and reproductive health	Medical care for survivors of sexual assault	Community health workers, traditional birth attendants	Focus group discussions	− CHWs were trained to provide care to victims of sexual assault.<br>− No cases of sexual assault were seen or heard of by any of the CHWs during the study period.<br>− CHWs were comfortable with the topic of GBV.<br>− CHWs provided training to TBAs to provide community information on sexual assault care.<br>− CHWs and TBAs were amongst the groups that community members in Karen State reported to trust to seek medical care.

Tanabe	Family planning in refugee settings: findings and actions from a multi-country study	2017	Women's Refugee Commission, University of Michigan, United Nations High Commissioner for Refugees	Journal article	Conflict and Health	Africa, Eastern Mediterranean, South-East Asia, Western Pacific	Bangladesh, Djibouti, Jordan, Kenya, Malaysia, Uganda	Conflict	General population, camp	Reproductive health	Provision of contraceptives, community education	Community health workers	Household survey, facility assessment, qualitative interviews, focus group discussions	− CHWs provided contraceptives to refugees.<br>− CHW provision of Sayana Press was piloted in a refugee camp in Uganda.<br>− CHWs also carried out community education and mobilization on family planning.<br>− Refugees viewed CHWs as one of the most appropriate sources of information on family planning.
Tanaka	Refugee participation in health relief services during the post-emergency phase in Tanzania	2004	University of Tokyo	Journal article	Public Health	Africa	Tanzania	Conflict	Camp	Child health, infectious diseases	Health education, home visits, immunization, nutrition assessment, distribution of contraceptives, treatment of diarrhea, DOTS for tuberculosis, disease surveillance	Health information team members	Household survey, qualitative interviews, focus group discussions	− Refugees were trained to provide health education in a refugee camp.<br>− A large majority (79%) of refugees said they learned about preventing illnesses from the CHWs.
Tappero	Lessons Learned during Public Health Response to Cholera Epidemic in Haiti and the Dominican Republic	2011	U.S. Centers for Disease Control and Prevention	Journal article	Emerging Infectious Diseases	Americas	Haiti	Disease outbreak	General population	Infectious diseases	Community education	Community health workers	Documentation of experience	− CHWs were trained to carry out community education on cholera prevention.

Teela	Community-based delivery of maternal care in conflict-affected areas of eastern Burma: Perspectives from lay maternal health workers	2009	Johns Hopkins Bloomberg School of Public Health, Global Health Access Program, Burma Medical Association	Journal article	Social Science and Medicine	South-East Asia	Myanmar	Conflict	General population	Maternal health	Emergency obstetric care, blood transfusion, focused antenatal and postnatal care, family planning promotion, provision of contraceptives	Maternal health workers, lay health workers, traditional birth attendants	Qualitative interviews, focus group discussions, case studies	− Non-resident MHWs provided essential maternal health interventions including emergency obstetric care.<br>− MHWs provided five of the six components of basic emergency obstetric care, plus blood transfusion.<br>− MHWs were able to provide, at the community level, services normally considered feasible only in facility-based settings.<br>− MHWs trained and worked with less skilled lay health workers and traditional birth attendants, who were closer to the community than MHWs and provided less technical services.<br>− Positive relationships between MHWs and other project providers (HWs and TBAs), village leaders and community members were critical for success of the project.<br>− MHWs' demonstration of their clinical abilities was also critical to acceptability.<br>− When access to some areas were blocked due to military presence, only the local workers (HWs and TBAs) were able to provide services in the communities.<br>− MHWs suggested solutions to some logistical constraints, including the use of walkie-talkies for communication, donkeys for travel and transport of supplies, and headlamps for improved lighting needed during provision of services.
Terry	Brigadistas and revolutionaries: Health and Social Justice in El Salvador	2013	N/A	Book chapter	Rutgers University Press	Americas	El Salvador	Conflict	General population	Not specified	First aid, trauma care, assisting surgeries, general medical care, health education	Brigadistas de salud	Documentation of experience	− Communities working independent of the government established their own health systems, using mostly CHWs.
Thara	An assessment of post-tsunami psychosocial training programmes in Tamilnadu, India	2008	Schizophrenia Research Foundation	Journal article	International Journal of Social Psychiatry	South-East Asia	India	Natural disaster	General population	Mental health	Psychosocial support	Community level workers	Qualitative interviews	− Community level workers were trained to provide psychosocial support to tsunami survivors.<br>− Community level workers reported to have repetitive training programmes and redundancy in training.<br>− Many community level workers did not implement their training knowledge because the trainings occurred many months after the tsunami when the services were no longer seen as necessary.

Thormar	The impact of disaster work on community volunteers: The role of peri-traumatic distress, level of personal affectedness, sleep quality and resource loss, on post-traumatic stress disorder symptoms and subjective health	2014	University of Amsterdam, Arq Psychotrauma Expert Group, University of Innsbruck, Reykjavík University	Journal article	Journal of Anxiety Disorders	South-East Asia	Indonesia	Natural disaster	General population	Disaster response	Remove the deceased, rescue the trapped and/or injured, re-establish water and sanitation, distribute food and non-food items, work in a public kitchen or aid warehouse, handle logistics, first aid, psychosocial support, locate missing community members	Red Cross volunteers	Survey of volunteers	− Red Cross volunteers who responded to an earthquake experienced trauma from the earthquake, as well as additional trauma as volunteers. As a result they experienced high levels of post-traumatic stress disorder.
Thormar	Organizational factors and mental health in community volunteers. The role of exposure, preparation, training, tasks assigned, and support	2013	University of Amsterdam, Centrum'45, University of Innsbruck, Reykjavik University	Journal article	Anxiety, Stress and Coping	South-East Asia	Indonesia	Natural disaster	General population	Disaster response	Remove the deceased, rescue the trapped and/or injured, re-establish water and sanitation, distribute food and non-food items, work in a public kitchen or aid warehouse, handle logistics, first aid, psychosocial support, locate missing community members	Red Cross volunteers	Survey of volunteers	− Loss of resources was the strongest contributor to symptoms of mental health complaints.<br>− The task of evacuating bodies was not related to symptoms, but the tasks of providing PSS, handling administration, or handing out food aid were strongly related to symptoms.<br>− Community volunteers that had a high need for support and the lack of support from team leaders and the organization strongly contributed to depression.<br>− In order to assist volunteers in maintaining optimal mental health, organizations should minimize exposure by managing working hours, fluctuating tasks between those that provide low and high reward, supplementing lost resources (e.g., sleeping facilities), providing proper equipment that takes into account the volunteers safety, and strengthening organizational support.
Thormar	Evaluation of the Ebola response - Uganda	Not specified	International Federation of the Red Cross and Red Crescent Societies, Ugandan Red Cross	Report	N/A	Africa	Uganda	Disease outbreak	General population	Infectious diseases	Disease surveillance, contact tracing, follow-up of Ebola survivors, community sensitization to reduce stigma against survivors, community education	Red Cross Volunteers	Qualitative interviews, focus group discussions	− During an Ebola outbreak, Red Cross volunteers carried out disease surveillance, contact tracing, follow-up of recovered Ebola survivors, community sensitization to reduce stigma against survivors, and community education.<br>− Stakeholders reported that the volunteers' status as community members allowed them to play a key role in passing key messages to community members.

Tiffany	Community-Based Surveillance to Monitor Mortality in a Malaria-Endemic and Ebola-Epidemic Setting in Rural Guinea	2016	Médecins sans Frontières, Guéckédou Direction Préfectorale de la Santé, Guinea National Malaria Control Program, University of Basel, Lausanne University Hospital, Epicentre	Journal article	American Society of Tropical Medicine and Hygiene	Africa	Guinea	Disease outbreak	General population	Mortality surveillance	Community-based mortality surveillance, malaria case management	Community volunteers	Analysis of surveillance data	− Community volunteers were trained to carry out community-based mortality surveillance.
Tuhkanen	A disaster preparedness and response project in Afghanistan: participants' perceptions	2008	Helsinki Polytechnic Stadia, HAMK University of Applied Sciences, University of Ulster	Journal article	Journal of Advanced Nursing	Eastern Mediterran ean	Afghanist an	Conflict, natural disaster	General population	Disaster preparedness	Not specified	Community-based first aid volunteers	Qualitative interviews, focus group discussions	− Respondents said that community volunteers needed to be integrated into disaster preparedness and response activities. Volunteers could support emergency mobile units with their community knowledge and contacts.

United Nations Children's Fund	Flexible Humanitarian – Development Programming: Case Studies	2014	United Nations Children's Fund	Report	N/A	Africa, Eastern Mediterranean	Ethiopia, Mali, Pakistan	Conflict, nutrition emergency, natural disaster	General population	Nutrition, not specified	Treatment of childhood malnutrition, malnutrition screening	Health Extension Workers, community health workers	Documentation of experience	Ethiopia:<br>− Frequent nutrition emergencies were treated as short-term emergencies. Therefore, the response was initiated each time an emergency occurred and was dependent on humanitarian funding and the response was short-term. This caused shortages in funding and delays in emergency response.<br>− The government decided to include management of SAM in it's development programming.<br>− CMAM was also initiated to allow HEWs to treat malnutrition in the community, which led to the greatest improvements in access to treatment.<br>− These changes led to much higher numbers of malnutrition treatments, high cure rates, improved and faster emergency response, and consistent funding and emergency preparedness.<br>− Costs associated with phasing in and phasing out of emergency programs were also eliminated.<br>− Integration of malnutrition management into the health system also allowed for longitudinal data on malnutrition cases for the majority of the country, compared to the limited data provided formerly by period nutrition surveys in limited areas.<br><br>Mali:<br>− Lack of emergency preparedness and capacity for emergency response led to a slow response to the conflict and nutrition emergency in 2012.<br>− CHWs shifted their focus to nutrition during a nutrition emergency.<br>− Scale-up of nutrition interventions by CHWs during an emergency period led to these interventions being included in the regular development programming post-emergency.<br>− Maintaining a long-term focus during the emergency and flexible funding allowed for longer-term planning so that interventions could span humanitarian and development contexts. For example, humanitarian resources were invested in health system strengthening activities, which facilitated the transition back to development programming.<br><br>Pakistan<br>− The Lady Health Workers were critical to the flood response, as they were re-tasked from their more development-oriented work to focus on life-saving activities (e.g. malnutrition screening and delivery of a basic life-saving package).

United Nations Children's Fund	Children in Pakistan: Every Child's Right - Responding to the Floods in Pakistan	2010	United Nations Children's Fund	Report	N/A	Eastern Mediterran ean	Pakistan	Natural disaster	General population, camp	Maternal health, newborn health, child health, nutrition	Treatment of childhood illnesses, immunization, de-worming, health education, promotion of exclusive breastfeeding	Lady health workers	Documentation of experience	− Lady Health Workers were supported to continue their usual MNCH services in affected areas.<br>− They were also critical in carrying out mass immunization, de-worming, and health education campaigns.
van der Hoek	Early diagnosis and treatment of malaria in a refugee population in Sri Lanka	1997	University of Leiden, Anti-Malaria Campaign, University of Sri Jayawardenepura	Journal article	Southeast Asian Journal of Tropical Medicine and Public Health	South-East Asia	Sri Lanka	Conflict	Camp	Child health	Treatment of malaria, health education	Health volunteers	Household survey, qualitative interviews	− Pre-existing health volunteers were quickly trained on malaria case management in IDP camps.<br>− Only 45% of community respondents were aware of the presence of the health volunteers.
Vandi	Ebola and community health worker services in Kenema District, Sierra Leone: please mind the gap!	2017	Sierra Leone Ministry of Health and Sanitation, Institute of Tropical Medicine, University of Toronto, Dignitas International, International Rescue Committee, St. Michael's Hospital	Journal article	Public Health Action	Africa	Sierra Leone	Disease outbreak	General population	Child health	Treatment of childhood pneumonia, diarrhea, and malaria	Community health workers	Analysis of quantitative program data	− The % of CHWs reporting any activity increased from 59% before the outbreak to 95% during the outbreak, and was sustained at 98% after the outbreak. The increased reporting rate was likely due to improved supervision during the outbreak.<br>− The number of children treated for malaria increased during the outbreak. This was the case in chiefdoms with and without Ebola cases. This was likely due to the shift from RDTs to presumptive treatment of fever with antimalarials.<br>− The number of children treated for pneumonia and diarrhea decreased during the outbreak. The decrease in pneumonia treatments was seen in chiefdoms with and without EVD cases. In contrast, only chiefdoms with EVD cases experienced a decrease in diarrhea treatments. After the outbreak, the numbers rebounded to or above the pre-outbreak levels.
Vijayakuma r	CASP – An intervention by community volunteers to reduce suicidal behaviour among refugees	2017	Sneha, Voluntary Health Services, Samarth, Christian Medical College, Adventist Development Relief Agency	Journal article	International Journal of Social Psychiatry	South-East Asia	India	Conflict	Camp	Mental health	Psychosocial support	Community volunteers	Household survey, focus group discussions	− An intervention using community volunteers to provide psychosocial support reduced suicidal behaviour among refugees.

Viswanathan	Can community health workers increase coverage of reproductive health services?	2011	Johns Hopkins University, The GAVI Alliance, Afghanistan Ministry of Public Health	Journal article	Journal of Epidemiology and Community Health	Eastern Mediterranean	Afghanistan	Conflict	General population	Reproductive health	Home visits; provision of micronutrient supplementation and anti-malarials to pregnant women; distribution of contraceptives; diagnosis and treatment of malaria, diarrhea and acute respiratory infection; health education; support for immunization; promotion of facility delivery; assisting in preparing for an emergency referral	Community health workers	Household survey	− The presence of a female CHW in the community was associated with higher use of modern contraception, antenatal care services and skilled birth attendants, but presence of a male CHW was not.
von Roenne	Reproductive health services for refugees by refugees: an example from Guinea	2010	Gesellschaft für Technische Zusammenarbeit, Reproductive Health Group, London School of Hygiene and Tropical Medicine, Institute of Tropical Medicine	Journal article	Disasters	Africa	Guinea	Conflict	Camp	Reproductive health	Health promotion, provision of contraceptives	Reproductive health facilitators	Documentation of experience	− Community-based reproductive health facilitators provided information and advice on reproductive health, distributed contraceptives, encouraged pregnant women to attend ANC, couples to use family planning, or those experiencing sexually transmitted infection symptoms to seek treatment.
Weiss	Community-based mental health treatments for survivors of torture and militant attacks in Southern Iraq: a randomized control trial	2015	Johns Hopkins Bloomberg School of Public Health, Heartland Alliance International, University of Washington	Journal article	BMC Psychiatry	Eastern Mediterranean	Iraq	Conflict	General population	Mental health	Treatment of mental health disorders	Community mental health workers	Randomized controlled trial	− CMHWs in one arm provided a transdiagnostic treatment for multiple mental health disorders, the Common Elements Treatment Approach (CETA). In the other arm, they provided an established, evidence-based cognitive behavior therapy approach, cognitive processing therapy (CPT).<br>− The CETA intervention showed large effect sizes for all outcomes. The CPT intervention showed moderate effects sizes for trauma and depression, with small to no effect for anxiety or dysfunction, respectively.<br>− Non-specialized health workers were able to treat comorbid symptoms of trauma, depression and anxiety, and dysfunction among survivors of systematic violence who have limited access to mental health professionals.<br>− Security issues and connectivity issues presented obstacles in community mental health workers contacting their supervisors.

Williams	Perceptions of Health Communication, Water Treatment and Sanitation in Artibonite Department, Haiti, March-April 2012	2015	Centers for Disease Control and Prevention	Journal article	PLOS One	Americas	Haiti	Disease outbreak	General population	Infectious diseases, WASH	Health education, distribution of water treatment products	Community health workers	Focus group discussions	− CHWs were trusted by the community to provide reliable health messages, verify the quality of water, and provide water treatment products.
Wilner	Sustained use in a relief-to-recovery household water chlorination program in Haiti: comparing external evaluation findings with internal supervisor and community health worker monitoring data	2017	Tufts University School of Medicine, Centers for Disease Control and Prevention, Tufts University, Deep Springs International	Journal article	Journal of Water, Sanitation and Hygiene for Development	Americas	Haiti	Natural disaster	General population	WASH	Provision of water treatment, monitoring of water treatment, health education	Community health workers	Analysis of quantitative program and evaluation data	− CHWs performed household visits to distribute safe storage containers and chlorine products and to monitor household water treatment status and provide health education.<br>− The program achieved high and sustained water treatment coverage.
Wilunda	Barriers to Institutional Childbirth in Rumbek North County, South Sudan: A Qualitative Study	2016	Kyoto University, Doctors with Africa CUAMM, Tenri Health Care University, World Health Organization	Journal article	PLOS One	Africa	South Sudan	Conflict	General population	Maternal health	Assisted home delivery	Traditional birth attendants	Qualitative interviews, focus group discussions	− Reasons that women preferred to deliver at home with a TBA instead of in a health facility included: long distance to the health facility and cost of travel, preference for TBAs who follow traditional cultural practices, negative perceptions of delivery practices at health facilities (such as childbirth position and vaginal examination).

Wilunda	Changing the role of traditional birth attendants in Yirol West County, South Sudan	2017	Kyoto University, Doctors with Africa CUAMM, Tenri Health Care University, World Health Organization	Journal article	PLOS One	Africa	South Sudan	Conflict	General population	Maternal health	Referral of women to health facilities for childbirth, prenatal visits, postnatal visits, first aid	Traditional birth attendants	Qualitative interviews, focus group discussions	− When national guidelines were changed to instruct TBAs to refer all women to deliver in health facilities, community- based TBAs' main task became referring women to health facilities for childbirth.<br>− TBAs were mostly complying and referring women to facilities. However, some TBAs were still attending deliveries in the community and making referrals only when there was a complication.<br>− The new role of TBAs was mostly accepted by women in the community because of a gradual realization that institutional childbirth was safer than home childbirth.<br>− TBA's motivation and commitment were key to integrating them into the health system, despite poor monetary compensation.<br>− As TBAs were mostly illiterate, they relied on facility-based CHWs to help them complete monthly reports.<br>− Insecurity made it difficult for TBAs to escort women to health facilities, especially at night because they feared inter-tribal fighting, rape, and abduction.<br>− Lack of mobile phones and money to make calls and lack of basic items like flashlights, bags, raincoats and boots made it more difficult to arrange for referrals to health facilities.<br>− Some TBAs without any intention of assisting women to deliver at home found themselves doing so because of various barriers including those faced by women in accessing health care.<br>− Long distances to health facilities and lack of transportation was a major obstacle for childbirth at health facilities.<br>− TBAs were not receiving enough monetary compensation to meet their basic needs.

Wisborg	Life or death. The social impact of paramedics and first responders in landmine-infested villages in northern Iraq	2008	Hammerfest Hospital, Trauma Care Foundation Iraq, Tromsø Mine Victim Resource Center, Bodø University College	Journal article	Rural and Remote Health	Eastern Mediterran ean	Iraq	Conflict	General population	Trauma	Trauma care	Village first responders	Qualitative interviews, focus group discussions	− A system of village paramedics and lay first responders for landmine injuries was highly appreciated by the communities.<br>− Community members wanted the paramedics and first responders to address issues beyond landmine injuries, and expanding the program's scope was important for gaining the trust of the communities.<br>− The program increased the feeling among community members that they were important to the external society.<br>− Community services were seen as reducing the likelihood that community members would migrate from the villages.
Witter	The gendered health workforce: mixed methods analysis from four fragile and post-conflict contexts	2017	ReBUILD Consortium, Queen Margaret University, Makerere School of Public Health, University of Sierra Leone, Biomedical Research and Training Institute, Cambodian Development Resource Institute, Liverpool School of Tropical Medicine	Journal article	Health Policy and Planning	Africa, Western Pacific	Cambodia, Sierra Leone, Uganda, Zimbabwe	Conflict	General population	Not specified	Not specified	Community health workers, traditional birth attendants	Stakeholder mapping, document review, qualitative interviews, life histories, analysis staffing data, health worker survey	During the Ebola outbreak in 2014–15, women predominated amongst the lowest cadres of health workers who were been critical to the Ebola response—including volunteers, traditional birth attendants, community-based motivators and community health workers. Thus, gender norms shaped vulnerability to Ebola.
Woodward	Reproductive health for refugees by refugees in Guinea IV: Peer education and HIV knowledge, attitudes, and reported practices	2011	London School of Hygiene & Tropical Medicine, Reproductive Health Group, Gesellschaft für Internationale Zusammenarbeit, Institute of Tropical Medicine, Institute of Tropical Medicine and International Health	Journal article	Conflict and Health	Africa	Guinea	Conflict	Camp	Maternal health	Provision of contraceptives, health education	Lay health workers	Survey of men and women	− Refugee lay health workers provided contraceptives and health education in refugee camps
Yusufzai	Health workers murdered as Pakistan vaccinates 8.4 million children	2014	Not specified	Commentary	Canadian Medical Association Journal	Eastern Mediterran ean	Pakistan	Conflict	General population	Child health, infectious diseases	Polio immunization, health education, distribution of hygiene kits	Vaccination workers	Documentation of experience	− Community members participated in large-scale polio immunization campaigns, as well as health education and distribution of hygiene kits.<br>− Vaccination workers have been targeted by militants, with many having been killed.<br>− Security was improved by having police guard the vaccination teams.

Appendix S3: Results of peer-reviewed article database search, grey literature search, and reference screening

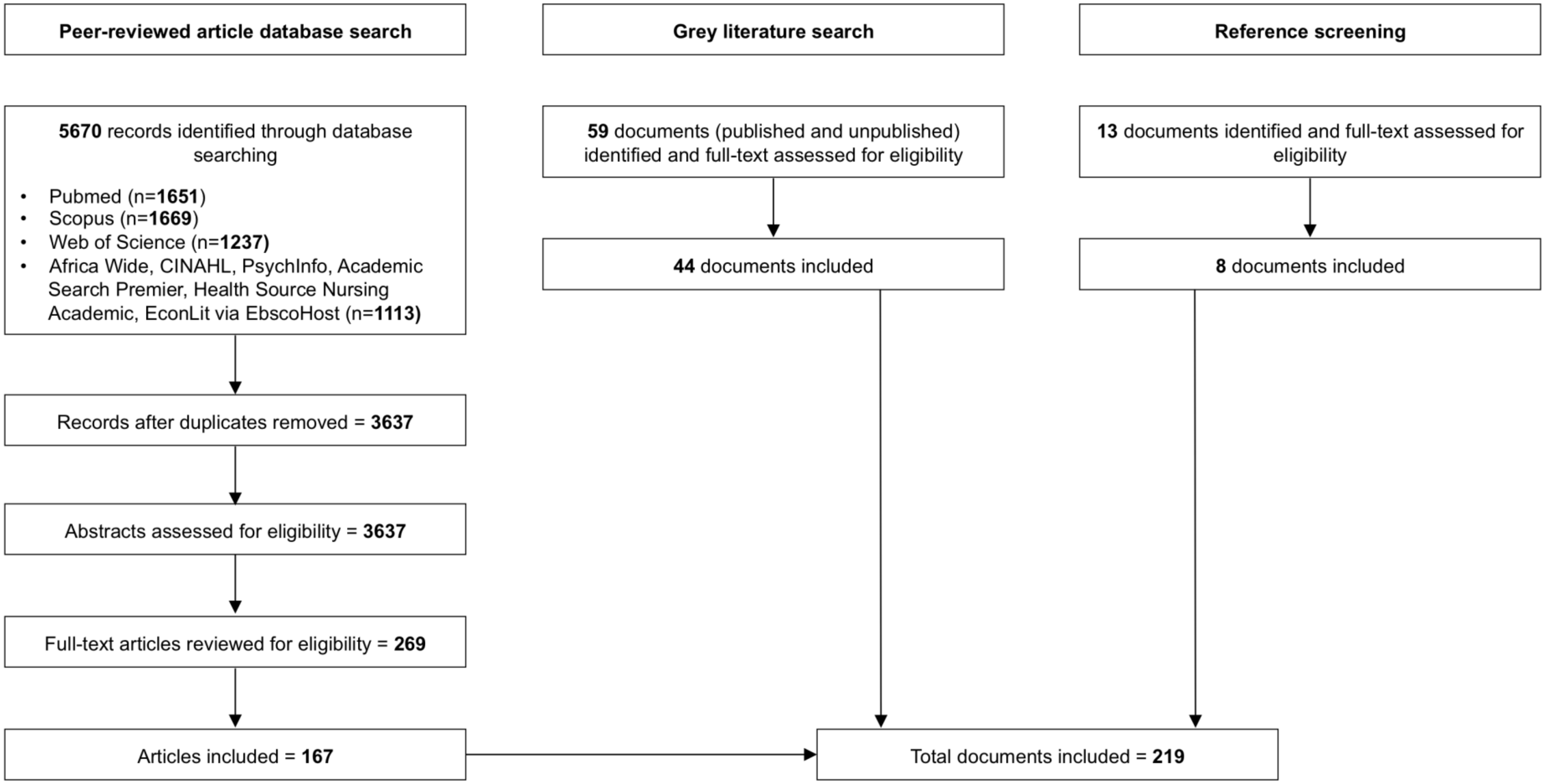

Supplement: Online Supplementary Document [file jogh-10-020602-s001.pdf]
